# Multi-dimensional machine learning approaches for fruit shape phenotyping in strawberry

**DOI:** 10.1093/gigascience/giaa030

**Published:** 2020-04-30

**Authors:** Mitchell J Feldmann, Michael A Hardigan, Randi A Famula, Cindy M López, Amy Tabb, Glenn S Cole, Steven J Knapp

**Affiliations:** 1 Department of Plant Sciences, University of California, Davis, 1 Shields Ave, Davis, CA 95616, USA; 2 USDA-ARS-AFRS, 2217 Wiltshire Rd, Kearneysville, WV 25430, USA

**Keywords:** *Fragaria × ananassa*, fruit shape, morphometrics, latent space phenotypes, machine learning, principal progression of *k* clusters

## Abstract

**Background:**

Shape is a critical element of the visual appeal of strawberry fruit and is influenced by both genetic and non-genetic determinants. Current fruit phenotyping approaches for external characteristics in strawberry often rely on the human eye to make categorical assessments. However, fruit shape is an inherently multi-dimensional, continuously variable trait and not adequately described by a single categorical or quantitative feature. Morphometric approaches enable the study of complex, multi-dimensional forms but are often abstract and difficult to interpret. In this study, we developed a mathematical approach for transforming fruit shape classifications from digital images onto an ordinal scale called the Principal Progression of *k* Clusters (PPKC). We use these human-recognizable shape categories to select quantitative features extracted from multiple morphometric analyses that are best fit for genetic dissection and analysis.

**Results:**

We transformed images of strawberry fruit into human-recognizable categories using unsupervised machine learning, discovered 4 principal shape categories, and inferred progression using PPKC. We extracted 68 quantitative features from digital images of strawberries using a suite of morphometric analyses and multivariate statistical approaches. These analyses defined informative feature sets that effectively captured quantitative differences between shape classes. Classification accuracy ranged from 68% to 99% for the newly created phenotypic variables for describing a shape.

**Conclusions:**

Our results demonstrated that strawberry fruit shapes could be robustly quantified, accurately classified, and empirically ordered using image analyses, machine learning, and PPKC. We generated a dictionary of quantitative traits for studying and predicting shape classes and identifying genetic factors underlying phenotypic variability for fruit shape in strawberry. The methods and approaches that we applied in strawberry should apply to other fruits, vegetables, and specialty crops.

## Background

Fruit breeders actively selected several morphological and quality phenotypes during the domestication of the garden strawberry (*Fragaria × ananassa*), an allo-octoploid (2n = 8x = 56) of hybrid origin [1–3]. *F. × ananassa* was created in the early 1700s by interspecific hybridization between ecotypes of wild octoploid species (*Fragaria virginiana* and *Fragaria chiloensis*), multiple subsequent introgressions of genetic diversity from *F. virginiana* and *F. chiloensis* subspecies in subsequent generations, and artificial selection for horticulturally important traits among interspecific hybrid descendants. Domestication and breeding have altered the fruit morphology, development, and metabolome of the garden strawberry, distancing modern cultivars from their wild progenitors [4–9]. Approximately 300 years of breeding in the admixed hybrid population has led to the emergence of high-yielding cultivars with large, firm, visually appealing, long shelf-life fruit that can withstand the rigors of harvest, handling, storage, and long-distance shipping [10]. Fruit shape is an essential trait of agricultural products, particularly those of specialty crops, owing to perceived and realized relationships with the quality and value of the products. Image-based fruit phenotyping has the potential to increase scope, throughput, and accuracy in quantitative genetic studies by reducing the effects of user bias, enabling the analysis of larger sample sizes, and more accurate partitioning of genetic variance from environments, management, and other non-genetic sources of variation [11–13].

Many fruit phenotyping approaches rely on the human eye to sort fruit into discrete, descriptive categories for planar (2D) shapes (e.g., rhombic and reniform) [14–19]. Categories are either nominal [11, 20, 21], existing in name only, or ordinal, referring to a position in an ordered series or on a gradient [15, 16, 21]. Classification into categories is often labor-intensive and prone to human bias, which can increase with task complexity and time requirements [22, 23]. Alternative scoring approaches rely on morphometrics and machine learning to automate classification; e.g., sorting fruit into shape categories in both tomato [11] and strawberry [20]. Unsupervised machine learning methods (e.g., *k*-means clustering), unlike supervised methods, are useful for pattern detection and clustering, while supervised machine learning methods (e.g., support vector machines) are useful for prediction and classification [24, 25]. Unsupervised clustering enables the calculation of several measures of model performance and overfitting to balance compression and accuracy. However, the categories derived from these techniques are without order, resulting in the need for a suitable transformation to an ordinal scale more appropriate for quantitative genetic analyses [26–30]. In this context, ordinal categories give the interpretation of relationship with, or distance from, other shape categories in a series. To enable this interpretation, we developed a method for asserting the progression through fruit shape categories derived from unsupervised machine learning methods. The Principal Progression of *k* Clusters (PPKC) allowed us to non-arbitrarily determine the appropriate shape gradient for statistical analyses using empirical data. The advantages of PPKC, relative to a manually determined ordinal scale, are that it does not require arbitrary, a priori decisions and is unsupervised, which obviates additional operator bias. Here, we describe approaches for translating digital images of strawberries into computationally defined phenotypic variables for identifying and classifying fruit shapes.

Fruit shape and anatomy are complex, multi-dimensional, and, potentially, abstract phenotypes that are often not completely or intuitively described by planar descriptors and individual qualitative or quantitative variables. Beyond the qualitative definitions used in plant systematics [18, 20], references to fruit shape encompass a wide variety of mathematical parameters and geometric indices that establish quantitative measurements of plant organs [19, 31–33]. Much like human faces or grain yield, fruit shape and anatomy are products of the underlying genetic and non-genetic determinants of phenotypic variability in a population [34, 35]. Quantitative phenotypic measurements have allowed researchers to uncover some of the genetic basis of fruit shape in tomato [36, 37], pepper [38, 39], pear [40], melon [35], potato [41], and strawberry [9, 42]. However, the major genetic determinants of fruit shape remain unclear, or understudied, in octoploid strawberry, in part because researchers have not yet translated fruit shape attributes into holistic, quantitative variables, which may empower the identification of underlying genes or quantitative trait loci through genome-wide association studies (GWAS) and other quantitative genetic approaches [43–46]. Quantitative features often rely on linear metrics of distance (e.g., height, width, and perimeter) and are generally modified into compound descriptors that remove the effects of size (e.g., aspect ratio or roundness) [40, 42, 47]. However, compound linear descriptors often have limited resolution compared to more comprehensive, multivariate descriptors [33]. Elliptical Fourier analysis (EFA) quantifies fruit shape from a closed outline by converting a closed contour into a weighted sum of harmonic functions [12, 48–51]. Generalized Procrustes analysis (GPA) quantifies the distance between sets of biologically homologous, or mathematically similar, landmarks on the surface of an object [48, 51–57]. Fruit shape can also be described using linear combinations of pixel intensities from digital images extrapolating from analyses generally used to quantify color patterns and facial recognition [13, 58–63]. Similar pixel-based descriptors have recently been referred to as ”latent space phenotypes” and arise from unsupervised analyses (i.e., principal component analysis [PCA] and auto-encoding neural networks) that allow a computer to produce novel, independently distributed features directly from images [64, 65]. Here, we generate a dictionary of 68 quantitative features, including linear-, outline-, landmark-, and pixel-based descriptors to investigate the quality of different features in preparation for quantitative genetic analyses.

**Figure 1:**
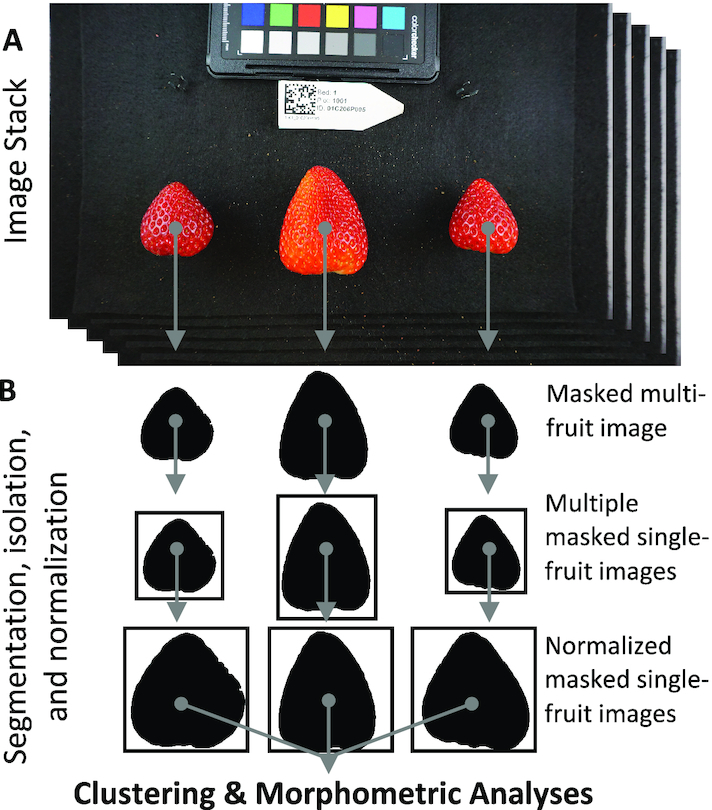
An example of the processing pipeline. **(A)** A user collects a stack of images containing multiple strawberries and a unique QR code. **(B)** All images are then segmented using the SIOX algorithm implemented in ImageJ. Each object is then cut from its original image based on the coordinates of its bounding rectangle in R 3.5.3. White pixels are then added to the edges of each frame until all images are 1,000 × 1,000 pixels. Regions of interest are then scaled such that the major axis of each object becomes 1,000 pixels in ImageJ. Output images are scale invariant and maintain the original aspect ratio.

**Figure 2:**
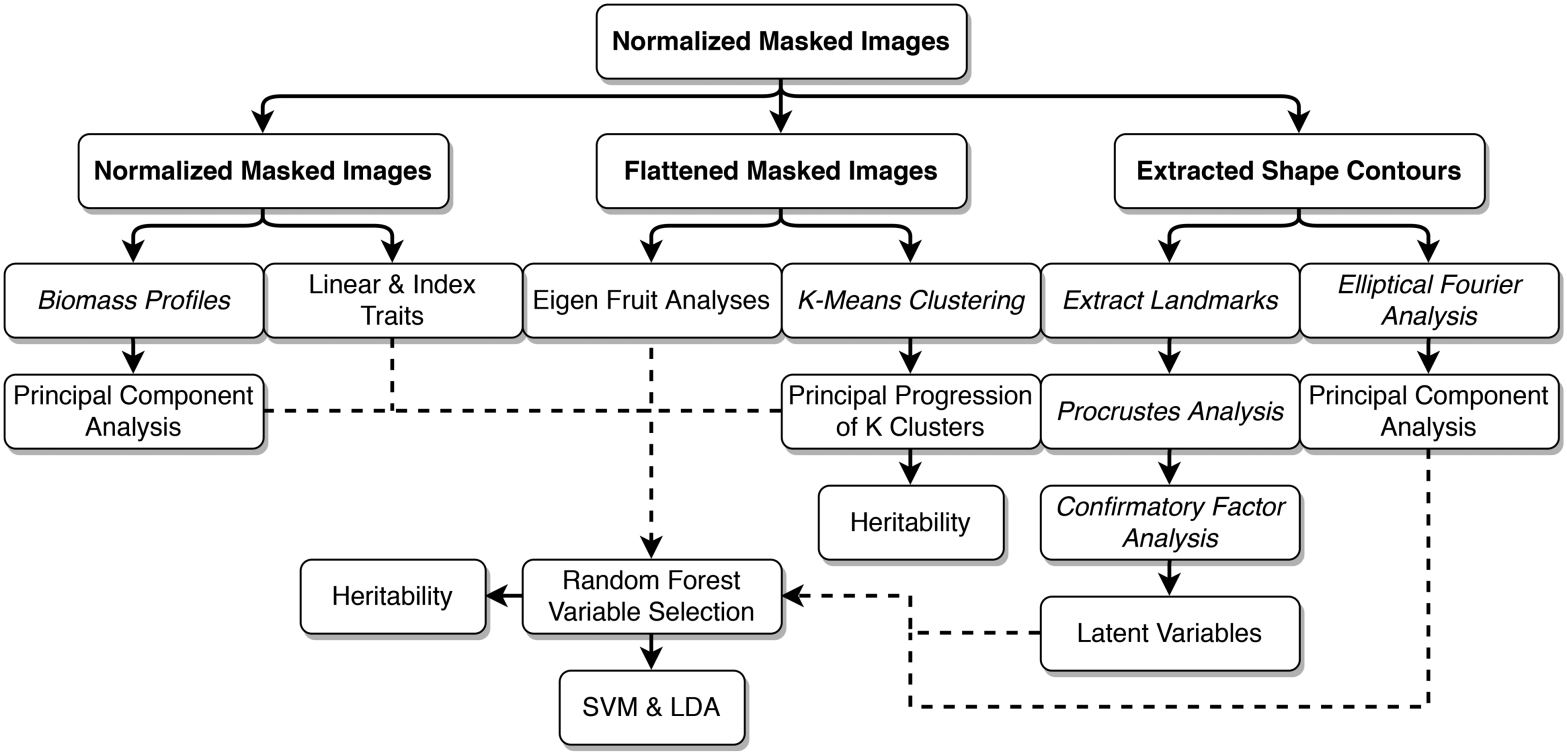
Analysis pipeline for this study. All images start as normalized, binary images from Fig 1. Images then follow each of the paths through different morphometric feature extractions including linear geometric features, biomass profile analysis (BPA), EigenFruit analysis, Procrustes analysis, and elliptical Fourier analysis as either normalized or flattened images (e.g., linear, BPA, and EigenFruit analysis) or as shape contours (e.g., GPA and EFA). Flattened binary images are used to perform *k*-means clustering and subsequently PPKC.

The ultimate goal of our study was to develop heritable phenotypic variables for describing fruit shape, which could then be used to identify the genetic factors underlying phenotypic differences in fruit shape. The phenotyping and analytic workflow for this study are summarized in Figs 1 and 2. We first describe and demonstrate the application of PPKC, which transforms categories discovered from unsupervised machine learning methods to a more convenient and analytically tractable ordinal scale [26, 28, 29]. We then explore the relationship between machine-acquired categories and 68 quantitative features extracted from digital images. Next, we apply random forest regression to select critical sets of quantitative features for classification and use supervised machine learning methods, including support vector regression (SVR) and linear discriminant analysis (LDA), to determine the accuracy of shape classification. We discovered that there are only a few categories of interest in a highly domesticated breeding population and that a small number of features are needed to classify shape into the discovered categories accurately. We also find that ordinal shape categories are highly heritable and that the features needed for accurate classification are also heritable.

## Data Description

The data released with this article contain digital images of 6,874 strawberry fruit from 572 hybrids originating from the University of California, Davis, Strawberry Breeding Program. The data for this article, including pre-processed images (Fig. 1A), processed images (Fig. 1B), and extracted features (see Methods, Fig. 2), are available on Zenodo [66]. The pre-processed images typically contained multiple berries per image along with a data matrix bar code indicating the genotype ID and other elements of the experiment design. The processed images are 1,000 × 1,000 pixels-scaled binary images of individual fruit. The extracted features data set is provided as a CSV file. The code to replicate the analyses in this article is provided in a GitHub repository [67]. Additionally, snapshots of the code and data supporting this work are available in the GigaScience repository, GigaDB [68]. We hope that the release of these data assists others in developing novel morphometric approaches to better understand the genetic, developmental, and environmental control of fruit shape in strawberry, and more broadly in other fruits, vegetables, and specialty crops.

## Analyses

### *k*-Means clustering

*k*-Means clustering rapidly detects patterns in large, multi-dimensional data sets used for clustering, decision making, and dimension reduction [24, 69, 70]. It is an iterative algorithm that partitions a data set into a pre-defined number of non-overlapping clusters, *k*, by minimizing the sum of squared distances from each data point to the cluster centroid. A centroid corresponds to the mean of all points assigned to the cluster. Here, we used *k*-means to cluster flattened binary images (Fig. 1; see Methods). Individual fruits were segmented from the image background as a binary mask, normalized by the major axis, resized to 100 × 100 pixels, and flattened into a vector (Figs 1 and 2; see Methods). We represented each image as a 10,000-element vector containing binary pixel values. We were able to rapidly and reliably assign images to classes using *k*-means clustering. In this experiment, we allowed *k*, the number of permitted categories, to range from 2 to 10. This range was chosen because we anticipate that a human-based classification system would not have the speed or reliability needed for this task, particularly for larger values of *k*.

### Principal progression of k clusters

**Figure 3:**
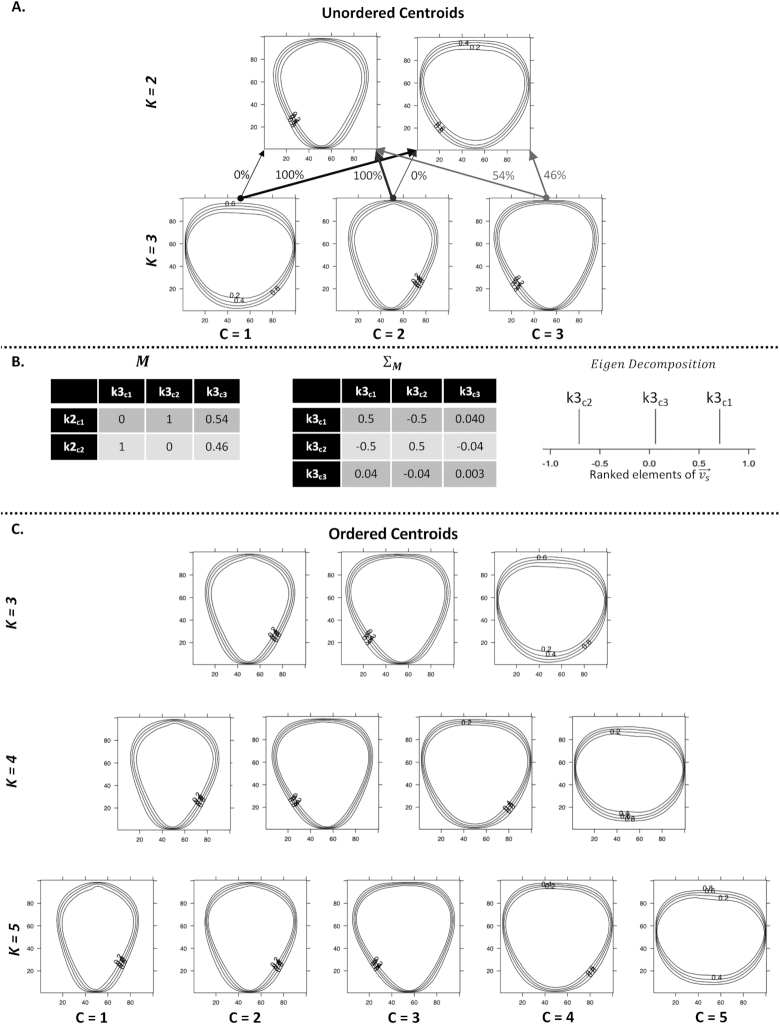
An example use of PPKC. **(A)** After *k*-means clustering is performed clusters are randomly assigned a numeric value (1,2,...,*k*). When *k* > 2, this value becomes nominal. PPKC relies on the fact that the order through clusters when *k* = 2 has identical interpretations in either direction. The lines representing each clusters centroid reflect the 20th, 40th, 60th, and 80th quantiles, moving out from the center of each image. **(B)***Left*, A table representation of the resultant matrix from Equation (1). Each cell represents the proportion of images in the column class and in the row class, normalized by the number of images in the column class. **(B)***Middle*, A table representation of $\mathbf {\Sigma _M}$. **(B)***Right*, The ranked elements of $\vec{v}_s$ shown on a number line. **(C)** After using PPKC, the order of groups is explicitly identified. In this example, showing *k* = [3, 5], the order discovered seems to trend from tall and thin berries, through more triangular shapes, ending with berries that are short and wide.

*k*-Means clustering does not assign a progression or gradient to discovered classes. However, score and ordinal traits are typically more useful and are more common in quantitative genetic studies than nominal scales [26, 28, 29, 71]. We developed a new method to transform the categories derived from *k*-means onto an ordinal scale, which we call PPKC (Fig. 3; Algorithm 1). This method relies on *k*-means clustering to categorize images and can be used to discover an appropriate ordinal scale in nominal data empirically. *k*-Means supports several metrics for evaluating model performance and overfitting, including adjusted *R*^2^, Akaike information criterion (AIC), and Bayesian information criterion (BIC), which allows users to determine the most appropriate value of *k* given the observed data. The gradient between clusters was estimated by performing PCA on a covariance matrix reflecting the structured relationship between a focal cluster and all previously discovered clusters.

We first assign each flattened binary image (Fig. 1) to a category using a *k*-means approach. We assign a cluster to each image and allow the number of clusters, *k*, to range from 2 through 10. The order is subsequently inferred using PPKC (Fig. 3, Algorithm 1). When *k* = 2, the order of relatedness is considered arbitrary, and both *k*2_*c*1_ → *k*2_*c*2_ and *k*2_*c*2_ → *k*2_*c*1_ have the same meaning, where ”→” indicates the progression of discovered categories. Any given order and its reverse are considered equivalent, and this applies to higher levels of *k* as well; e.g., the hypothetical ranking of clusters 1, 4, 2, 3 is considered equivalent to 3, 2, 4, 1 because the relative relationship between the *k* clusters is identical in both (e.g., *c*3 is more related to *c*2 than either *c*1 or *c*4). For each cluster of interest (e.g., *k*4_*c*1_, *k*4_*c*2_, *k*4_*c*3_, and *k*4_*c*4_), we calculate the proportion of each cluster that came from *k*3_*c*1_, *k*3_*c*2_, or *k*3_*c*3_ and *k*2_*c*1_ or *k*2_*c*2_ (i.e., all former classifications). These proportions enable the estimation of similarity between a focal cluster (e.g., *k*4_*c*1_) and the clusters of all prior values of *k*. We then normalize the proportions by the total number of images in the focal cluster (e.g., *k*4_*c*1_, *k*4_*c*2_, *k*4_*c*3_, and *k*4_*c*4_) (Equation 1).

For every level of *k* > 2, we construct $\mathbf {M}$, a rectangular matrix of size $(k^2-k)/2 -1$ × *k*(Algorithm 1 line 13). The sum of each column should equal *k* − 2. The proportions are continuous values in the range [0, 1] that described the origin of a particular focal cluster (e.g., *k*4_*c*1_) as it relates to the clusters of *k* = 3 and *k* = 2 or all clusters [2, *k* − 1]. In the following example, *k* = 4:

(1) $$\begin{equation*}
\mathbf {M} = \begin{bmatrix}\displaystyle\frac{ |k4_{c1} \wedge k3_{c1}|}{ |k4_{c1}|} & \displaystyle\frac{ |k4_{c2} \wedge k3_{c1}|}{ |k4_{c2}|} & \displaystyle\frac{ |k4_{c3} \wedge k3_{c1|}}{ |k4_{c3}|} & \displaystyle\frac{ |k4_{c4} \wedge k3_{c1}|}{ |k4_{c4}|}\\
\displaystyle\frac{ |k4_{c1} \wedge k3_{c2}|}{ |k4_{c1}|} & \displaystyle\frac{ |k4_{c2} \wedge k3_{c2}|}{ |k4_{c2}|} & \displaystyle\frac{ |k4_{c3} \wedge k3_{c2|}}{ |k4_{c3}|} & \displaystyle\frac{ |k4_{c4} \wedge k3_{c2}|}{ |k4_{c4}|}\\
\displaystyle\frac{ |k4_{c1} \wedge k3_{c3}|}{ |k4_{c1}|} & \displaystyle\frac{ |k4_{c2} \wedge k3_{c3}|}{ |k4_{c2}|} & \displaystyle\frac{ |k4_{c3} \wedge k3_{c3|}}{ |k4_{c3}|} & \displaystyle\frac{ |k4_{c4} \wedge k3_{c3}|}{ |k4_{c4}|}\\
\displaystyle\frac{ |k4_{c1} \wedge k2_{c1}|}{ |k4_{c1}|} & \displaystyle\frac{ |k4_{c2} \wedge k2_{c1}|}{ |k4_{c2}|} & \displaystyle\frac{ |k4_{c3} \wedge k2_{c1|}}{ |k4_{c3}|} & \displaystyle\frac{ |k4_{c4} \wedge k2_{c1}|}{ |k4_{c4}|}\\
\displaystyle\frac{ |k4_{c1} \wedge k2_{c2}|}{ |k4_{c1}|} & \displaystyle\frac{ |k4_{c2} \wedge k2_{c2}|}{ |k4_{c2}|} & \displaystyle\frac{ |k4_{c3} \wedge k2_{c2|}}{ |k4_{c3}|} & \displaystyle\frac{ |k4_{c4} \wedge k2_{c2}|}{ |k4_{c4}|} \end{bmatrix}
\end{equation*}$$

We then calculate the variance-covariance matrix of Equation (1) (Algorithm 1 line 18). The variance-covariance matrix, $\mathbf {\Sigma _M}$, represents the relationship between each focal cluster (e.g., *k*4_*c*1_, *k*4_*c*2_, *k*4_*c*3_, or *k*4_*c*4_).

(2) $$\begin{equation*}
\mathbf {\Sigma _M} = \begin{bmatrix}\sigma _{k4_{c1}}^2 & \sigma _{k4_{c2}, k4_{c1}} & \sigma _{k4_{c3}, k4_{c1}} & \sigma _{k4_{c4}, k4_{c1}}\\
\sigma _{k4_{c1}, k4_{c2}} & \sigma _{k4_{c2}}^2 & \sigma _{k4_{c3}, k4_{c2}} & \sigma _{k4_{c4}, k4_{c2}}\\
\sigma _{k4_{c1}, k4_{c3}} & \sigma _{k4_{c2}, k4_{c3}} & \sigma _{k4_{c3}}^2 & \sigma _{k4_{c4}, k4_{c3}}\\
\sigma _{k4_{c1}, k4_{c4}} & \sigma _{k4_{c2}, k4_{c4}} & \sigma _{k4_{c3}, k4_{c4}} & \sigma _{k4_{c4}}^2 \end{bmatrix}
\end{equation*}$$

We then perform eigen decomposition on Equation (2) using the following equation (Algorithm 1 line 19).

(3) $$\begin{equation*}
\mathbf {\Sigma _M} = \mathbf {V}\mathbf {\Lambda } \mathbf {V}^{-1} .
\end{equation*}$$

In Equation (3), $\mathbf {\Lambda }$ is a diagonal matrix with values corresponding to the *k* eigenvalues of $\mathbf {\Sigma _M}$ and $\mathbf {V}$ is a square matrix containing eigenvectors associated with the eigenvalues in $\mathbf {\Lambda }$. We then extract the eigenvector associated with the largest eigenvalue, $\vec{v}_{\lambda _{\mathrm{max}}}$. We order the elements of $\vec{v}_{\lambda _{\mathrm{max}}}$ such that the resultant vector, $\vec{v_s}$, has the property $v_{s_1} \le ... \le v_{s_k}$. We do not consider the distance between elements in $\vec{v_s}$, only their rank. The clusters are then indexed to match the rank of the associated elements in $\vec{v_s}$. There are at most *k* eigenvalues associated with eigenvectors of length *k* due to $\mathbf {\Sigma _M}$ being *k* × *k*. Eigen decomposition is used to describe the major axis of variance in $\mathbf {\Sigma _M}$. In theory, this perspective of covariance should be able to separate the classes effectively because it describes a linear axis containing the greatest amount of independent variation and solutions are non-arbitrary. The value a category takes on this composite axis is therefore suggestive of its linear relationship to other the *k* categories being considered. However, we note that relationships containing branches, bubbles, and other topological features will not be captured accurately. In this study, we are unable to report a visually meaningful order when *k* ≥ 9 (Fig. S1) [66]. The change in progression could be reflective of overfitting the number of groups in *k*-means clustering. The large change of slope at *k* = 4 in the total within-group sums of squares, AIC, and adjusted *R*^2^ evidenced overfitting (Fig. S2) [66]. The strongest evidence for 4 clusters is in the BIC, which is minimized when *k* = 4 (Fig. S2D) [66]. The elements of $\vec{v_s}$ tend to converge on one another as *k* increases, which may be indicative of little biological information in the new clusters and overfitting (Fig. S3) [66]. Given that only relatively small covariance matrices are considered in this algorithm, the computational time to order *k* = [3, ..., 10] on an early 2015 MacBook Pro 2.9 GHz Core i5 with 8GB memory is <0.2 seconds.

**Algorithm 1:**
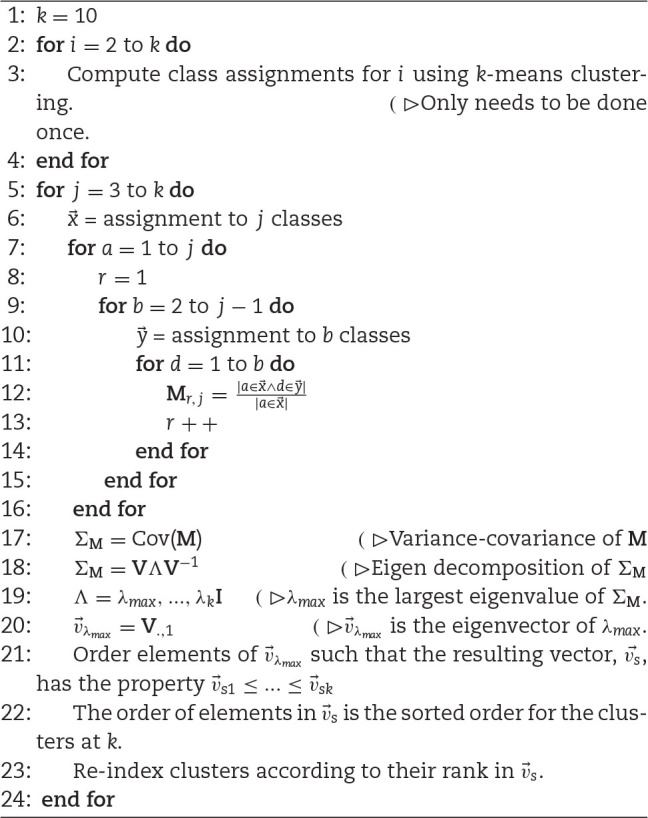
Principal Progression of K Clusters (PPKC) Algorithm

### Broad-sense heritability of ordered categories

For each value of *k*, broad-sense heritability (*H*^2^) on an entry-mean basis was assessed using a general linear mixed model with a cumulative logit link function ( Equations 4 and 5) [72]. For this data set, *H*^2^ was generally high, ranging from *H*^2^ = 0.80 to 0.98, even as *k* → 10 (Table 2). These estimates of *H*^2^ are very similar to those reported by Antanaviciute [16] (i.e., *H*^2^ = 0.84). When the *H*^2^ of a trait is in this range, it indicates that independent replications of the same individuals share a high degree of similarity and that most of the variation among individuals originated from genetic variation among individuals. Because the plant material used in this study came from genetic clones, any variation in fruit shape among replicates originated from random, unobserved effects. For *k* ≥ 9, the accuracy of *H*^2^ estimates is expected to be lower than for *k* ≤ 8 because the gradient of the phenotype seems to be improperly specified. In this set of germplasm, we propose a set of 4 primary classes for categorizing fruit shape (Fig. 3 and S2) [66]. As *k* increases from 5 to 10, the visual similarity of some clusters is high (Fig. S1) [66], thus indicating fewer relevant delineations (Fig. S3) [66]. As indicated, there is strong evidence in these data that there are 4 distinct clusters in these data (Fig. S2) [66].

### Feature selection using random forests

**Figure 4:**
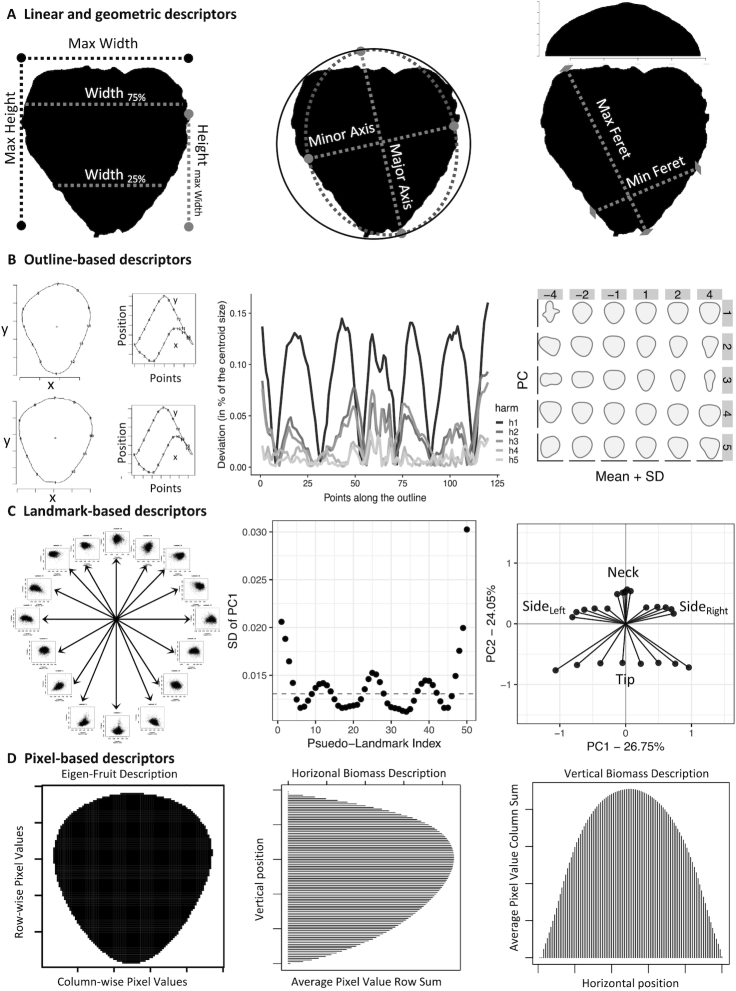
Trait dictionary for this study. **(A)** Linear descriptors. *Left*, Simple linear measurements. *Center*, Best-fit ellipse axes. For the circle, Round and Circ = 1. *Right*, Maximum and minimum Feret. Histogram represents the marginal distribution on the horizontal axis used to calculate Var, Skew, and Kurt. **(B)** Outline descriptors. *Left*, The 2 leftmost images are the outlines of 2 strawberries with 12 evenly spaced points. The graphs on the right show the original closed outline as 2 oscillating functions. *Center*, Deviations from the closed outline with increasing harmonics (harm = [*h*1, *h*5]).*Right*, The plot shows the effects of PC [1,5] (vertical) with effect sizes, [−4, 4] (horizontal) on the mean shape. **(C)** Landmark descriptors. *Left*, 50 evenly spaced landmarks are extracted and treated as bi-variate features.*Center*, Standard deviation of PC1 for each landmark is plotted in sequence. Dashed horizontal line is the median standard deviation (SD). *Right*, Pseudo-landmarks were selected to represent each region of high variance. Using the values on the first principal axis as observed variables, confirmatory factor analysis was performed to infer latent relationships to tip, left and right side, and neck shape. **(D)** Pixel descriptors. *Left*, Mean EigenFruit using flattened binary images. *Center*, Mean horizontal biomass using image row sums. *Right*, Mean vertical biomass using image column sums.

**Figure 5:**
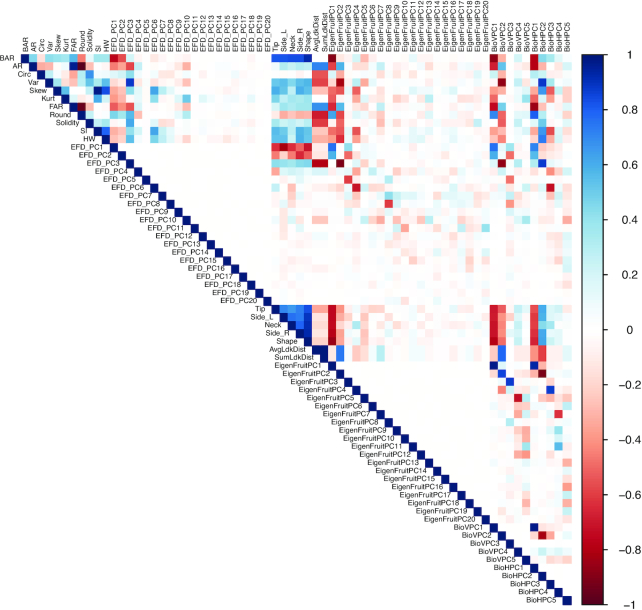
Correlations between all 68 features used in this study. Blue indicates positive correlations, and red, negative correlations.

**Figure 6:**
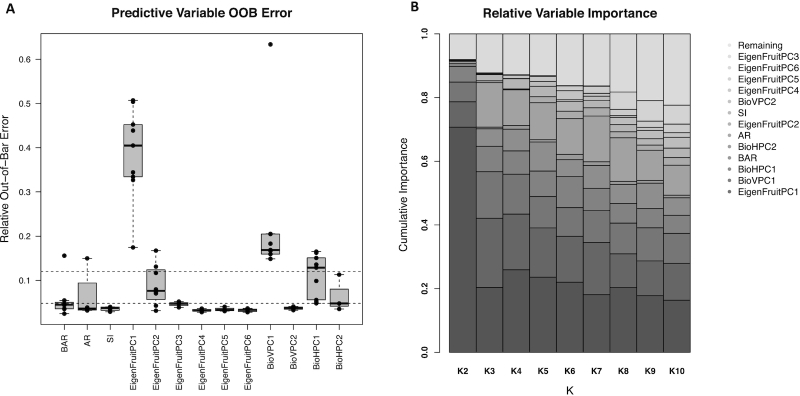
**Results from feature selection. (A)** Out-of-bag error for each of the 13 selected features. Horizontal dashed lines are the median (0.047) and mean (0.12) OOB. For each trait shown, the lower vertical dashed line is the first quartile, the lower boundary of the gray box to the horizontal black line is the second quartile, the horizontal black line to the upper boundary of the gray box is the third quartile, and the upper dashed line is the fourth quartile. Points not in the quartile range are considered outliers. **(B)** The relative importance of each feature within each level of *k*. The 13 selected features explain >80% of the weight attributed to all of the features, excluding *K* = 9 and 10.

**Figure 7:**
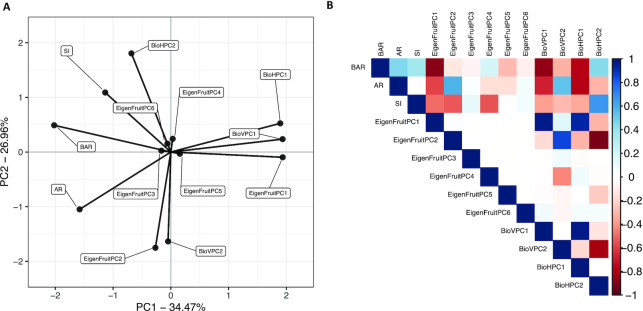
Relationship between selected features. **(A)** Principal directions of the feature variance-covariance matrix among the 13 features selected for classification. **(B)** Pearson correlation matrix of the 13 selected features. Blue indicates positive correlations, and red, negative correlations.

To discover which of 68 quantitative features (summarized in Figs 4 and 5) capture and reflect differences in shape categories, supervised machine learning was used to estimate feature importance (see Methods) [73]. Of the 68 features used as predictors in a random forest regression (see Methods), we selected only 13. Out-of-bag (OOB) error is an estimate of how poorly models perform when a specific feature is excluded and is akin to error estimated from jackknife resampling (Fig 6). In this way, features with higher estimates tend to be more relevant for classification and prediction. In this experiment, features could only be selected up 9 times, once per value of *k*. We maintained features that were selected in ≥3 levels of *k* to use as independent variables in classification (Table 1). The 13 selected features accounted for >80% of importance assigned to the 68 features across all values of *k* (Fig 6B). Here, the use of ”EigenFaces,” an analysis from the 1980s, designed to classify human faces, was re-purposed for the quantification and classification of fruit shape in strawberry [58–61]. Pixel-based features dominated the selected features and include principal components (PCs) 1−7 of the EigenFruit analysis (EigenFruitPC_[1, 6]_), PCs 1 and 2 of the vertical biomass profile (BioVPC_[1, 2]_), and PCs 1 and 2 of the horizontal biomass profile (BioHPC_[1, 3]_) (Table 1 and Figs 6 and 7). These features originated from the same data type as used in *k*-means clustering (i.e., pixel intensities), which is likely the reason they make up the majority of the selected features (Table 1 and Figs 6 and 7). Several geometric descriptors were also selected, including the bounding aspect ratio (BAR), shape index (SI), and ellipse aspect ratio (AR) (Table 1 and Figs 6 and 7). We generated a subset of 5 features with mean OOB ≥ 0.047 (Fig. 6A). OOB = 0.047 was the median OOB error for all features across all classes. This subset of features included EigenFruitPC_[1, 2]_, BioVPC_1_, and BioHPC_[1]_ (Table 1). We also generated a third smaller set that included only EigenFruitPC_1_, BioVPC_1_, and BioHPC_1_ with mean OOB ≥ 0.12 (Fig. 6A). OOB = 0.12 was the mean OOB error for all features across all classes. The prevalence of pixel-based descriptors in these selected subsets indicated the magnitude of relevant shape information that they described.

**Table 1:** Broad-sense heritability of selected features

Feature	*H* ^2^	*k* Selected	Normalized eigenvalue $_{(80\%, 50\%, 20\%)}$	Feature set
EigenFruit PC1	0.68	9	0.26 _(0.27, 0.27, 0.26)_	13, 5, 3
EigenFruit PC2	0.58	8	0.14 _(0.14, 0.14, 0.14)_	13, 5
EigenFruit PC3	0.00	3	0.05 _(0.06, 0.05, 0.06)_	13
EigenFruit PC4	0.69	5	0.04 _(0.04, 0.05, 0.04)_	13
EigenFruit PC5	0.43	4	0.03 _(0.03, 0.04, 0.03)_	13
EigenFruit PC6	0.47	5	0.03 _(0.03, 0.03, 0.03)_	13
Vertical biomass profile PC1	0.67	9	0.65 _(0.66, 0.66, 0.66)_	13, 5, 3
Vertical biomass profile PC2	0.49	4	0.17 _(0.17, 0.16, 0.17)_	13
Horizontal biomass profile PC1	0.65	9	0.44 _(0.44, 0.46, 0.44)_	13, 5, 3
Horizontal biomass profile PC2	0.62	3	0.36 _(0.36, 0.35, 0.37)_	13, 5
Bounding aspect ratio	0.71	8	*NA*	13
Shape index	0.72	4	*NA*	13
Ellipse aspect ratio	0.58	4	*NA*	13

Broad-sense heritability (*H*^2^) estimated on a per-line basis.

*k* selected is the number of classification models that a feature was selected in, out of 9 (i.e., *k* = [2, 10]).

Normalized eigenvalues is the eigenvalue associated with a specific PC divided by the sum of all eigenvalues.

The large value is the normalized eigenvalue from the full data set. Values in parentheses contain the normalized eigenvalues for the 80%, 50%, and the 20% training sets, respectively.

Feature set indicates in which of the 3 sets a given feature was included.

### Broad-sense heritability and relationship of selected features

While the continuous nature of the morphometric features is expected to be more conducive and provide higher resolution to quantitative genetic analyses compared to their categorical counterparts, it is also vital that these features be heritable. The *H*^2^ for each feature was estimated on a clone-mean basis using a linear mixed-effects model (see Equations 5 and 6) [74]. The *H*^2^ for each feature is reported in Table 1. Estimates of *H*^2^ for the quantitative features ranged from low (>0.3) to high (>0.7). Heritability estimates were consistent with those previously reported for shape phenotypes in strawberry and other plant species [12, 42, 75].

Fig. 7A shows the directions of the feature variance-covariance matrix with the traits labeled as in Fig. 6. Fig. 7B shows the correlation matrix between the 13 selected features. For the 5 features selected by OOB error (Fig. 6), indicated with a "5" in Table 1, the estimated *H*^2^ was ≥0.58. Because the majority of selected features are PCs of different pixel-based analyses (Fig. S5) [ 66], there were many weak correlations (Fig.   7B). We hypothesize that the importance of these features is partly driven by the similarity of the raw data (i.e., binary pixel intensities) used in *k*-means clustering to acquire shape categories and for EigenFruit shape analysis. Although PCs are uncorrelated, we observed strong correlations between PCs from different analyses (Fig. 7). EigenFruitPC_1_ shared a strong positive correlation with both BioVPC_1_ and BioHPC_1_ (ρ = 0.98; *P* < 2E−16 and ρ = 0.93; *P* < 2E−16, respectively), as did EigenFruitPC_2_ with BioVPC_2_ (ρ = 0.86; *P* < 2E−16). BioHPC_2_ was negatively correlated with both EigenFruitPC_2_ and BioVPC_2_ (ρ = −0.92;*P* < 2E−16 and ρ = −0.81;*P* < 2E−16, respectively). BioHPC_3_ was negatively correlated with EigenFruitPC_4_ (ρ = −0.87; *P* < 2E−16). BAR was negatively correlated with EigenFruitPC_1_, BioVPC_1_, and BioHPC_1_ (ρ = −0.89;*P* < 2E−16, ρ = −0.87; *P*< 2E−16, and ρ = −0.78; *P* < 2E−16, respectively). Reported *P*-values were Bonferroni adjusted for all 78 pairwise comparisons between the 13 selected features ). The correlations between these features indicated that the pixel-based descriptors describe comparable patterns of phenotypic variation.

### Image classification using selected features

The accuracy of classification, or prediction, is typically assessed by cross-validation [24, 76]. We generated training sets that consisted of 80% (5,500), 50% (3,437), or 20% (1,374) of the images. Assignment to either training or test set was random and without stratification. It is possible that stratification would be needed for more iterations, >10, smaller sample sizes, or very unequal images per *k* category. *k*-means clustering was performed using the training sets, and *k* was allowed to range from 2 to 10. We assigned the test set images to the nearest neighboring cluster for each level of *k*. We performed PPKC on the clusters derived from the training set, and the similarity between the full set and training sets was visually assessed. The clusters derived from the different sets appeared to be nearly identical (Fig. S6) [66]. The order of clusters derived from the reduced data set also appears identical to those described in the full set (Fig. S6) [66]. The PC-based features were recalculated using the training data sets and the corresponding test set images projected into the new space. We only extracted the 13 selected features. These included EigenFruitPC_[1, 6]_, BioVPC_[1, 2]_, and BioHPC_[1, 2]_ (Table 1). The selected geometric features, including BAR, SI, and AR, were not recalculated because they do not change concerning the other samples, unlike *k*-means and PCA, which both rely on and change on the basis of observed data. For EigenFruitPC_[1, 6]_, BioVPC_[1, 2]_, and BioHPC_[1, 2]_, the percent variance explained by each feature was similar to that in the full data set (Table 1), indicating that the PCs derived from the reduced set describe similar features of shape as those derived from the full set.

SVR and LDA were both used for classification (see Methods). We performed 10 iterations of each set size and feature set across all levels of *k*. The results of this experiment are reported in Table 2. Overall, the models performed with high accuracy of classification. Generally, as we used fewer features for classification, model performance was reduced, most notably for larger values of *k*. Indeed, when *k* = 2 accuracy improved slightly with fewer features in the different models. In general, SVR was found to outperform LDA consistently. LDA only outperformed SVR with very small training sets relative to the test set (Table 2). Using 5 features for classification, we achieved the highest accuracy (99.5%) for *k* = 2. In the range of interest, *k* = [2, 4], the models did not fall below 90.0% accuracy for any training set size.

**Table 2:** Classification model evaluations validation experiment

Set _(Train/Test)_	*k*	*H* ^2^	Accuracy_13_	Precision_13_	Recall_13_	FPR_13_	Accuracy_5_	Precision_5_	Recall_5_	FPR_5_	Accuracy_3_	Precision_3_	Recall_3_	FPR_3_
80/20	2	0.98	0.990/0.978	0.990/0.978	0.990/0.978	0.010/0.022	0.995/0.982	0.995/0.983	0.995/0.981	0.005/0.019	0.990/0.983	0.990/0.983	0.990/0.985	0.010/0.015
	3	0.87	0.985/0.963	0.985/0.962	0.982/0.957	0.009/0.019	0.990/0.971	0.990/0.973	0.989/0.969	0.003/0.014	0.941/0.910	0.938/0.906	0.926/0.904	0.030/0.046
	4	0.85	0.982/0.949	0.982/0.953	0.981/0.943	0.008/0.020	0.982/0.950	0.983/0.952	0.981/0.951	0.008/0.018	0.946/0.921	0.950/0.935	0.934/0.896	0.019/0.029
	5	0.81	0.973/0.942	0.979/0.949	0.975/0.941	0.009/0.013	0.976/0.955	0.977/0.962	0.980/0.954	0.008/0.010	0.932/0.893	0.939/0.917	0.928/0.879	0.020/0.030
	6	0.83	0.973/0.943	0.976/0.947	0.973/0.940	0.008/0.010	0.965/0.926	0.966/0.934	0.965/0.919	0.010/0.015	0.898/0.852	0.903/0.876	0.889/0.835	0.020/0.031
	7	0.83	0.966/0.941	0.968/0.947	0.966/0.940	0.006/0.010	0.951/0.910	0.952/0.922	0.950/0.904	0.010/0.016	0.870/0.824	0.880/0.857	0.866/0.815	0.021/0.030
	8	0.82	0.963/0.928	0.964/0.934	0.962/0.926	0.009/0.010	0.866/0.825	0.856/0.828	0.858/0.812	0.018/0.027	0.790/0.748	0.790/0.765	0.778/0.731	0.028/0.038
	9	0.80	0.954/0.920	0.956/0.926	0.954/0.917	0.009/0.010	0.828/0.789	0.825/0.801	0.827/0.781	0.021/0.030	0.745/0.715	0.751/0.736	0.741/0.707	0.030/0.038
	10	0.81	0.951/0.909	0.952/0.915	0.951/0.906	0.008/0.010	0.798/0.752	0.798/0.770	0.802/0.752	0.024/0.026	0.708/0.679	0.718/0.704	0.706/0.676	0.034/0.036
50/50	2		0.990/0.978	0.990/0.978	0.990/0.979	0.010/0.021	0.993/0.983	0.992/0.983	0.993/0.983	0.007/0.017	0.990/0.990	0.990/0.990	0.990/0.990	0.010/0.010
	3		0.981/0.961	0.981/0.963	0.980/0.958	0.010/0.022	0.988/0.972	0.989/0.974	0.987/0.971	0.006/0.016	0.943/0.907	0.940/0.902	0.934/0.909	0.030/0.047
	4		0.979/0.951	0.980/0.953	0.979/0.944	0.010/0.019	0.981/0.952	0.981/0.955	0.980/0.954	0.010/0.018	0.943/0.920	0.947/0.933	0.927/0.896	0.020/0.030
	5		0.969/0.941	0.972/0.945	0.969/0.938	0.010/0.014	0.969/0.948	0.972/0.955	0.969/0.945	0.010/0.012	0.922/0.885	0.931/0.912	0.916/0.869	0.020/0.032
	6		0.966/0.941	0.967/0.945	0.966/0.939	0.010/0.010	0.961/0.928	0.961/0.935	0.960/0.917	0.010/0.014	0.887/0.856	0.896/0.882	0.879/0.835	0.022/0.030
	7		0.961/0.934	0.961/0.939	0.960/0.931	0.010/0.010	0.933/0.897	0.932/0.906	0.931/0.887	0.011/0.017	0.851/0.818	0.861/0.848	0.845/0.805	0.025/0.031
	8		0.955/0.928	0.957/0.931	0.955/0.923	0.010/0.010	0.872/0.831	0.861/0.832	0.861/0.808	0.017/0.027	0.794/0.759	0.790/0.772	0.776/0.738	0.028/0.037
	9		0.950/0.918	0.950/0.923	0.949/0.910	0.010/0.010	0.836/0.793	0.830/0.799	0.829/0.779	0.021/0.029	0.746/0.718	0.747/0.731	0.731/0.704	0.030/0.038
	10		0.947/0.909	0.949/0.915	0.947/0.904	0.010/0.010	0.802/0.762	0.798/0.774	0.804/0.755	0.022/0.027	0.707/0.693	0.716/0.713	0.705/0.687	0.031/0.034
20/80	2		0.987/0.977	0.987/0.977	0.986/0.977	0.014/0.023	0.990/0.983	0.990/0.983	0.990/0.983	0.010/0.017	0.990/0.986	0.990/0.986	0.990/0.986	0.010/0.014
	3		0.973/0.955	0.975/0.958	0.973/0.950	0.013/0.024	0.982/0.967	0.982/0.966	0.981/0.967	0.010/0.018	0.942/0.906	0.943/0.907	0.939/0.911	0.028/0.047
	4		0.967/0.944	0.971/0.951	0.964/0.938	0.010/0.020	0.973/0.953	0.977/0.955	0.971/0.953	0.010/0.017	0.940/0.921	0.949/0.939	0.921/0.892	0.020/0.030
	5		0.959/0.941	0.963/0.946	0.954/0.936	0.010/0.017	0.953/0.931	0.954/0.939	0.948/0.923	0.012/0.016	0.899/0.875	0.912/0.899	0.883/0.849	0.026/0.033
	6		0.953/0.935	0.958/0.940	0.951/0.935	0.010/0.012	0.937/0.909	0.938/0.917	0.935/0.899	0.012/0.020	0.851/0.835	0.864/0.861	0.837/0.819	0.030/0.034
	7		0.945/0.928	0.950/0.933	0.944/0.925	0.010/0.010	0.902/0.876	0.901/0.885	0.901/0.866	0.016/0.021	0.812/0.804	0.827/0.829	0.799/0.789	0.032/0.034
	8		0.937/0.913	0.938/0.920	0.933/0.914	0.010/0.011	0.829/0.804	0.825/0.810	0.822/0.792	0.023/0.030	0.736/0.733	0.755/0.753	0.722/0.720	0.039/0.040
	9		0.930/0.908	0.933/0.915	0.927/0.903	0.010/0.010	0.808/0.780	0.802/0.788	0.798/0.767	0.024/0.028	0.706/0.707	0.724/0.727	0.688/0.692	0.038/0.038
	10		0.927/0.901	0.930/0.905	0.926/0.896	0.010/0.010	0.794/0.758	0.796/0.781	0.796/0.760	0.023/0.028	0.677/0.681	0.701/0.706	0.670/0.676	0.038/0.036

Set refers to the 80/20, 50/50, or 20/80 training set/test set split. *k* is the number of categorie. *H*^2^ is the broad-sense heritability and was estimated using the full data set. SVR metric/LDA metric is presented for accuracy, precision, recall, and false-positive rate (FPR). Subscripts 13, 5, and 3 indicate the number of selected features in the classification model fit.

## Discussion

As high-throughput phenotyping for external fruit characteristics becomes of interest to specialty crop researchers, we expect that this work will have various applications in both applied and basic plant research [12, 13, 51, 64, 65], intellectual property protection and documentation [77, 78], and waste reduction [20, 79]. Our study showed that strawberry fruit shapes could be robustly quantified and accurately classified from digital images. Most importantly, our analyses yielded quantitative phenotypic variables that describe fruit shape (Fig. 4), arise from continuous distributions, and are moderately to highly heritable (Table 1). We accomplished this by translating 2D, digital images of fruit into categorical and continuous phenotypic variables using unsupervised machine learning and morphometrics. We found that mathematical approaches developed for human face recognition [58, 59] were powerful for strawberry fruit shape phenotyping (Table 1), that unsupervised shape clustering was robust to sample size deviations (Fig. S6) [ 66], and that only a few quantitative features are needed to accurately classify shapes from images (Table   2), indicating a paradigm appropriate for genetic dissection.

Digital plant phenotyping is able to empower quantitative genetic analyses by providing heritable and biologically relevant, latent phenotypes in a cost-effective manner [13, 64, 65, 80, 81]. In many cases, these latent traits are derived from PCA, multi-dimensional scaling (MDS), structured equation modeling (SEM), persistent homology (PH), or auto-encoding convolutional neural networks, which can be exceedingly abstract and difficult to interpret biologically but may also reveal unexpected patterns of phenotypic and genetic variation [12, 13, 19, 24, 51, 59, 61, 75, 82–84]. Many of the features described in this study, along with those reported by Turner et al. [13] (i.e., biomass profile) [12] (i.e., elliptical Fourier PCs and persistent homology PCs) and Gage et al. [65] (i.e., image PCs and convolutional encodings), had high heritability (Table 1) and are exciting targets for future quantitative genetic analyses, including GWAS and genomic prediction, which have been shown to be successful for shape features in recent work in rice (*Oryza sativa* L.) [85], apple (*Malus domestica*) [86], and pear (*Pyrus* spp.) [87]. However, the *H*^2^ of 1 selected feature in this study, EigenFruitPC_3_, was estimated to be 0.00 (Table 1 and Fig. S4) [66]. Similar results were reported in carrot (*Daucus carota* L.) for pixel-based root and shoot features [13], apple (*Malus domestica*) for elliptical Fourier leaf shape features [12], and corn (*Zea mays*) for pixel-based shoot features [65]. Turner et al. [13] attributed the null *H*^2^ of root shape characteristics to low phenotypic variation between the inbred parents and genotype × environment interactions. This pattern, while seemingly present, was not discussed in detail by either Migicovsy et al. [12] or Gage et al. [65]. While there may be many drivers for this pattern, we hypothesize that the null estimate may arise from the pixel-based descriptors describing more complex aspects of fruit or root shape. If the non-genetic component of a multivariate phenotype is large, then performing PCA on that multivariate trait could produce leading PCs that describe mostly non-genetic variance (e.g., environment, management, and residual). However, there are too few reports to adequately determine the likelihood and causal source of this phenomenon.

We empirically derived the shape progression produced in the present study through the application of a new method, PPKC, and used these mathematical categories to interpret the extracted shape features (Algorithm 1 and Fig. 3). Ordinal categorical traits are commonplace in quantitative genetic studies [29, 71], a current standard for phenotyping external fruit characteristics [14, 15, 42], and enable understanding and explanation of complex, latent space plant phenotypes (Figs 6 and 7). PPKC specifically considers the relationship between a cluster at *k* and all clusters for values <*k* as a covariance matrix and projects this *k*-dimensional space to 1 dimension using eigen decomposition. Ordination using dimension reduction techniques, including PCA, correspondence analysis, and MDS has been previously proposed and used in community ecology [ 88]. Theoretically, the eigen decomposition step of PPKC could be replaced with another technique. However, unlike methods using eigen decomposition, which progressively subdivides variation such that the position on the leading axis (i.e., PC1) is fixed regardless of the number of axes examined, the position of samples on MDS axes may change when different dimensions are extracted, making MDS axes arbitrary and without meaning other than a convenient reference [88]. PPKC identified 4 exemplary strawberry shape categories in the population that we studied, which were characterized by a progression from "longer-than-wide" (prolate) to "wider-than-long" (oblate) ( Figs.   3 and S7) [ 66]. This ordinal scale can be used in breeding and research programs as traits of interest, or it can be used to organize and interpret more abstract quantitative features, such as EigenFruitPCs or SEM latent variables, through supervised machine learning algorithms [ 24]. Critically, this gradient agreed with the arbitrarily defined progressions in previous reports [14, 16]. However, unlike previous studies, which suggested using 9 ordinal [14] or 11 nominal shape categories [20], our work presented empirical evidence for a smaller number of mathematically defined shape categories. We determined that *k* = 4 was the appropriate level of complexity on the basis of the visual appearance of the discovered clusters (Fig. 3), high *H*^2^ estimates (Table 2), and the information criteria calculated for the *k*-means models (Fig. S2) [ 66]. Interestingly, PPKC can determine a visually, reasonable phenotypic gradient up to *k* = 8 (Fig. S3) [ 66] despite strong evidence of overfitting for *k* > 4 (Fig. S2) [ 66]. We extrapolate that PPKC should continue to work beyond *k* = 9 so long as new clusters are distinct and do not arise as an artifact of overfitting *k*.

The specific genetic factors that give rise to variation in fruit shape in octoploid, garden strawberry are currently unclear or understudied. The selective pressure exerted on fruit shape in strawberry could have affected large-effect loci, in which case ordinal phenotypic scores are likely to be sufficient for identifying genetic factors affecting fruit shape. Loss- and gain-of-function mutations have played an essential role in identifying genes affecting fruit shape in tomato, a model that has been highly instructive and important for understanding the genetics of fruit shape and enlargement in plants [34–36, 89, 90]. There are striking examples in tomato and other plants where identified genes regulate the development of fruit shape. For example, the *OVATE* gene in tomato regulates the phenotypic transition from round to pear-shaped fruit [91, 92]. If large-effect mutations underlie differences in strawberry fruit shape, the ordinal classification system proposed here should enable the discovery of such effects. Furthermore, quantitative phenotypes were linked to genetic features that interact with large-effect genes, i.e., suppressors of *OVATE (sov)*, through bulk segregant analysis and quantitative trait locus mapping [93]. In woodland strawberry (*Fragaria vesca*), fruit size and shape are linked to the accumulation and complex interaction of auxin, gibberellic acid, and abscisic acid, mediated by the expression and activity of *FveCYP707* and *FveNCED*, as well as other genes [9]. Because of the high *H*^2^ estimates for several of the newly created phenotypic variables (Table 1), we hypothesize that quantitative, latent space phenotypes can yield a more comprehensive understanding of the underlying genetic mechanisms of fruit shape in garden strawberry through GWAS and other quantitative genetic analyses [44, 45, 94]. We anticipate that the analyses in this study will enable us to discover and study the genetic determinants of fruit shape in strawberry and other specialty crops.

## Methods

### Mating and field design

Seventy-five bi-parental crosses were generated by controlled pollination of 30 parents in an incomplete (14 × 16) factorial mating design. These parents were chosen to represent a broad range of phenotypic diversity in the University of California, Davis, strawberry germplasm. A total of 2,800 hybrid progeny were planted at the Wolfskill Experimental Orchard in Winters, CA, in sets of 20 or 40 per family, depending on seedling survival. Twenty percent of the planted materials from each family were randomly selected for further testing. Clones of 545 of the selected 560 progeny were successfully propagated. Twelve bare-root runner plants of each of the 545 progeny and the 30 parents were collected and planted in November 2017 in Salinas, CA, in 4 plant plots as a randomized complete block design with 3 replicates of each genotype.

### Image acquisition

Strawberries were harvested from plots in Salinas, CA, once in April 2018 and again in May 2018. Digital images of up to 3 fruit per plot were imaged using a Sony α-6000 Mirrorless digital camera mounted on a portable copy stand in aperture priority, with the aperture set to f/8. Strawberries with the calyx removed were placed in the frame against a black felt backdrop, along with a QR code identifying the plot, such that the most extensive face was perpendicular to the sensor. Berries were mounted to a set of staples to eliminate any rolling or pitch of the berries. The time to stage a given set of fruit and acquire an image ranged from 1 to 2 min. All images were acquired with a 16−50 mm lens set to 16 mm and positioned ∼16 cm above the base of the copy stand, resulting in images with 97.4 pixels per cm. In total, 2,924 plots were imaged over the 2 harvest dates.

### Image processing

Input files were JPEG images (3,008 × 1,688 pixels) with the strawberries placed in regular positions within a scene. All images were first segmented and converted to binary using the Simple Interactive Object Extraction (SIOX) tool in ImageJ 2.0.0 [95–97] through custom batch scripts. Images that were unsuccessfully segmented were flagged and handled individually to ensure completeness. ImageJ was used to acquire the bounding rectangle of each object of interest. Each object was extracted on the basis of the dimensions of its bounding rectangle using R 3.5.3 [98] and the jpeg package [99]. White pixels were added to the edges of each image such that the resulting image was a square of size max (*H, W*) × max (*H, W*) using the ”magick::image_border()” package [100]. ”magick::image_resize()” was used to scale the images from max (*H, W*) × max (*H, W*) pixels to 1,000 × 1,000 pixels. This method results in binary images that maintain the original aspect ratio with a maximum dimension equal to 1,000 pixels and then resized to 100 × 100 (Fig. 1). In total, the downstream analyses included 6,874 images of individual berries.

### Feature extraction

#### Categorical features

This method afforded clustering decisions based on raw image data instead of the extracted quantitative features. Each image matrix was flattened into a single 10,000 element row vector; all of the samples were then bound together by columns. The resulting matrix for all samples was 6,874 × 10,000. The ”stats::kmeans()” function in R was used to perform *k*-means clustering. Values of *k* (i.e., the number of clusters) ranged from 2 to 10. Assigned clusters were recorded for all values of *k*. Discovered clusters were then ordered using PPKC (Fig. 3). The ordered categories, across the various levels of *k*, became the response for classification experiments. The correct choice of *k* is often ambiguous, with interpretations depending on the shape and scale of the distribution of points in a data set and the desired clustering resolution of the user. In addition, increasing *k* without penalty will always reduce the amount of error in the resulting clustering, to the extreme case of zero error if each data point is considered its own cluster (i.e., when *k* equals the number of data points, *n*). Intuitively then, the optimal choice of *k* will strike a balance between maximum compression of the data using a single cluster, and maximum accuracy by assigning each data point to its own cluster. The optimal value of *k* was determined on the basis of 4 different evaluation criteria: total within-cluster sum of squares, adjusted *R*^2^, AIC, and BIC.

#### Linear and geometric features

Linear and geometric features measure aspects of the fruit directly from images and were processed using ImageJ 2.0.0 [96, 97] and R 3.5.3 [98]. Extracted measurements included shape index (SI) [40], circularity (Circ) [97], bounding aspect ratio (BAR) [97], ellipse aspect ratio (AR) [97], roundness (Round) [97], solidity (Solid) [97], Feret aspect ratio (FAR) [97], the ratio of the height of maximum width and maximum height (HW) [40], variance (Var), skewness (Skew) [97], and kurtosis (Kurt) [97] (Fig. 4A). For Var, Skew, and Kurt, the analyses focus on the horizontal axis (Fig. 4A).

#### Elliptical Fourier analysis

EFA comprehensively described closed outlines as a series of oscillating, harmonic functions and were calculated using Momocs v1.2.9 [101] in R 3.5.3. We extracted elliptical Fourier features for the first 5 harmonics, resulting in 20 coefficients using ”Momocs::efourier()” function. Each harmonic level is made up of 4 coefficients that correspond to the effects of the cosine and sine in the *x*-axis (coefficients *A* and *B*) and the *y*-axis (coefficients *C* and *D*). To allow for discrimination between accessions based on fruit shape, principal component analysis (PCA) was performed using the ”Momocs::PCA” from Momocs for EFFs. We recorded the eigenvectors of each image on the 20 resulting principal axes (Fig. 4B).

#### Generalized Procrustes analysis and revealed latent features

GPA describes shape as the average distance between all measured landmarks on a target object and the corresponding landmarks on a reference object or centroid. The outline of each object was decomposed into 50 evenly spaced pseudo-landmarks moving clockwise around the object. The ”Momocs::fgProcrustes()” function from Momocs v1.2.9 [101] was used to perform the alignment between shapes (Fig. 4C,*left*). Each of the 50 aligned pseudo-landmarks was considered as an individual multivariate feature. Each of the 50 features was centered such that the marginal mean of both axes is 0. The ”stats::prcomp()” function in R was used to perform PCA on each of the 50 centered pseudo-landmarks (Fig. 4C, *left*and *center*).

Latent features from the calculated landmark PCs were constructed to describe the 4 most variable regions of the strawberry outline (i.e., tip, left side, neck, and right side) (Fig. 4C; *center*) with "lavaan::sem()” using the lavaan package v0.6−5 [102]. Use of SEM is commonly justified in the social sciences because of its ability to impute relationships (i.e., covariance) between unobserved constructs (latent variables) from observable variables. Here, we treated different pseudo-landmarks as observable variables to study the relationship between latent components of shape. Only those pseudo-landmarks with variance on PC1 greater than the median were used to manifest the 4 latent features (Fig. 4C, *center* and *right*). The "lavaan::predict()" function was then used to extract 5 latent variables: Tip, Side_Left_, Side_Right_, Neck, and finally Shape. Tip was manifest by a combination of PC1 of the pseudo-landmarks 1, 2, 3, 4, 5, 48, 49, and 50; Neck by PC1 of landmarks 24, 25, 26, 27, 28, and 29; Side_Left_ by PC1 of landmarks 11, 12, 13, 14, and 15; and Side_Right_ by PC1 of landmarks 38, 39, 40, 41, 42, and 43. Shape is then manifest by a combination of Tip, Neck, Side_Left_, and Side_Right_. The variances of the 5 latent variables were set to 1 for model identification. The model fit was adequate standardized root mean squared residual = 0.095, root mean square error of approximation = 0.071 ± 0.002, comparative fit index = 0.979), and Tucker-Lewis index = 0.977 [103]. However, the χ^2^ test statistic was large ($\chi ^2_{\mathrm{df}=271} = 9,724.76$;*P* < 2E−16), which likely resulted from the large sample size. We did not perform model comparisons because our goal was to quantify a reduced, latent-space representation of observed pseudo-landmarks that minimizes the difference between the model-implied and sample covariance matrices. Each of the 4 latent features was calculated for all images.

#### EigenFruit analysis

EigenFruit features were calculated from the EigenFaces and other related PCA-based methods of [58–61, 65] and incorporated information about every pixel in a given set of images. The resulting matrix of binary image vectors was 6,874 × 10,000. There could only be as many non-zero PCs as there were observations (i.e., 6,874). The ”stats::prcomp()” function was used to perform PCA. We recorded the eigenvalues of the first 20 PCs. Together these 20 PCs explained  71.7% of the variance. PC1, PC2, and PC3 explained 26.8%, 12.6%, and 5.24%, respectively (Fig. 4D, *left*).

#### Biomass profile features

Biomass profile features described the shape as the sum of pixels in each row, or column, of a given image. We adopted this method from Turner et al. [13]. We generated the horizontal biomass profile by recording the number of black pixels in each of 100 rows. The vertical biomass profile was generated by recording the number of black pixels in each of the 100 columns. The function "stats::prcomp()” in R was used to perform PCA for each profile (i.e., vertical and horizontal). The eigenvectors of the first 5 PCs from each were retained. Together these 5 PCs explained 95.9% and 95.4% of the total symmetric shape variance for the horizontal and vertical profiles, respectively (Fig. 4D, *center* and *right*).

### Broad-sense heritability estimation

#### Qualitative features

Broad-sense heritability on a clone-mean basis (*H*^2^) for each ordered level of *k* was estimated using the ordinal package v2019.3−9 [72] in R 3.5.3. Variance components were estimated using cumulative link mixed models with a cumulative logit link function and a multinomial error,

(4) $$\begin{equation*}
y_{ijk_l} = \mu + G_{i} + H_{j} + B_{k} + E_{ijk} + F_{ijk_l}
\end{equation*}$$

where $y_{ijk_l}$ is the categorical feature, μ is the grand mean, *G_i_* is the random effect of the *i*th genotype [$G_i \sim \mathcal {N}(0,\sigma ^2_{G})$], *H_j_* is the fixed effect of the *j*th harvest, *B_k_* is the fixed effect of the *k*th block, *E_ijk_* is the residual error of the *ijk*th plot [$E_{ijk} \sim \mathcal {N}(0,\sigma ^2_{E})$], and *F_ijkl_* is the error of *ijk_l_*th fruit (subsample) ($F_{ijk_l} \sim \, {\rm logit}[P(Y \le j)]_0^{k-1}$), where *k* is the number of clusters. The "clmm()” function implements cumulative link mixed models for ordinal data. Ordinal GLMMs were considered the most appropriate, and conservative, approach because we could not assume that shape categories would be linear. Variance component estimation is performed via maximum likelihood and allows for multiple random effects with crossed and nested structures [72]. *H*^2^ for each feature was calculated as

(5) $$\begin{equation*}
H^2 = \displaystyle\frac{\sigma _{G}^2}{\sigma _{G}^2 + \sigma _{E}^2/hr},
\end{equation*}$$

where $\sigma _{G}^2$ is the genetic variance, $\sigma _{E}^2$ is the residual variance, *h* is the harmonic mean of observed harvest dates per genotype (1.66), and *r* is the harmonic mean of replicates per harvest (2.50).

#### Quantitative Features

Broad-sense heritability on a clone-mean basis (*H*^2^) was estimated for features with the lme4 package v1.1−19 [74] in R 3.5.3. Restricted maximum likelihood variance components were estimated using the linear mixed effects model,

(6) $$\begin{equation*}
y_{ijk} = \mu + G_{i} + H_{j} + B_{k} + E_{ijk},
\end{equation*}$$

where *y_ijk_* is the quantitative feature, μ is the grand mean, *G_i_* is the random effect of the *i*th genotype [$G_i \sim \mathcal {N}(0,\sigma ^2_{G})$], *H_j_* is the fixed effect of the *j*th harvest, *B_k_* is the fixed effect of the *k*th block, and *E_ijk_* is the residual error of the *ijk*th plot [$E_{ijk} \sim \mathcal {N}(0,\sigma ^2_{E})$]. Only 2 harvest dates and 3 blocks were observed, and, because of this, they were treated as fixed effects. *H*^2^ for each feature was calculated as in Equation (5).

### Feature selection

Random forest regression models were fit in R 3.5.3 using the VSURF package v1.0.4 [73]. One hundred forests, each consisting of 2,000 random trees, were fit using 68 features to predict cluster assignments. The "VSURF::VSURF()” function returns 2 sets of features. The first includes important features with some redundancy, and the second, smaller set corresponds to a model focusing more closely on the classification and reducing redundancy [73]. Features that appeared in the second set for >3 levels of *k* were recorded and used for classification for all clusters (Feature Set 13). Five features that had mean OOB estimates greater than the median (OOB = 0.047) were used as Feature Set 5. Three features that had mean OOB estimates greater than the mean estimate (OOB = 0.12) were recorded as Feature Set 3.

### Classification performance

The classification accuracy was then estimated using the ”MASS::lda()” function from MASS v7.3−51.1 [104] as well the "e1071::svm()” function from e1071 v1.7−0 [105]. Classification models were trained to delineate the cluster assignments from *k*-means using the 3 different feature sets as predictor variables. All images were randomly sorted into training and test sets without stratification of size 80/20%, 50/50%, and 20/80% to explore the relationship between sample size and model performance. The training set images were clustered using the "stats::kmeans()” function in R. As before, *k* was allowed to range from 2 to 10 for this experiment. The images in the test set were assigned to the nearest cluster for each value of *k*. The PC features (i.e., EigenFruitPC[1,7], BioVPC[1,2], and BioHPC[1,3]) were calculated using only the training set images, and the test images were projected into this new space. The maximum number of non-zero PCs in this experiment for the EigenFruit analysis was either 5,500, 3,437, or 1,374, depending on the size of the training data set. The percent variance explained of each leading PC was recalculated. Geometric descriptors (i.e., BAR, SI, and Kurt) were not recalculated because they are derived from an individual sample and not a sample population. Finally, both LDA and SVR models were trained using all 3 feature sets for all values of *k* using the "MASS::lda()” and "e1071::svm()” functions in R. The trained models were used to classify the images in the respective test set. The model performance was evaluated using the average classification accuracy, precision, recall, and false-positive rate (FPR) of 10 iterations of cross-validation.

## Availability of Source Code and Requirements

 

## Availability of Supporting Data and Materials

The data supporting the results of this article and supplementary figures are available in the Zenodo repository [66]. The code to reproduce these analyses is documented and available on GitHub [67]. Data further supporting this work are available in the GigaScience repository, GigaDB [68].

## Abbreviations

AIC: Akaike information criterion; BIC: Bayesian information criterion; BLUP: best linear unbiased prediction; CSV: comma-separated values; EFA: elliptical Fourier analysis; FPR: false-positive rate; GM: Geometric Morphometrics; GPA: generalized Procrustes analysis; GWAS: genome-wide association studies; LDA: linear discriminant analysis; MDS: multi-dimensional scaling; OOB: out-of-bag error; PC: principal component; PCA: principal component analysis; PH: persistent homology; PPKC: Principal Progression of *k* Clusters; SEM: structure equation model; SIOX: Simple Interactive Object Extraction; SVR: support vector regression; VSURF: Variable Selection Using Random Forests.

## Competing Interests

The authors declare that they have no competing interests.

## Funding

This research was supported by grants to S.J.K. from the United Stated Department of Agriculture National Institute of Food and Agriculture (NIFA) Specialty Crops Research Initiative (No. 2017-51181-26833), and California Strawberry Commission (A19-3961-001), in addition to funding from the University of California, Davis. Opinions, findings, conclusions, or recommendations expressed in this publication are those of the authors and do not necessarily reflect the views of the USDA. USDA is an equal opportunity provider and employer.

## Authors' Contributions

The overall project was conceived by M.J.F. and S.J.K.; M.A.H., R.A.F., C.M.L., and G.S.C. helped grow plant material and collect raw data; M.J.F. performed the analyses; M.J.F., A.T., and S.J.K. wrote the manuscript.

## Supplementary Material

giaa030_GIGA-D-19-00292_Original_Submission

giaa030_GIGA-D-19-00292_Revision_1

giaa030_GIGA-D-19-00292_Revision_2

giaa030_GIGA-D-19-00292_Revision_3

giaa030_Response_to_Reviewer_Comments_Original_Submission

giaa030_Response_to_Reviewer_Comments_Revision_1

giaa030_Response_to_Reviewer_Comments_Revision_2

giaa030_Reviewer_1_Report_Original_Submission Michael Gore -- 9/14/2019 Reviewed

giaa030_Reviewer_2_Report_Original_Submission Mao Li -- 10/4/2019 Reviewed

giaa030_Reviewer_2_Report_Revision_1 Mao Li -- 12/5/2019 Reviewed

giaa030_Reviewer_2_Report_Revision_2 Mao Li -- 1/21/2020 Reviewed

giaa030_Reviewer_3_Report_Revision_2 Chris Armit -- 2/4/2020 Reviewed

giaa030_Supplemental_Files
