# Supplementary material for: Multi-dimensional machine learning approaches for fruit shape phenotyping in strawberry
Source: Gigascience. 2020 Apr 30;9(5):giaa030. doi: 10.1093/gigascience/giaa030 (PMC7191992; doi:10.1093/gigascience/giaa030)
Supplement: giaa030_GIGA-D-19-00292_Revision_3 [file giaa030_giga-d-19-00292_revision_3.pdf]

GigaScience

Multi-Dimensional Machine Learning Approaches for Fruit Shape Phenotyping in Strawberry

--Manuscript Draft--

Manuscript Number:	GIGA-D-19-00292R3
Full Title:	Multi-Dimensional Machine Learning Approaches for Fruit Shape Phenotyping in Strawberry
Article Type:	Research
Funding Information:	National Institute of Food and Agriculture (2017-51181-26833) — Dr Steven J Knapp<br>California Strawberry Commission (US) — Dr Steven J Knapp
Abstract:	Background: Shape is a critical element of the visual appeal of strawberry fruit and influenced by both genetic and non-genetic determinants. Current fruit phenotyping approaches for external characteristics in strawberry often rely on the human eye to make categorical assessments. However, fruit shape is inherently multi-dimensional, continuously variable trait, and not adequately described by a single categorical or quantitative feature. Morphometric approaches enable the study of complex, multi-dimensional forms but are often abstract and difficult to interpret. In this study, we developed a mathematical approach for transforming fruit shape classifications from digital images onto an ordinal scale called the Principal Progression of k Clusters (PPKC). We use these human-recognizable shape categories to select quantitative features extracted from multiple morphometric analyses that are best fit for genetic dissection and analysis.<br><br>Results: We transformed images of strawberry fruit into human-recognizable categories using unsupervised machine learning, discovered four principal shape categories, and inferred progression using PPKC. We extracted 68 quantitative features from digital images of strawberries using a suite of morphometric analyses and multi-variate statistical approaches. These analyses defined informative feature sets that effectively captured quantitative differences between shape classes. Classification accuracy ranged from 68-99% for the newly created phenotypic variables for describing a shape.<br>Conclusions: Our results demonstrated that strawberry fruit shapes could be robustly quantified, accurately classified, and empirically ordered using image analyses, machine learning, and PPKC. We generated a dictionary of quantitative traits for studying and predicting shape classes and identifying genetic factors underlying phenotypic variability for fruit shape in strawberry. The methods and approaches we applied in strawberry should apply to other fruits, vegetables, and specialty crops.
Corresponding Author:	Steven J Knapp<br><br>UNITED STATES
Corresponding Author Secondary Information:	
Corresponding Author's Institution:	
Corresponding Author's Secondary Institution:	
First Author:	Mitchell J Feldmann
First Author Secondary Information:	
Order of Authors:	Mitchell J Feldmann<br>Michael A Hardigan<br>Randi A Famula

Powered by Editorial Manager® and ProduXion Manager® from Aries Systems Corporation

	Cindy M López
	Amy Tabb
	Glenn S Cole
	Steven J Knapp
**Order of Authors Secondary Information:**	
**Response to Reviewers:**	We have added the citation to the Gigascience Database and have added statements in the sections "Data Description" and "Availability of Supporting Data and Materials" citing this repository alongside the Zenodo repository and the Github page. Because we added a similar statement citing GigaDB in the "Data Description" section, the additional citation is [68].<br><br>I have been in communications with Chris to finalize the GigaDB repository associated with this manuscript, and will approve that database as soon as it is transferred to me.
**Additional Information:**	
**Question**	**Response**
Are you submitting this manuscript to a special series or article collection?	No
**Experimental design and statistics**<br><br>Full details of the experimental design and statistical methods used should be given in the Methods section, as detailed in our Minimum Standards Reporting Checklist. Information essential to interpreting the data presented should be made available in the figure legends.<br><br>Have you included all the information requested in your manuscript?	Yes
**Resources**<br><br>A description of all resources used, including antibodies, cell lines, animals and software tools, with enough information to allow them to be uniquely identified, should be included in the Methods section. Authors are strongly encouraged to cite Research Resource Identifiers (RRIDs) for antibodies, model organisms and tools, where possible.<br><br>Have you included the information requested as detailed in our Minimum Standards Reporting Checklist?	Yes

Powered by Editorial Manager® and ProduXion Manager® from Aries Systems Corporation

**Availability of data and materials**<br><br>All datasets and code on which the conclusions of the paper rely must be either included in your submission or deposited in publicly available repositories (where available and ethically appropriate), referencing such data using a unique identifier in the references and in the "Availability of Data and Materials" section of your manuscript.<br><br>Have you have met the above requirement as detailed in our Minimum Standards Reporting Checklist?	Yes

Manuscript

Click here to access/download;Manuscript;Feldmann_2D_PPKC_19.pdf

OXFORD

(GIGA)^n SCIENCE

GigaScience, 2020, 1–24

doi: xx.xxxx/xxxx
Manuscript in Preparation
Research

RESEARCH

Multi-Dimensional Machine Learning Approaches for Fruit Shape Phenotyping in Strawberry

Mitchell J. Feldmann[1,*], Michael A. Hardigan[1], Randi A. Famula[1], Cindy M. López[1], Amy Tabb[2], Glenn S. Cole[1] and Steven J. Knapp[1,*]

[1]Department of Plant Sciences, University of California, Davis. One Shields Ave, Davis, CA 95616, USA and [2]USDA-ARS-AFRS, 2217 Wiltshire Rd, Kearneysville, WV 25430, USA

[*]mjfeldmann@ucdavis.edu; sjknapp@ucdavis.edu

Abstract

Background: Shape is a critical element of the visual appeal of strawberry fruit and influenced by both genetic and non-genetic determinants. Current fruit phenotyping approaches for external characteristics in strawberry often rely on the human eye to make categorical assessments. However, fruit shape is inherently multi-dimensional, continuously variable trait, and not adequately described by a single categorical or quantitative feature. Morphometric approaches enable the study of complex, multi-dimensional forms but are often abstract and difficult to interpret. In this study, we developed a mathematical approach for transforming fruit shape classifications from digital images onto an ordinal scale called the Principal Progression of k Clusters (PPKC). We use these human-recognizable shape categories to select quantitative features extracted from multiple morphometric analyses that are best fit for genetic dissection and analysis.
Results: We transformed images of strawberry fruit into human-recognizable categories using unsupervised machine learning, discovered four principal shape categories, and inferred progression using PPKC. We extracted 68 quantitative features from digital images of strawberries using a suite of morphometric analyses and multi-variate statistical approaches. These analyses defined informative feature sets that effectively captured quantitative differences between shape classes. Classification accuracy ranged from $68-99\%$ for the newly created phenotypic variables for describing a shape.
Conclusions: Our results demonstrated that strawberry fruit shapes could be robustly quantified, accurately classified, and empirically ordered using image analyses, machine learning, and PPKC. We generated a dictionary of quantitative traits for studying and predicting shape classes and identifying genetic factors underlying phenotypic variability for fruit shape in strawberry. The methods and approaches we applied in strawberry should apply to other fruits, vegetables, and specialty crops.

Key words: *Fragaria*×*ananassa*; Fruit shape; Morphometrics; Latent Space Phenotypes; Machine learning; Principal Progression of k Clusters

Background

Fruit breeders actively selected several morphological and quality phenotypes during the domestication of garden strawberry (*Fragaria* × *ananassa*), an allo-octoploid (2n = 8x = 56) of hybrid origin [1–3]. *F.* × *ananassa* was created in the early 1700s by interspecific hybridization between ecotypes of wild octoploid species (*F. virginiana* and *F. chiloensis*), multiple subsequent introgressions of genetic diversity from *F. virginiana* and *F. chiloensis* subspecies in subsequent generations, and artificial selection for horticulturally important traits among interspecific hybrid descendants. Domestication and breeding have altered the fruit morphology, development, and metabolome of garden strawberry, distancing modern cultivars from their wild progenitors [4–9]. Approximately 300 years of breeding in the admixed hybrid population has led to the emergence of high yielding cultivars with large, firm, visually appealing, long shelf-life fruit that can withstand the rigors of harvest, handling, storage, and long-distance shipping [10]. Fruit shape is an essential trait of agricultural products, particularly those of specialty crops, due to perceived and realized relationships with the quality and value of the products. Image-based fruit phenotyping has the potential to increase scope, throughput, and accuracy in quantitative genetic studies by reducing the effects of user bias, enabling the analysis of larger sample sizes, and more accurate partitioning of genetic variance from environments (E), management (M), and other non-genetic sources of variation [11–13].

Many fruit phenotyping approaches rely on the human eye to sort fruit into discrete, descriptive categories for planar (2D) shapes (e.g., rhombic and

Compiled on: February 7, 2020.
Draft manuscript prepared by the author.

reniform)[14–19]. Categories are either nominal [11, 20, 21], existing in name only, or ordinal, referring to a position in an ordered series or on a gradient [15, 16, 21]. Classification into categories is often labor-intensive and prone to human bias, which can increase with task complexity and time requirements [22, 23]. Alternative scoring approaches rely on morphometrics and machine learning to automate classification; for example, sorting fruit into shape categories in both tomato [11] and strawberry [20]. Unsupervised machine learning methods (e.g., k-means clustering), unlike supervised methods, are useful for pattern detection and clustering, while supervised machine learning methods (e.g., support vector machines) are useful for prediction and classification [24, 25]. Unsupervised clustering enables the calculation of several measures of model performance and overfitting to balance compression and accuracy. However, the categories derived from these techniques are without order, resulting in the need for a suitable transformation to an ordinal scale more appropriate for quantitative genetic analyses [26–30]. In this context, ordinal categories give the interpretation of relationship with, or distance from, other shape categories in a series. To enable this interpretation, we developed a method for asserting the progression through fruit shape categories derived from unsupervised machine learning methods. The Principal Progression of k Clusters (PPKC), allowed us to non-arbitrarily determine the appropriate shape gradient for statistical analyses using empirical data. The advantages of PPKC, relative to a manually-determined ordinal scale, are that it does not require arbitrary, *a priori* decisions and is unsupervised, which avoids additional operator bias. Here, we describe approaches for translating digital images of strawberries into computationally defined phenotypic variables for identifying and classifying fruit shapes.

Fruit shape and anatomy are complex, multi-dimensional, and, potentially, abstract phenotypes that are often not completely or intuitively described by planar descriptors and individual qualitative or quantitative variables. Beyond the qualitative definitions used in plant systematics [18, 20], references to fruit shape encompass a wide variety of mathematical parameters and geometric indices that establish quantitative measurements of plant organs [19, 31–33]. Much like human faces or grain yield, fruit shape and anatomy are products of the underlying genetic and non-genetic determinants of phenotypic variability in a population [34, 35]. Quantitative phenotypic measurements have allowed researchers to uncover some of the genetic basis of fruit shape in tomato [36, 37], pepper [38, 39], pear [40], melon [35], potato [41], and strawberry [9, 42]. However, the major genetic determinants of fruit shape remain unclear, or understudied, in octoploid strawberry, in part because researchers have not yet translated fruit shape attributes into holistic, quantitative variables, which may empower the identification of underlying genes or quantitative trait loci through genome-wide association studies (GWAS) and other quantitative genetic approaches [43–46]. Quantitative features often rely on linear metrics of distance (e.g., height, width, and perimeter) and are generally modified into compound descriptors that remove the effects of size (e.g., aspect ratio or roundness) [40, 42, 47]. However, compound linear descriptors often have limited resolution compared to more comprehensive, multi-variate descriptors [33]. Elliptical Fourier Analysis (EFA) quantifies fruit shape from a closed outline by converting a closed-contour into a weighted sum of harmonic functions [12, 48–51]. Generalized Procrustes Analysis (GPA) quantifies the distance between sets of biologically homologous, or mathematically similar, landmarks on the surface of an object [48, 51–57]. Fruit shape can also be described using linear combinations of pixel intensities from digital images extrapolating from analyses generally used to quantify color patterns and facial recognition [13, 58–63]. Similar pixel-based descriptors have recently been referred to as "Latent Space Phenotypes" (LSP) and arise from unsupervised analyses (i.e., principal components analysis and auto-encoding neural networks) that allow a computer to produce novel, independently distributed features directly from images [64, 65]. Here, we generate a dictionary of 68 quantitative features, including linear-, outline-, landmark-, and pixel-based descriptors to investigate the quality of different features in preparation for quantitative genetic analyses.

The ultimate goal of our study was to develop heritable phenotypic variables for describing fruit shape, which could then be used to identify the genetic factors underlying phenotypic differences in fruit shape. The phenotyping and analytic work flow for this study are summarized in Figures 1 and 2. We first describe and demonstrate the application of PPKC, which transforms categories discovered from unsupervised machine learning methods to a more convenient and analytically tractable ordinal scale [26, 28, 29]. We then explore the relationship between machine-acquired categories and 68 quantitative features extracted from digital images. Next, we apply random forest regression to select critical sets of quantitative features for classification and use supervised machine learning methods, including support vector regression and linear discriminant analysis, to determine the accuracy of shape classification. We discovered that there are only a few categories of interest in a highly domesticated breeding population and that a small number of features are needed to classify shape into the discovered categories accurately. We also find that ordinal shape categories are highly heritable and that the features needed for accurate classification are also heritable.

Data Description

The data released with this manuscript contains digital images of $6,874$ strawberry fruit from 572 hybrids originating from the University of California, Davis Strawberry Breeding Program. The data for this manuscript, including pre-processed images (Fig. 1A), processed images (Fig. 1B), and extracted features (see Methods; Fig. 2), are available on Zenodo [66]. The pre-processed images typically contained multiple berries per image along with a data matrix bar code indicating the genotype ID and other elements of the experiment design. The processed images are 1000×1000px-scaled binary images of individual fruit. The extracted features data set is provided as a CSV file. The code to replicate the analyses in this manuscript is provided in a GitHub repository [67]. Additionally, snapshots of the code and data supporting this work are available in the GigaScience repository, GigaDB [68]. We hope that the release of this data assists others in developing novel morphometric approaches to better understand the genetic, developmental, and environmental control of fruit shape in strawberry, and more broadly in other fruits, vegetables, and specialty crops.

Analyses

k-means clustering

k-means clustering rapidly detects patterns in large, multi-dimensional data sets used for clustering, decision making, and dimension reduction [24, 69, 70]. It is an iterative algorithm that partitions a data set into a pre-defined number of non-overlapping clusters, k, by minimizing the sum of squared distances from each data point to the cluster centroid. A centroid corresponds to the mean of all points assigned to the cluster. Here, we used k-means to cluster flattened binary images (Fig. 1; see Methods). Individual fruits were segmented from the image background as a binary mask, normalized by the major axis, resized to 100×100px, and flattened into a vector (Figs. 1 and 2; see Methods). We represented each image as a $10,000$ element vector containing binary pixel values. We were able to rapidly and reliably assign images to classes using k-means clustering. In this experiment, we allowed k, the number of permitted categories, to range from 2 to 10. This range was chosen because we anticipate that a human-based classification system would not have the speed or reliability needed for this task, particularly for larger values of k.

Principal progression of k clusters

k-means clustering does not assign a progression or gradient to discovered classes. However, score and ordinal traits are typically more useful and are more common in quantitative genetic studies than nominal scales [26, 28, 29, 71]. We developed a new method to transform the categories derived from k-means onto an ordinal scale, which we call the principal progression of k clusters, or PPKC (Fig. 3; Alg. 1). This method relies on k-means clustering to categorize images and can be used to discover an appropriate ordinal scale in nominal data empirically. k-means supports several metrics for evaluating model performance and overfitting, including adjusted R^2, Akaike Information Criterion (AIC), and Bayesian Information Criterion (BIC), which allows users to determine the most appropriate value of k given the observed data. The gradient between clusters was estimated by performing principal components analysis on a covariance matrix reflecting the structured relationship between a focal cluster and all previously discovered clusters.

We first assign each flattened binary image (Fig. 1) to a category using a k-means approach. We assign a cluster to each image and allow the number of clusters, k, to range from $[2, 10]$. The order is subsequently inferred using PPKC (Fig. 3; Alg. 1). When $k = 2$, the order of relatedness is considered arbitrary, and both $k2_{c1} \rightarrow k2_{c2}$ and $k2_{c2} \rightarrow k2_{c1}$ have the same meaning, where "$\longrightarrow$" indicates the progression of discovered categories. Any given order and its reverse are considered equivalent, and this applies to higher levels of k as well; for example, the hypothetical ranking of clusters $1, 4, 2, 3$ is considered equivalent to $3, 2, 4, 1$ as the relative relationship between the k clusters is identical in both (e.g., $c3$ is more related to $c2$ than either $c1$ or $c4$). For each cluster of interest (e.g., $k4_{c1}$, $k4_{c2}$, $k4_{c3}$, and $k4_{c4}$), we calculate the proportion of each cluster that came from $k3_{c1}$, $k3_{c2}$, or $k3_{c3}$ and $k2_{c1}$ or $k2_{c2}$ (i.e., all former classifications). These proportions enable the estimation of similarity between a focal cluster (e.g., $k4_{c1}$) and the clusters of all prior values of k. We then normalize the proportions by the total number of images in the focal cluster (e.g., $k4_{c1}$, $k4_{c2}$, $k4_{c3}$, and $k4_{c4}$) (Eqn. 1).

For every level of $k > 2$, we construct $\mathbf{M}$, a rectangular matrix of size $\frac{k^2-k}{2} - 1 \times k$ (Alg. 1 line 13). The sum of each column should equal $k - 2$. The proportions are continuous values in the range $[0, 1]$ that described the origin of a particular focal cluster (e.g., $k4_{c1}$) as it relates to the clusters of $k = 3$ and $k = 2$ or all clusters $[2, k - 1]$. In the following example, $k = 4$:

$$\mathbf{M} = \begin{bmatrix} \frac{|k4_{c1} \wedge k3_{c1}|}{|k4_{c1}|} & \frac{|k4_{c2} \wedge k3_{c1}|}{|k4_{c2}|} & \frac{|k4_{c3} \wedge k3_{c1}|}{|k4_{c3}|} & \frac{|k4_{c4} \wedge k3_{c1}|}{|k4_{c4}|} \\ \frac{|k4_{c1} \wedge k3_{c2}|}{|k4_{c1}|} & \frac{|k4_{c2} \wedge k3_{c2}|}{|k4_{c2}|} & \frac{|k4_{c3} \wedge k3_{c2}|}{|k4_{c3}|} & \frac{|k4_{c4} \wedge k3_{c2}|}{|k4_{c4}|} \\ \frac{|k4_{c1} \wedge k3_{c3}|}{|k4_{c1}|} & \frac{|k4_{c2} \wedge k3_{c3}|}{|k4_{c2}|} & \frac{|k4_{c3} \wedge k3_{c3}|}{|k4_{c3}|} & \frac{|k4_{c4} \wedge k3_{c3}|}{|k4_{c4}|} \\ \frac{|k4_{c1} \wedge k2_{c1}|}{|k4_{c1}|} & \frac{|k4_{c2} \wedge k2_{c1}|}{|k4_{c2}|} & \frac{|k4_{c3} \wedge k2_{c1}|}{|k4_{c3}|} & \frac{|k4_{c4} \wedge k2_{c1}|}{|k4_{c4}|} \\ \frac{|k4_{c1} \wedge k2_{c2}|}{|k4_{c1}|} & \frac{|k4_{c2} \wedge k2_{c2}|}{|k4_{c2}|} & \frac{|k4_{c3} \wedge k2_{c2}|}{|k4_{c3}|} & \frac{|k4_{c4} \wedge k2_{c2}|}{|k4_{c4}|} \end{bmatrix} \quad (1)$$

We then calculatee the variance-covariance matrix of Eqn. (1) (Alg. 1; line 18). The variance-covariance matrix, $\Sigma_{\mathbf{M}}$, represents the relationship between each focal cluster (e.g., $k4_{c1}$, $k4_{c2}$, $k4_{c3}$, or $k4_{c4}$).

$$\Sigma_{\mathbf{M}} = \begin{bmatrix} \sigma^2_{k4_{c1}} & \sigma_{k4_{c2},k4_{c1}} & \sigma_{k4_{c3},k4_{c1}} & \sigma_{k4_{c4},k4_{c1}} \\ \sigma_{k4_{c1},k4_{c2}} & \sigma^2_{k4_{c2}} & \sigma_{k4_{c3},k4_{c2}} & \sigma_{k4_{c4},k4_{c2}} \\ \sigma_{k4_{c1},k4_{c3}} & \sigma_{k4_{c2},k4_{c3}} & \sigma^2_{k4_{c3}} & \sigma_{k4_{c4},k4_{c3}} \\ \sigma_{k4_{c1},k4_{c4}} & \sigma_{k4_{c2},k4_{c4}} & \sigma_{k4_{c3},k4_{c4}} & \sigma^2_{k4_{c4}} \end{bmatrix} \quad (2)$$

We then perform eigen decomposition on Eqn. (2) using the following equation (Alg. 1; line 19).

$$\Sigma_{\mathbf{M}} = \mathbf{V} \Lambda \mathbf{V}^{-1} \quad (3)$$

In Eqn. (3), Λ is a diagonal matrix with values corresponding to the k eigenvalues of $\Sigma_{\mathbf{M}}$ and $\mathbf{V}$ is a square matrix containing eigenvectors associated with the eigenvalues in Λ. We then extract the eigenvector associated with the largest eigenvalue, $\vec{v}_{\lambda_{max}}$. We order the elements of $\vec{v}_{\lambda_{max}}$ such that the resultant vector, $\vec{v_s}$, has the property $v_{s_1} \leq \ldots \leq v_{s_k}$. We do not consider the distance between elements in $\vec{v_s}$, only their rank. The clusters are then indexed to match the rank of the associated elements in $\vec{v_s}$. There are at most k eigenvalues associated with eigenvectors of length k due to $\Sigma_{\mathbf{M}}$ being $k \times k$. Eigen decomposition is used to describe the major axis of variance in $\Sigma_{\mathbf{M}}$. In theory, this perspective of covariance should be able to separate the classes effectively as it describes a linear axis containing the greatest amount of independent variation and solutions are non-arbitrary. The value a category takes on this composite axis is therefore suggestive of its linear relationship to other the k categories being considered. However, we note that relationships containing branches, bubbles, and other topological features will not be captured accurately. In this study, we are unable to report a visually meaningful order when $k \geq 9$ (Fig. S1). The change in progression could be reflective of overfitting the number of groups in k-means clustering. The large change of slope at $k = 4$ in the total within-group sums of squares, AIC, and Adjusted R^2 evidenced overfitting (Fig. S2). The strongest evidence for four clusters is in the BIC, which is minimized when $k = 4$ (Fig. S2D). The elements of $\vec{v_s}$ tend to converge on one another as k increases, which may be indicative of little biological information in the new clusters and overfitting (Fig. S3). Given that only relatively small covariance matrices are considered in this algorithm, the computational time to order $k = [3, \ldots, 10]$ on an early 2015 MacBook Pro 2.9 GHz Core i5 with 8GB memory is < 0.2 seconds.

Algorithm 1 Principal Progression of K Clusters (PPKC) Algorithm

1: $k = 10$
2: **for** $i = 2$ to k **do**
3: Compute class assignments for i using k-means clustering.
4: ▷ Only needs to be done once.
5: **end for**
6: **for** $j = 3$ to k **do**
7: $\vec{x}$ = assignment to j classes
8: **for** $a = 1$ to j **do**
9: $r = 1$
10: **for** $b = 2$ to $j - 1$ **do**
11: $\vec{y}$ = assignment to b classes
12: **for** $d = 1$ to b **do**
13: $\mathbf{M}_{r,j} = \frac{|a \in \vec{x} \wedge d \in \vec{y}|}{|a \in \vec{x}|}$
14: $r{+}{+}$
15: **end for**
16: **end for**
17: **end for**
18: $\Sigma_{\mathbf{M}} = Cov(\mathbf{M})$ ▷ Variance-covariance of $\mathbf{M}$
19: $\Sigma_{\mathbf{M}} = \mathbf{V} \Lambda \mathbf{V}^{-1}$ ▷ Eigen decomposition of $\Sigma_{\mathbf{M}}$
20: $\Lambda = \lambda_{max}, \ldots, \lambda_k \mathbf{I}$ ▷ λ_{max} is the largest eigenvalue of $\Sigma_{\mathbf{M}}$.
21: $\vec{v}_{\lambda_{max}} = \mathbf{V}_{.,1}$ ▷ $\vec{v}_{\lambda_{max}}$ is the eigenvector of λ_{max}.
22: Order elements of $\vec{v}_{\lambda_{max}}$ such that the resulting vector, $\vec{v_s}$, has the property $\vec{v}_{s1} \leq \ldots \leq \vec{v}_{sk}$
23: The order of elements in $\vec{v}_s$ is the sorted order for the clusters at k.
24: Re-index clusters according to their rank in $\vec{v}_s$.
25: **end for**

Broad-sense heritability of ordered categories

For each value of k, broad-sense heritability (H^2) on an entry-mean basis was assessed using a general linear mixed model with a cumulative logit link function (see Methods; Eqn. 4 and Eqn. 5) [72]. For this data set, H^2 was generally high, ranging from $H^2 = 0.80$ to 0.98, even as $k \longrightarrow 10$ (Table 2). These estimates of H^2 are very similar to those reported in [16] (i.e., $H^2 = 0.84$). When the H^2 of a trait is in this range, it indicates that independent replications of the same individuals share a high degree of similarity and that most of the variation among individuals originated from genetic variation among individuals. Since the plant material used in this study are genetic clones, any variation in fruit shape among replicates originated from random, unobserved effects. For $k \geq 9$, the accuracy of H^2 estimates is expected to be lower than for $k \leq 8$ as the gradient of the phenotype seems to be improperly specified. In this set of germplasm, we propose a set of four primary classes for categorizing fruit shape (Fig. 3 and S2). As k increases from 5 to 10, the visual similarity of some clusters is high (Fig. S1), thus indicating fewer relevant delineations (Fig. S3). As indicated, there is strong evidence in this data that there are four distinct clusters in this data (Fig S2).

Feature selection using random forests

To discover which of 68 quantitative features (summarized in Figures 4 and 5) capture and reflect differences in shape categories, supervised machine learning was employed to estimate feature importance (see Methods) [73]. Of the 68 features used as predictors in a random forest regression (see Methods), we selected only 13. Out-of-Bag (OOB) error is an estimate of how poorly models perform when a specific feature is excluded and is akin to error estimated from jackknife re-sampling (Fig 6). In this way, features with higher estimates tend to be more relevant for classification and prediction. In this experiment, fea-

tures could only be selected up nine times, once per value of k. We maintained features that were selected in ≥ 3 levels of k to use as independent variables in classification (Table 1). The 13 selected features accounted for $> 80\%$ of importance assigned to the 68 features across all values of k (Fig 6B). Here, the use of "EigenFaces," an analysis from the 1980s, designed to classify human faces, was re-purposed for the quantification and classification of fruit shape in strawberry [58–61]. Pixel-based features dominated the selected features and include PCs $1-7$ of the EigenFruit analysis (EigenFruitPC$_{[1,6]}$), PCs 1 and 2 of the vertical biomass profile (BioVPC$_{[1,2]}$), and PCs 1 and 2 of the horizontal biomass profile (BioHPC$_{[1,3]}$) (Table 1; Fig. 6 and 7). These features originated from the same data type as used in k-means clustering (i.e., pixel intensities), which is likely the reason they make up the majority of the selected features (Table 1; Fig. 6 and 7). Several geometric descriptors were also selected, including the bounding aspect ratio (BAR), Shape Index (SI), and Ellipse Aspect Ratio (AR) (Table 1; Fig. 6 and 7). We generated a subset of five features with mean $OOB \geq 0.047$ (Fig. 6A). $OOB = 0.047$ was the median OOB error for all features across all classes. This subset of features included EigenFruitPC$_{[1,2]}$, BioVPC$_1$, and BioHPC$_{[1]}$ (Table 1). We also generated a third smaller set that included only EigenFruitPC$_1$, BioVPC$_1$, and BioHPC$_1$ with mean $OOB \geq 0.12$ (Fig. 6A). $OOB = 0.12$ was the mean OOB error for all features across all classes. The prevalence of pixel-based descriptors in these selected subsets indicated the magnitude of relevant shape information that they described.

Broad-sense heritability and relationship of selected features

While the continuous nature of the morphometric features is expected to be more conducive and provide higher resolution to quantitative genetic analyses compared to their categorical counterparts, it is also vital that these features be heritable. The H^2 for each feature was estimated on a clone-mean basis using a linear mixed-effect model (see Methods; Eqn. 6 and Eqn. 5) [74]. The H^2 for each feature is reported in Table 1. Estimates of H^2 for the quantitative features ranged from low (> 0.3) to high (> 0.7). Heritability estimates were consistent with those previously reported for shape phenotypes in strawberry and other plant species [12, 42, 75].

Figure 7A shows the directions of the feature variance-covariance matrix with the traits labeled as in Figure 6. Figure 7B shows the correlation matrix between the 13 selected features. For the five features selected by OOB error (Fig. 6), indicated with a 5 in Table 1, the estimated H^2 was ≥ 0.58. As the majority of selected features are principal components of different pixel-based analyses (Fig. S5), there were many weak correlations (Fig. 7B). We hypothesize that the importance of these features is partly driven by the similarity of the raw data (i.e., binary pixel intensities) used in k-means clustering to acquire shape categories and for EigenFruit shape analysis. Although principal components are uncorrelated, we observed strong correlations between PCs from different analyses (Fig. 7). EigenFruitPC$_1$ shared a strong positive correlation with both BioVPC$_1$ and BioHPC$_1$ ($\rho = 0.98; p < 2e-16$ and $\rho = 0.93; p < 2e-16$, respectively), as did EigenFruitPC$_2$ with BioVPC$_2$ ($\rho = 0.86; p < 2e-16$). BioHPC$_2$ was negatively correlated with both EigenFruitPC$_2$ and BioVPC$_2$ ($\rho = -0.92; p < 2e-16$ and $\rho = -0.81; p < 2e-16$, respectively). BioHPC$_3$ was negatively correlated with EigenFruitPC$_4$ ($\rho = -0.87; p < 2e-16$). BAR was negatively correlated with EigenFruitPC$_1$, BioVPC$_1$ and BioHPC$_1$ ($\rho = -0.89; p < 2e-16$, $\rho = -0.87; p < 2e-16$, and $\rho = -0.78; p < 2e-16$, respectively). Reported p-values were Bonferroni adjusted for all pairwise tests between the 13 selected features (e.g., 78).The correlations between these features indicated that the pixel-based descriptors describe comparable patterns of phenotypic variation.

Image Classification using Selected Features

The accuracy of classification, or prediction, is typically assessed by cross-validation [24, 76]. We generated training sets that consisted of 80% ($5,500$), 50% ($3,437$), or 20% ($1,374$) of the images. Assignment to either training or test set was random and without stratification. It is possible that stratification would be needed for more iterations, > 10, smaller sample sizes, or very unequal images per k category. k-means clustering was performed using the training sets, and k was allowed to range from 2 to 10. We assigned the test set images to the nearest neighboring cluster for each level of k. We performed PPKC on the clusters derived from the training set and the similarity between the full set and training sets were visually assessed. The clusters derived from the different sets appeared to be nearly identical (Fig. S6). The order of clusters derived from the reduced data set also appears identical to those described in the full set (Fig. S6). The principal component-based features were recalculated using the training data sets and the corresponding test set images projected into the new space. We only extracted the 13 selected features. These included EigenFruitPC$_{[1,6]}$, BioVPC$_{[1,2]}$, and BioHPC$_{[1,2]}$ (Table 1). The selected geometric features, including BAR, SI, and AR, were not recalculated as they do not change concerning the other samples, unlike k-means and PCA which both rely on and change based on observed data. For EigenFruitPC$_{[1,6]}$, BioVPC$_{[1,2]}$, and BioHPC$_{[1,2]}$, the percent variance explained by each feature was similar to that in full data set (Table 1), indicating that the principal components derived from the reduced set describe similar features of shape as those derived from the full set.

Support vector regression (SVR) and linear discriminant analysis (LDA) were both used for classification (see Methods). We performed ten iterations of each set size and feature set across all levels of k. The results of this experiment are reported in Table 2. Overall, the models performed with high accuracy of classification. Generally, as we used fewer features for classification model performance is reduced, most notably for larger values of k. Indeed, when $k = 2$ accuracy improved slightly with fewer features in the different models. In general, SVR was found to outperform linear discriminant analysis consistently. LDA only outperformed SVR with very small train sets relative to the test set 2. Using five features for classification, we achieve the highest accuracy (99.5%) for $k = 2$. In the range of interest, $k = [2, 4]$, the models do not fall below 90.0% accuracy for any training set size.

Discussion

As high-throughput phenotyping for external fruit characteristics becomes of interest to specialty crop researchers, we expect that this work will have various applications in both applied and basic plant research [12, 13, 51, 64, 65], intellectual property protection and documentation [77, 78], and waste reduction [20, 79]. Our study showed that strawberry fruit shapes could be robustly quantified and accurately classified from digital images. Most importantly, our analyses yielded quantitative phenotypic variables that describe fruit shape (Fig. 4), arise from continuous distributions, and are moderately to highly heritable (Table 1). We accomplished this by translating two-dimensional, digital images of fruit into categorical and continuous phenotypic variables using unsupervised machine learning and morphometrics. We found that mathematical approaches developed for human-face recognition [58, 59] were powerful for strawberry fruit shape phenotyping (Table 1), that unsupervised shape clustering was robust to sample size deviations (Fig. S6), and that only a few quantitative features are needed to accurately classify shapes from images (Table 2) indicating a paradigm appropriate for genetic dissection.

Digital plant phenotyping is able to empower quantitative genetic analyses by providing heritable and biologically relevant, latent phenotypes in a cost-effective manner [13, 64, 65, 80, 81]. In many cases, these latent traits are derived from principal components analysis (PCA), multi-dimensional scaling (MDS), structured equation modeling (SEM), persistent homology (PH), or auto-encoding convolutional neural networks which can be exceedingly abstract and difficult to interpret biologically, but may also reveal unexpected patterns of phenotypic and genetic variation [12, 13, 19, 24, 51, 59, 61, 75, 82–84]. Many of the features described in this study along with those reported in Turner et al. [13] (i.e., biomass profile), Migicovsky et al. [12] (i.e., elliptical Fourier PCs and persistent homology PCs), and Gage et al. [65] (i.e., image PCs and convolutional encodings), had high heritability (Table 1) and are exciting targets for future quantitative genetic analyses, including GWAS and genomic prediction, which were have been shown to be successful for shape features in recent work in rice (*Oryza sativa* L.) [85], apple (*Malus domestica*) [86], and pear (*Pyrus* spp.) [87]. However, the H^2 of one selected feature in this study, EigenFruitPC$_3$, was estimated to be 0.00 (Table 1 and Fig. S4). Similar results were reported in carrot (*Daucus carota* L.) for pixel-based root and shoot features [13], apple (*Malus domestica*) for elliptical Fourier leaf shape features

[12], and corn (*Zea mays*) for pixel-based shoot features [65]. Turner et al. [13] attributed the null H^2 of root shape characteristics to low phenotypic variation between the inbred parents and genotype $\times$ environment interactions. This pattern, while seemingly present, was not discussed in detail in either Migicovsky et al. [12] or Gage et al. [65]. While there may be many drivers for this pattern, we hypothesize that the null estimate may arise from the pixel-based descriptors describing more complex aspects of fruit or root shape. If the non-genetic component of a multi-variate phenotype is large, then performing PCA on that multi-variate trait could produce leading principal components that describe mostly non-genetic variance (e.g., environment, management, and residual). However, there are too few reports to adequately determine the likelihood and causal source of this phenomenon.

We empirically derived the shape progression produced in the present study through the application of a new method, PPKC, and used these mathematical categories to interpret the extracted shape features (Alg. 1; Fig. 3). Ordinal categorical traits are commonplace in quantitative genetic studies [29, 71], a current standard for phenotyping external fruit characteristics [14, 15, 42], and enable understanding and explanation of complex, latent space plant phenotypes (Figs. 6 and 7). PPKC specifically considers the relationship between a cluster at k and all clusters for values $< k$ as a covariance matrix and projects this k-dimensional space to 1-D using eigen decomposition. Ordination using dimension reduction techniques, including PCA, correspondence analysis (CA), and MDS has been previously proposed and used in community ecology [88]. Theoretically, the eigen decomposition step of PPKC could be replaced with another technique. However, unlike methods using eigen decomposition, which progressively subdivides variation such that the position on the leading axis (i.e., PC1) is fixed regardless of the number of axes examined, the position of samples on MDS axes may change when different dimensions are extracted, making MDS axes arbitrary and without meaning other than a convenient reference [88]. PPKC identified four exemplary strawberry shape categories in the population we studied, which were characterized by a progression from 'longer-than-wide' (prolate) to 'wider-than-long' (oblate) (Figs. 3 and S7). This ordinal scale can be used in breeding and research programs as traits of interest, or they can be used to organize and interpret more abstract quantitative features, such as EigenFruitPCs or SEM latent variables, through supervised machine learning algorithms [24]. Critically, this gradient agreed with the arbitrarily defined progressions in previous reports [14, 16]. However, unlike previous studies, which suggested using nine ordinal [14] or eleven nominal shape categories [20], our work presented empirical evidence for a smaller number of mathematically defined shape categories. We determined that $k = 4$ was the appropriate level of complexity based on the visual appearance of the discovered clusters (Fig. 3), high H^2 estimates (Table 2), and the information criteria calculated for the k-means models (Fig. S2). Interestingly, PPKC can determine a visually, reasonable phenotypic gradient up to $k = 8$ (Fig. S3) despite strong evidence of overfitting for $k > 4$ (Fig. S2). We extrapolate that PPKC should continue to work beyond $k = 9$ so long as new clusters are distinct and do not arise as an artifact of overfitting k.

The specific genetic factors that give rise to variation in fruit shape in octoploid, garden strawberry are currently unclear or understudied. The selective pressure exerted on fruit shape in strawberry could have impacted large-effect loci, in which case ordinal phenotypic scores are likely to be sufficient for identifying genetic factors affecting fruit shape. Loss- and gain-of-function mutations have played an essential role in identifying genes affecting fruit shape in tomato, a model that has been highly instructive and important for understanding the genetics of fruit shape and enlargement in plants [34–36, 89, 90]. There are striking examples in tomato and other plants where identified genes regulate the development of fruit shape. For example, the *OVATE* gene in tomato regulates the phenotypic transition from round- to pear-shaped fruit [91, 92]. If large-effect mutations underlie differences in strawberry fruit shape, the ordinal classification system proposed here should enable the discovery of such effects. Furthermore, quantitative phenotypes were linked to genetic features that interact with large-effect genes, i.e., suppressors of *OVATE (sov)*, through bulk segregant analysis and QTL mapping [93]. In woodland strawberry (*F. vesca*), fruit size and shape are linked to the accumulation and complex interaction of auxin, GA, and ABA, mediated by the expression and activity of *FveCYP707* and *FveNCED*, as well as other genes [9]. Because of the high H^2 estimates for several of the newly created phenotypic variables (Table 1), we hypothesize that quantitative, latent space phenotypes can yield a more comprehensive understanding of the underlying genetic mechanisms of fruit shape in garden strawberry through genome-wide association studies and other quantitative genetic analyses [44, 45, 94]. We anticipate that the analyses in this study will enable us to discover and study the genetic determinants of fruit shape in strawberry and other specialty crops.

Methods

Mating and Field Design

Seventy-five bi-parental crosses were generated by controlled pollination of 30 parents in an incomplete (16×14) factorial mating design. These parents were chosen to represent a broad range of phenotypic diversity in the University of California, Davis strawberry germplasm. $2,800$ hybrid progeny were planted at the Wolfskill Experimental Orchard in Winters, CA in sets of 20 or 40 per family, depending on seedling survival. 20% of the planted materials from each family were randomly selected for further testing. Clones of 545 of the selected 560 progeny were successfully propagated. 12 bare-root runner plants of each of the 545 progeny and the 30 parents were collected and planted in November 2017 in Salinas, CA in 4 plant plots as a randomized complete block design with three replicates of each genotype.

Image acquisition

Strawberries were harvested from plots in Salinas, CA once in April 2018 and again in May 2018. Digital images of up to 3 fruit per plot were imaged using a Sony α-6000 Mirrorless digital camera mounted on a portable copy stand in aperture priority, with the aperture set to f/8. Strawberries with the calyx removed were placed in the frame against a black felt backdrop, along with a QR-code identifying the plot, such that the most extensive face was perpendicular to the sensor. Berries were mounted to set of staples to eliminate any rolling or pitch of the berries. The time to stage a given set of fruit and acquire an image ranged from 1 to 2 min. All images were acquired with a $16-50$ mm lens set to 16 mm and positioned approximately 16 cm above the base of the copy stand resulting in images with 97.4 pixels per cm. In total, $2,924$ plots were imaged over the two harvest dates.

Image Processing

Input files were JPEG images (3008px$\times$$1688$px) with the strawberries placed in regular positions within a scene. All images were first segmented and converted to binary using the Simple Interactive Object Extraction (SIOX) tool in ImageJ $2.0.0$ [95–97] through custom batch scripts. Images that were unsuccessfully segmented were flagged and handled individually to ensure completeness. ImageJ was used to acquire the bounding rectangle of each object of interest. Each object was extracted based on the dimensions of its bounding rectangle using R $3.5.3$ [98] and the jpeg package [99]. White pixels were added to the edges of each image such that the resulting images is a square of size $\max(H, W) \times \max(H, W)$ using the "magick::image_border()" package [100]. "magick::image_resize()" was used to scale the images from $\max(H, W) \times \max(H, W)$ px to 1000×1000 px. This method results in binary images that maintain the original aspect ratio with a maximum dimension equal to $1,000$ pixels and then resized to 100×100.(Fig. 1). In total, the downstream analyses included $6,874$ images of individual berries.

Feature extraction

Categorical features

This method afforded clustering decisions based on raw image data instead of the extracted quantitative features. Each image matrix was flattened into a single $10,000$ element row vector; all of the samples were then bound together by columns. The resulting matrix for all samples was $6,874 \times 10,000$. The "stats::kmeans()" function in R was used to perform k-means clustering. Values of k (i.e., the number of clusters) range from 2 to 10. Assigned clusters were recorded for all values of k. Discovered clusters are then ordered using

PPKC (Fig. 3). The ordered categories, across the various levels of k, became the response for classification experiments. The correct choice of k is often ambiguous, with interpretations depending on the shape and scale of the distribution of points in a data set and the desired clustering resolution of the user. In addition, increasing k without penalty will always reduce the amount of error in the resulting clustering, to the extreme case of zero error if each data point is considered its own cluster (i.e., when k equals the number of data points, n). Intuitively then, the optimal choice of k will strike a balance between maximum compression of the data using a single cluster, and maximum accuracy by assigning each data point to its own cluster. The optimal value of k was determined based on four different evaluation criteria: total within-cluster sum of squares, adjusted R^2, AIC, and BIC.

Linear and geometric features

Linear and geometric features measure aspects of the fruit directly from images and were processed using ImageJ $2.0.0$ [96, 97] and R $3.5.3$ [98]. Extracted measurements included Shape Index (SI) [40], Circularity (Circ) [97], Bounding Aspect Ratio (BAR) [97], Ellipse Aspect Ratio (AR) [97], Roundness (Round) [97], Solidity (Solid) [97], Feret Aspect Ratio (FAR) [97], the ratio of the height of max width and max height (HW) [40], Variance (Var), Skewness (Skew) [97], and Kurtosis (Kurt) [97] (Fig. 4A). For Var, Skew, and Kurt, the analyses focus on the horizontal axis (Fig. 4A).

Elliptical Fourier analysis

EFA comprehensively described closed outlines as a series of oscillating, harmonic functions and were calculated using Momocs v$1.2.9$ [101] in R $3.5.3$. We extracted elliptical Fourier features for the first 5 harmonics resulting in 20 coefficients using "Momocs::efourier()" function. Each harmonic level is made up of 4 coefficients that correspond to the effects of the cosine and sine in the x-axis (coefficients A and B) and the y-axis (coefficients C and D). To allow for discrimination between accessions based on fruit shape, principal component analysis (PCA) was performed using the "Momocs::PCA" from Momocs for EFFs. We recorded the eigenvectors of each image on the 20 resulting principal axes (Fig. 4B).

Generalized Procrustes analysis and revealed latent features

GPA describes the shape as the distance either between landmarks and a centroid. The outline of each object was decomposed into 50 evenly spaced pseudo-landmarks moving clockwise around the object. The "Momocs::fgProcrustes()" function from Momocs v$1.2.9$ [101] was used to perform the alignment between shapes (Fig. 4C; left). Each of the 50 aligned pseudo-landmarks was considered as an individual multi-variate feature. Each of the 50 features was centered such that the marginal mean of both axes is 0. The "stats::prcomp()" function in R was used to perform PCA on each of the 50 centered pseudo-landmarks (Fig. 4C; left and center).

Latent features from the calculated landmark principal components were constructed to describe the 4 most variable regions of the strawberry outline (i.e., tip, left side, neck, and right side) (Fig. 4C; center) with "lavaan::sem()" using the lavaan package $v0.6-5$ [102]. Use of SEM is commonly justified in the social sciences because of its ability to impute relationships (i.e., covariance) between unobserved constructs (latent variables) from observable variables. Here, we treated different pseudo-landmarks as observable variables to study the relationship between latent components of shape. Only those pseudo-landmarks with variance on PC1 greater than the median were used to manifest the four latent features (Fig. 4C; center and right). The "lavaan::predict()" function was then used to extract five latent variables: Tip, $Side_{Left}$, $Side_{Right}$, $Neck$, and finally $Shape$. Tip was manifest by a combination the first principal component of the pseudo-landmarks 1, 2, 3, 4, 5, 48, 49, and 50; $Neck$ by PC1 of landmarks 24, 25, 26, 27, 28, and 29; $Side_{Left}$ by PC1 of landmarks 11, 12, 13, 14, and 15; and $Side_{Right}$ by PC1 of landmarks 38, 39, 40, 41, 42, and 43. $Shape$ is then manifest by a combination of Tip, $Neck$, $Side_L$, and $Side_R$. The variances of the five latent variables was set to 1 for model identification. The model fit was adequate: $SRMR = 0.095$, $RMSEA = 0.071 \pm 0.002$, $CFI = 0.979$, and $TLI = 0.977$ [103]. However, the chi-squared test statistic was large ($\chi^2_{df=271} = 9724.76$; $p < 2e-16$) which likely resulted from the a large sample size. We did not perform model comparisons because our goal was to quantify a reduced, latent-space representation of observed pseudo-landmarks that minimizes the difference between the model-implied and sample covariance matrices. Each of the four latent features were calculated for all images.

EigenFruit analysis

EigenFruit features were calculated from the EigenFaces and other related PCA-based methods of [58–61, 65] and incorporated information about every pixel in a given set of images. The resulting matrix of binary image vectors was $6,874 \times 10,000$. There were only be as many non-zero PC's as there are observations (i.e., $6,874$). The "stats::prcomp()" function was used to perform PCA. We recorded the eigenvalues of the first 20 PCs. Together these 20 PCs explained 71.7% of the variance. PC1, PC2, and PC3 explained 26.8%, 12.6%, and 5.24%, respectively (Fig. 4D; left).

Biomass profile features

Biomass profile features described the shape as the sum of pixels in each row, or column, of a given image. We adopted this method from [13]. We generated the horizontal biomass profile by recording the number of black pixels in each of 100 rows. The vertical biomass profile was generated by recording the number of black pixels in each of the 100 columns. The function "stats::prcomp()" in R was used to perform PCA for each profile (i.e., vertical and horizontal). The eigenvectors of the first 5 PCs from each were retained. Together these 5 PCs explained 95.9% and 95.4% of the total symmetric shape variance for the horizontal and vertical profiles, respectively (Fig. 4D; center and right).

Broad-sense Heritability Estimation

Qualitative Features

Broad-sense heritability on a clone-mean basis (H^2) for each ordered level of k was estimated using the ordinal package v$2019.3-9$ [72] in R $3.5.3$. Variance components were estimated using a cumulative link mixed models with a cumulative logit link function and a multinomial error,

$$y_{ijk_l} = \mu + G_i + H_j + B_k + E_{ijk} + F_{ijk_l} \quad (4)$$

y_{ijk_l} is the categorical feature, μ is the grand mean, G_i is the random effect of ith genotype ($G_i \sim \mathcal{N}(0, \sigma_G^2)$), H_j is the fixed effect of the jth harvest, B_k is the fixed effect of the kth block, E_{ijk} is the residual error of the ijkth plot ($E_{ijk} \sim \mathcal{N}(0, \sigma_E^2)$), and F_{ijkl} is the error of ijk_lth fruit (subsample) ($F_{ijk_l} \sim \text{logit}[P(Y \leq j)]_0^{k-1}$), where k is the number of clusters. The "clmm()" function implements of cumulative link mixed models for ordinal data. Ordinal GLMMs were considered the most appropriate, and conservative, approach because we could not assume that shape categories would be linear. Variance component estimation is performed via maximum likelihood and allows for multiple random effects with crossed and nested structures [72]. H^2 for each feature was calculated as

$$H^2 = \frac{\sigma_G^2}{\sigma_G^2 + \frac{\sigma_E^2}{hr}} \quad (5)$$

Where σ_G^2 is the genetic variance, σ_E^2 is the residual variance, h is the harmonic mean of observed harvest dates per genotype (1.66), and r is the harmonic mean of replicates per harvest (2.50).

Quantitative Features

Broad-sense heritability on a clone-mean basis (H^2) was estimated for features with the lme4 package v$1.1-19$ [74] in R $3.5.3$. REML variance components were estimated using the linear mixed effect model,

$$y_{ijk} = \mu + G_i + H_j + B_k + E_{ijk} \quad (6)$$

y_{ijk} is the quantitative feature, μ is the grand mean, G_i is the random effect of the ith genotype ($G_i \sim \mathcal{N}(0, \sigma_G^2)$), H_j is the fixed effect of the jth harvest, B_k is the fixed effect of the kth block, E_{ijk} is the residual error of the ijkth plot ($E_{ijk} \sim \mathcal{N}(0, \sigma_E^2)$). Only two Harvest dates and three Blocks were observed and, because of this, they were treated as fixed effects. H^2 for each feature was

calculated as in equation 5.

Feature selection

Random forest regression models were fit in R 3.5.3 using the VSURF package v1.0.4 [73]. 100 forests, each consisting of 2,000 random trees were fit using 68 features to predict cluster assignments. The "VSURF::VSURF()" function returns two sets of features. The first includes important features with some redundancy, and the second, smaller set, corresponds to a model focusing more closely on the classification and reducing redundancy [73]. Features that appeared in the second set for more than three levels of k were recorded and used for classification for all clusters (feature set 13). Five features had mean OOB estimates greater than the median ($OOB = 0.047$) were used as feature set 5. Three features had mean OOB estimates greater than the mean estimate ($OOB = 0.12$) were recorded as feature set 3.

Classification performance

The classification accuracy was then estimated using the "MASS::lda()" function from MASS v7.3 − 51.1 [104] as well the "e1071::svm()" function from e1071 v1.7 − 0 [105]. Classification models were trained to delineate the cluster assignments from k-means using the three different feature sets as predictor variables. All images were randomly sorted into training and test sets without stratification of size 80/20%, 50/50%, and 20/80% to explore the relationship between sample size and model performance. The training set images were clustered using the "stats::kmeans()" function in R. As before, k was allowed to range from 2 to 10 for this experiment. The images in the test set were assigned to the nearest cluster for each value of k. The principal component features (i.e., EigenFruitPC[1, 7], BioVPC[1, 2], and BioHPC[1, 3]) were calculated using only the training set images and the test images were projected into this new space. The maximum number of non-zero principal components in this experiment for the EigenFruit analysis was either 5,500, 3,437, or 1,374, depending on the size of the training data set. The percent variance explained (PVE) of each leading PC was recalculated. Geometric descriptors (i.e., BAR, SI, and Kurt) were not recalculated as they are derived from an individual sample and not a sample population. Finally, both LDA and SVR models were trained using all three feature sets for all values of k using the "MASS::lda()" and "e1071::svm()" functions in R. The trained models were used to classify the images in the respective test set. The model performance was evaluated using the average classification accuracy, precision, recall, and false positive rate (FPR) of 10 iterations of cross validation.

Availability of source code and requirements

Lists the following:

- Project name: 2DShapeDescription
- Project home page: https://github.com/mjfeldmann/2DShapeDescription
- Operating system(s): Platform independent
- Programming language: R and ImageJ Macro
- Other requirements: Not Applicable
- License: MIT License.
- Any restriction to use by non-academics: none

Availability of supporting data and materials

The data supporting the results of this article are available in the Zenodo repository [66]. The code to reproduce these analyses are documented and available on GitHub [67]. Data further supporting this work are available in the GigaScience repository, GigaDB [68].

Additional files

The additional files for this article are available in the Zenodo repository [66].

- **Additional file 1: Fig. S1** Results of PPKC against original cluster assignments. Ordered centroids from $k = 2$ to $k = 8$. On the left are the unordered assignments from k-means, and the on the right are the order assignments following PPKC. Cluster position indicated on the right [1, 8].
- **Additional file 2: Fig. S2** Optimal Value of k. **(A)** Total within cluster sum of squares. **(B)** Inverse of the Adjusted R^2. **(C)** Akaike information criterion (AIC). **(D)** Bayesian information criterion (AIC). All metrics were calculated on a random sample of 3,437 images (50%). 10 samples were randomly drawn. The vertical dashed line in each plot represents the optimal value of k. Reported metrics are standardized to be between [0, 1].
- **Additional file 3: Fig. S3** Hierarchical clustering and distance between classes on PC1. The relationship between clusters at each value of k is represented as both a dendrogram and as bar plot. The labels on the dendrogram (i.e., V1, V2, V3,..., V10) represent the original cluster assignment from k-means. The barplot to the right of each dendrogram depicts the elements of the eigenvector associated with the largest eigenvalue form PPKC. The labels above each line represent the original cluster assignment.
- **Additional file 4: Fig. S4** BLUPs for 13 selected features. For each plot, the X-axis is the index and the Y-axis is the BLUP value estimated from a linear mixed model. Grey points represent the mean feature value for each individual. Each point is the BLUP for a single genotype.
- **Additional file 5: Fig. S5** Effects of Eigenfruit, Vertical Biomass, and Horizontal Biomass Analyses. **(A)** Effects of PC [1, 7] from the Eigenfruit analysis on the mean shape (center column). Left column is the mean shape minus 1.5× the standard deviation. Right is the mean shape plus 1.5× the standard deviation. The horizontal axis is the horizontal pixel position. The vertical axis is the vertical pixel position. **(B)** Effects of PC [1, 3] from the Horizontal Biomass analysis on the mean shape (center column). Left column is the mean shape minus 1.5× the standard deviation. Right is the mean shape plus 1.5× the standard deviation. The horizontal axis is the vertical position from the image (height). The vertical axis is the number of activated pixels (RowSum) at the given veritcal position. **(C)** Effects of PC [1, 3] from the Vertical Biomass analysis on the mean shape (center column). Left column is the mean shape minus 1.5× the standard deviation. Right is the mean shape plus 1.5× the standard deviation. The horizontal axis is the horizontal position from the image (width). The vertical axis is the number of activated pixels (ColSum) at the given horizontal position.
- **Additional file 6: Fig. S6** PPKC with variable sample size. Ordered centroids from $k = 2$ to $k = 5$ using different image sets for clustering. For all $k = [2, 5]$, k-means clustering was performed using either 100, 80, 50%, or 20% of the total number of images; 6,874, 5,500, 3,437, and 1,374 respectively. Cluster position indicated on the right [1, 5].
- **Additional file 7: Fig. S7** Comparison to RosBREED scale. **(A.)** RosBREED 9-unit ordinal scale. **(B.)** PPKC 4-unit ordinal scale. **(C.)** Distributions of selected feature with each level of $k = 4$ from the PPKC 4-unit ordinal scale. The light gray line is cluster 1, medium gray line is cluster 2, dark gray line is cluster 3, and black line is cluster 4.

Declarations

CA: Correspondence Analysis; EFA: Elliptical Fourier Analysis; FPR: False Positive Rate; GM: Geometric Morphometrics; GPA: Generalized Procrustes Analysis; LDA: Linear Discriminant Analysis; LSP: Latent Space Phenotype; MDS: Multi-Dimensional Scaling; OOB: Out-of-Bag Error; PC: Principal Component; PH: Persistent Homology; PPKC: Principal Progression of K Clusters; PVE: Percent Variance Explained; QTL: Quantitative Trait LocusSEM: Structure Equation Model; SIOX: Simple Interactive Object Extraction; SVR: Support Vector Regression; VSURF: Variable Selection Using Random Forests.

Competing Interests

The author(s) declare that they have no competing interests.

Funding

This research was supported by grants to S.J.K. from the United States Department of Agriculture (http://dx.doi.org/10.13039/100000199) National Institute of Food and Agriculture (NIFA) Specialty Crops Research Initiative (# 2017-51181-26833) and California Strawberry Commission (http://dx.doi.org/10.13039/100006760), in addition to funding from the University of California, Davis (http://dx.doi.org/10.13039/100007707).

Author's Contributions

The overall project was conceived by MJF and SJK; MAH, RAF, CML, and GSC helped grow plant material and collect raw data; MJF performed the analyses; MJF, AT, and SJK wrote the paper.

Acknowledgements

We thank Bruce Campopiano and Eduardo Garcia for assistance with several aspects of the field experiments. We thank Patrick J. Brown, Daniel H. Chitwood, Christine H. Diepenbrock, Sarah D. Turner, and Daniel E. Runcie for their comments and advice and for reviewing this manuscript.

Opinions, findings, conclusions, or recommendations expressed in this publication are those of the authors and do not necessarily reflect the views of the USDA. USDA is an equal opportunity provider and employer.

References

1. Duchesne A. Histoire naturelle des fraisiers. Didot le Jeune, Paris.; 1766.
2. Darrow GM. The strawberry. History, breeding and physiology. Holt, Rinehart and Winston, New York; 1966.
3. Edger PP, Poorten TJ, VanBuren R, Hardigan MA, Colle M, McKain MR, et al. Origin and evolution of the octoploid strawberry genome. Nature Genetics 2019 Mar;51(3):541–547.
4. Hardigan MA, Poorten TJ, Acharya CB, Cole GS, Hummer KE, Bassil N, et al. Domestication of Temperate and Coastal Hybrids with Distinct Ancestral Gene Selection in Octoploid Strawberry. The Plant Genome 2018;11(3):0.
5. Aharoni A. Gain and Loss of Fruit Flavor Compounds Produced by Wild and Cultivated Strawberry Species. The Plant Cell 2004 Nov;16(11):3110–3131.
6. Wang SY, Lewers KS. Antioxidant Capacity and Flavonoid Content in Wild Strawberries. Journal of the American Society for Horticultural Science 2007 Sep;132(5):629–637.
7. Diamanti J, Capocasa F, Balducci F, Battino M, Hancock J, Mezzetti B. Increasing Strawberry Fruit Sensorial and Nutritional Quality Using Wild and Cultivated Germplasm. PLoS ONE 2012 Oct;7(10):e46470.
8. Vallarino JG, de Abreu e Lima F, Soria C, Tong H, Pott DM, Willmitzer L, et al. Genetic diversity of strawberry germplasm using metabolomic biomarkers. Scientific Reports 2018 Dec;8(1).
9. Liao X, Li M, Liu B, Yan M, Yu X, Zi H, et al. Interlinked regulatory loops of ABA catabolism and biosynthesis coordinate fruit growth and ripening in woodland strawberry. Proceedings of the National Academy of Sciences 2018 Dec;115(49):E11542–E11550.
10. Whitaker VM, Hasing T, Chandler CK, Plotto A, Baldwin E. Historical Trends in Strawberry Fruit Quality Revealed by a Trial of University of Florida Cultivars and Advanced Selections. HortScience 2011 Apr;46(4):553–557.
11. Visa S, Cao C, Gardener BM, van der Knaap E. Modeling of tomato fruits into nine shape categories using elliptic fourier shape modeling and Bayesian classification of contour morphometric data. Euphytica 2014 Dec;200(3):429–439.
12. Migicovsky Z, Gardner KM, Money D, Sawler J, Bloom JS, Moffett P, et al. Genome to Phenome Mapping in Apple Using Historical Data. The Plant Genome 2016;9(2):0.
13. Turner SD, Ellison SL, Senalik DA, Simon PW, Spalding EP, Miller ND. An Automated Image Analysis Pipeline Enables Genetic Studies of Shoot and Root Morphology in Carrot (Daucus carota L.). Frontiers in Plant Science 2018 Nov;9.
14. Mathey MM, Mookerjee S, Gündüz K, Hancock JF, Iezzoni AF, Mahoney LL, et al. Large-Scale Standardized Phenotyping of Strawberry in RosBREED. Journal of the American Pomological Society 2013;p. 12.
15. Whitaker VM, Osorio LF, Hasing T, Gezan S. Estimation of Genetic Parameters for 12 Fruit and Vegetative Traits in the University of Florida Strawberry Breeding Population. Journal of the American Society for Horticultural Science 2012 Sep;137(5):316–324.
16. Antanaviciute L. Genetic mapping and phenotyping plant characteristics, fruit quality and disease resistance traits in octoploid strawberry (Fragaria× ananassa). PhD thesis, University of Reading; 2016.
17. Minamikawa MF, Nonaka K, Kaminuma E, Kajiya-Kanegae H, Onogi A, Goto S, et al. Genome-wide association study and genomic prediction in citrus: potential of genomics-assisted breeding for fruit quality traits. Scientific reports 2017;7(1):4721.
18. Simpson MG. 9. In: Plant systematics Academic press; 2010. p. 494–508.
19. Victorino J, Gómez F. Contour analysis for interpretable leaf shape category discovery. Plant Methods 2019;15(1):112.
20. Ishikawa T, Hayashi A, Nagamatsu S, Kyutoku Y, Dan I, Wada T, et al. Classification of Strawberry Fruit Shape by Machine Learning. ISPRS - International Archives of the Photogrammetry, Remote Sensing and Spatial Information Sciences 2018 May;XLII-2:463–470.
21. dos Anjos RS, Marçal TdS, Carneiro P, Carneiro JEdS. New Proposals to Estimate Unbiased Selection Gain and Coefficient of Variation in Traits Evaluated Using Score Scales. Crop Science 2019;.
22. Mitry D, Zutis K, Dhillon B, Peto T, Hayat S, Khaw KT, et al. The accuracy and reliability of crowdsource annotations of digital retinal images. Translational Vision Science and Technology 2016;5(5):6–6.
23. Zhou N, Siegel ZD, Zarecor S, Lee N, Campbell DA, Andorf CM, et al. Crowdsourcing image analysis for plant phenomics to generate ground truth data for machine learning. PLoS Computational Biology 2018;14(7):e1006337.
24. Chollet F, Allaire JJ. Deep Learning with R. 1st ed. Greenwich, CT, USA: Manning Publications Co.; 2018.
25. Achcar F, Camadro JM, Mestivier D. AutoClass@ IJM: a powerful tool for Bayesian classification of heterogeneous data in biology. Nucleic acids research 2009;37(suppl_2):W63–W67.
26. Cheverud JM, Buikstra JE. Quantitative genetics of skeletal nonmetric traits in the rhesus macaques on Cayo Santiago. II. Phenotypic, genetic, and environmental correlations between traits. American Journal of Physical Anthropology 1981;54(1):51–58.
27. Agresti A. Analysis of ordinal categorical data, vol. 656. John Wiley & Sons; 2010.
28. Montesinos-López OA, Montesinos-López A, Pérez-Rodríguez P, de los Campos G, Eskridge K, Crossa J. Threshold Models for Genome-Enabled Prediction of Ordinal Categorical Traits in Plant Breeding. G3: Genes, Genomes, Genetics 2015 Feb;5(2):291–300.
29. Montesinos-López OA, Montesinos-López A, Crossa J, Burgueño J, Eskridge K. Genomic-Enabled Prediction of Ordinal Data with Bayesian Logistic Ordinal Regression. G3: Genes, Genomes, Genetics 2015 Oct;5(10):2113–2126.
30. Fresnedo-Ramírez J, Famula TR, Gradziel TM. Application of a Bayesian ordinal animal model for the estimation of breeding values for the resistance to Monilinia fruticola (G. Winter) Honey in progenies of peach [Prunus persica (L.) Batsch]. Breeding Science 2017;p. 16027.
31. Hearn DJ. Shape analysis for the automated identification of plants from images of leaves. Taxon 2009 Aug;58(3):934–954.
32. Fu G, Berg A, Das K, Li J, Li R, Wu R. A statistical model for mapping morphological shape. Theoretical Biology and Medical Modelling 2010;7(1):28.
33. Balduzzi M, Binder BM, Bucksch A, Chang C, Hong L, Iyer-Pascuzzi AS, et al. Reshaping Plant Biology: Qualitative and Quantitative Descriptors for Plant Morphology. Frontiers in Plant Science 2017 Feb;08.
34. Tanksley SD. The genetic, developmental, and molecular bases of fruit size and shape variation in tomato. The plant cell 2004;16(suppl 1):S181–

S189.
35. Monforte AJ, Diaz A, Caño-Delgado A, van der Knaap E. The genetic basis of fruit morphology in horticultural crops: lessons from tomato and melon. Journal of Experimental Botany 2013 Aug;65(16):4625–4637.
36. Xiao H, Jiang N, Schaffner E, Stockinger EJ, Van Der Knaap E. A retrotransposon-mediated gene duplication underlies morphological variation of tomato fruit. Science 2008;319(5869):1527–1530.
37. Wu S, Zhang B, Keyhaninejad N, Rodríguez GR, Kim HJ, Chakrabarti M, et al. A common genetic mechanism underlies morphological diversity in fruits and other plant organs. Nature Communications 2018 Dec;9(1).
38. Han K, Jeong HJ, Yang HB, Kang SM, Kwon JK, Kim S, et al. An ultra-high-density bin map facilitates high-throughput QTL mapping of horticultural traits in pepper (Capsicum annuum). DNA Research 2016;23(2):81–91.
39. Chunthawodtiporn J, Hill T, Stoffel K, Van Deynze A. Quantitative trait loci controlling fruit size and other horticultural traits in bell pepper (Capsicum annuum). The Plant Genome 2018;11(1).
40. White AG, Alspach PA, Weskett RH, Brewer LR. Heritability of fruit shape in pears. Euphytica 2000 Mar;112(1):1–7.
41. Prashar A, Hornyik C, Young V, McLean K, Sharma SK, Dale MFB, et al. Construction of a dense SNP map of a highly heterozygous diploid potato population and QTL analysis of tuber shape and eye depth. Theoretical and Applied Genetics 2014 Oct;127(10):2159–2171.
42. Lerceteau-Köhler E, Moing A, Guérin G, Renaud C, Petit A, Rothan C, et al. Genetic dissection of fruit quality traits in the octoploid cultivated strawberry highlights the role of homoeo-QTL in their control. Theoretical and Applied Genetics 2012;124(6):1059–1077.
43. Lynch M, Walsh B, et al. Genetics and analysis of quantitative traits, vol. 1. Sinauer Sunderland, MA; 1998.
44. Goddard M, Hayes B. Genomic selection. Journal of Animal breeding and Genetics 2007;124(6):323–330.
45. Heffner EL, Sorrells ME, Jannink JL. Genomic selection for crop improvement. Crop Science 2009;49(1):1–12.
46. Resende MF, Muñoz P, Resende MD, Garrick DJ, Fernando RL, Davis JM, et al. Accuracy of genomic selection methods in a standard data set of loblolly pine (Pinus taeda L.). Genetics 2012;190(4):1503–1510.
47. Tanabata T, Shibaya T, Hori K, Ebana K, Yano M. SmartGrain: high-throughput phenotyping software for measuring seed shape through image analysis. Plant physiology 2012;160(4):1871–1880.
48. Claude J. Morphometrics with R. Springer Science & Business Media; 2008.
49. Kuhl FP, Giardina CR. Elliptic Fourier features of a closed contour. Computer Graphics and Image Processing 1982;18(3):236–258.
50. Chitwood DH, Ranjan A, Martinez CC, Headland LR, Thiem T, Kumar R, et al. A Modern Ampelography: A Genetic Basis for Leaf Shape and Venation Patterning in Grape. Plant Physiology 2014 Jan;164(1):259–272.
51. Chitwood DH, Otoni WC. Morphometric analysis of Passiflora leaves: the relationship between landmarks of the vasculature and elliptical Fourier descriptors of the blade. GigaScience 2017 Jan;6(1).
52. Gower JC. Generalized procrustes analysis. Psychometrika 1975;40(1):33–51.
53. Bookstein FL. Landmark methods for forms without landmarks: morphometrics of group differences in outline shape. Medical Image Analysis 1997 Apr;1(3):225–243.
54. Klingenberg CP, Leamy LJ. Quantitative Genetics of Geometric Shape in the Mouse Mandible. Evolution 2001;55(11):2342–2352.
55. Langlade NB, Feng X, Dransfield T, Copsey L, Hanna AI, Thébaud C, et al. Evolution through genetically controlled allometry space. Proceedings of the National Academy of Sciences 2005;102(29):10221–10226.
56. Bensmihen S, Hanna AI, Langlade NB, Micol JL, Bangham A, Coen ES. Mutational spaces for leaf shape and size. Hfsp Journal 2008;2(2):110–120.
57. Manacorda CA, Asurmendi S. Arabidopsis phenotyping through geometric morphometrics. GigaScience 2018;7(7):giy073.
58. Sirovich L, Kirby M. Low-dimensional procedure for the characterization of human faces. Journal of the Optical Society of America 1987 Mar;4(3):519.
59. Turk MA, Pentland AP. Face recognition using eigenfaces. In: Proceedings. 1991 IEEE Computer Society Conference on Computer Vision and Pattern Recognition; 1991. p. 586–591.
60. Horgan GW, Talbot M, Davey JC. Use of statistical image analysis to discriminate carrot cultivars. Computers and Electronics in Agriculture 2001;31(2):191–199.
61. Horgan GW. The statistical analysis of plant part appearance—a review. Computers and Electronics in Agriculture 2001;31(2):169–190.
62. Ehsanirad A. Plant classification based on leaf recognition. International Journal of Computer Science and Information Security 2010;8(4):78–81.
63. Rodrigo R, Samarawickrame K, Mindya S. An Intelligent Flower Analyzing System for Medicinal Plants. Conference on Computer Graphics, Visualization and Computer Vision 2013;p. 4.
64. Ubbens J, Cieslak M, Prusinkiewicz P, Parkin I, Ebersbach J, Stavness I. Latent Space Phenotyping: Automatic Image-Based Phenotyping for Treatment Studies. bioRxiv 2019;p. 557678.
65. Gage JL, Richards E, Lepak N, Kaczmar N, Soman C, Chowdhary G, et al. In-field whole plant maize architecture characterized by Latent Space Phenotyping. bioRxiv 2019;p. 763342.
66. Feldmann MJ. Classification and Quantification of Strawberry Fruit Shape Data; 2019, http://dx.doi.org/10.5281/zenodo.3365714.
67. Feldmann MJ. 2DShapeDescription; 2019, https://github.com/mjfeldmann/2DShapeDescription.
68. Feldmann MJ, Hardigan MA, Famula RA, López CM, Tabb A, Cole GS, et al. Supporting data for "Multi-Dimensional Machine Learning Approaches for Fruit Shape Phenotyping in Strawberry"; 2020, http://dx.doi.org/10.5524/100707.
69. Lloyd SP. Least squares quantization in pcm. IEEE Transactions on Information Theory 1982;28:129–137.
70. Evanno G, Regnaut S, Goudet J. Detecting the number of clusters of individuals using the software STRUCTURE: a simulation study. Molecular Ecology 2005;14(8):2611–2620.
71. Pincot DD, Poorten TJ, Hardigan MA, Harshman JM, Acharya CB, Cole GS, et al. Genome-wide association mapping uncovers Fw1, a dominant gene conferring resistance to Fusarium wilt in strawberry. G3: Genes, Genomes, Genetics 2018;8(5):1817–1828.
72. Christensen RHB. ordinal—Regression Models for Ordinal Data; 2019, r package version 2019.3-9. http://www.cran.r-project.org/package = ordinal/.
73. Genuer R, Poggi JM, Tuleau-Malot C. VSURF: an R package for variable selection using random forests. The R Journal 2015;7(2):19–33.
74. Bates D, Mächler M, Bolker B, Walker S. Fitting Linear Mixed-Effects Models Using lme4. Journal of Statistical Software 2015;67(1):1–48.
75. Li M, Frank MH, Coneva V, Mio W, Chitwood DH, Topp CN. The persistent homology mathematical framework provides enhanced genotype-to-phenotype associations for plant morphology. Plant physiology 2018;177(4):1382–1395.
76. Bernardo R, Thompson AM. Germplasm architecture revealed through chromosomal effects for quantitative traits in maize. The plant Genome 2016;9(2).
77. Voth V, Bringhurst RS. Strawberry plant called Chandler; 1984, uS Patent App. 06/452,699.
78. Voth V, Shaw DV, Bringhurst RS. Strawberry plant called Camarosa; 1994, uS Patent App. 08/041,742.
79. Suenaga T, Imamura Y, Maeda K, Yamada T, Takamatsu M. The workloads of farmers who sort and pack strawberries in accordance with standards of shipment and their awareness of standards of shipment. Journal of the Japanese Association of Rural Medicine 1989;38(4):895–907.
80. Li Z, Sillanpää MJ. Dynamic quantitative trait locus analysis of plant phenomic data. Trends in plant science 2015;20(12):822–833.
81. Chitwood DH, Topp CN. Revealing plant cryptotypes: defining meaningful phenotypes among infinite traits. Current opinion in plant biology 2015;24:54–60.
82. Sonnenschein A, VanderZee D, Pitchers WR, Chari S, Dworkin I. An image database of Drosophila melanogaster wings for phenomic and biometric analysis. GigaScience 2015;4(1):25.
83. Granier C, Vile D. Phenotyping and beyond: modelling the relationships

between traits. Current opinion in plant biology 2014;18:96–102.

84. Li M, An H, Angelovici R, Bagaza C, Batushansky A, Clark L, et al. Topological Data Analysis as a Morphometric Method: Using Persistent Homology to Demarcate a Leaf Morphospace. Frontiers in Plant Science 2018 Apr;9.
85. Iwata H, Ebana K, Uga Y, Hayashi T. Genomic prediction of biological shape: elliptic fourier analysis and kernel partial least squares (PLS) regression applied to grain shape prediction in rice (Oryza sativa L.). PloS one 2015;10(3):e0120610.
86. Migicovsky Z, Gardner KM, Money D, Sawler J, Bloom JS, Moffett P, et al. Genome to phenome mapping in apple using historical data. The plant genome 2016;9(2).
87. Kumar S, Kirk C, Deng CH, Shirtliff A, Wiedow C, Qin M, et al. Marker-trait associations and genomic predictions of interspecific pear (Pyrus) fruit characteristics. Scientific reports 2019;9(1):9072.
88. Syms C. Ordination. In: Encyclopedia of Ecology Elsevier; 2008.p. 2572–2581.
89. Jiang N, Gao D, Xiao H, Van Der Knaap E. Genome organization of the tomato sun locus and characterization of the unusual retrotransposon Rider. The Plant Journal 2009;60(1):181–193.
90. Frary A, Nesbitt TC, Frary A, Grandillo S, Van Der Knaap E, Cong B, et al. fw2. 2: a quantitative trait locus key to the evolution of tomato fruit size. Science 2000;289(5476):85–88.
91. Liu J, Van Eck J, Cong B, Tanksley SD. A new class of regulatory genes underlying the cause of pear-shaped tomato fruit. Proceedings of the National Academy of Sciences 2002;99(20):13302–13306.
92. Rodríguez GR, Muños S, Anderson C, Sim SC, Michel A, Causse M, et al. Distribution of SUN, OVATE, LC, and FAS in the tomato germplasm and the relationship to fruit shape diversity. Plant physiology 2011;156(1):275–285.
93. Rodríguez GR, Kim HJ, Van Der Knaap E. Mapping of two suppressors of OVATE (sov) loci in tomato. Heredity 2013;111(3):256.
94. Lande R, Thompson R. Efficiency of marker-assisted selection in the improvement of quantitative traits. Genetics 1990;124(3):743–756.
95. Wang F. SIOX plugin in ImageJ: area measurement made easy. UV4 Plants Bulletin 2017 Feb;2:37–44.
96. Schneider CA, Rasband WS, Eliceiri KW. NIH Image to ImageJ: 25 years of image analysis. Nature Methods 2012 Jul;9(7):671–675.
97. Schindelin J, Arganda-Carreras I, Frise E, Kaynig V, Longair M, Pietzsch T, et al. Fiji: an open-source platform for biological-image analysis. Nature methods 2012;9(7):676.
98. R Core Team. R: A Language and Environment for Statistical Computing. R Foundation for Statistical Computing, Vienna, Austria; 2019, https://www.R-project.org/.
99. Urbanek S. jpeg: Read and write JPEG images; 2014, r package version 0.1-8.
100. Ooms J. magick: Advanced Graphics and Image-Processing in R; 2018, r package version 2.0.
101. Bonhomme V, Picq S, Gaucherel C, Claude J. Momocs: Outline Analysis Using R. Journal of Statistical Software 2014;56(13):1–24.
102. Rosseel Y. lavaan: An R Package for Structural Equation Modeling. Journal of Statistical Software 2012;48(2):1–36.
103. Schreiber JB, Nora A, Stage FK, Barlow EA, King J. Reporting Structural Equation Modeling and Confirmatory Factor Analysis Results: A Review. The Journal of Educational Research 2006 Jul;99(6):323–338.
104. Venables WN, Ripley BD. Modern Applied Statistics with S. Fourth ed. New York: Springer; 2002. ISBN 0-387-95457-0.
105. Meyer D, Dimitriadou E, Hornik K, Weingessel A, Leisch F. e1071: Misc Functions of the Department of Statistics, Probability Theory Group (Formerly: E1071), TU Wien; 2019, r package version 1.7-0.1.

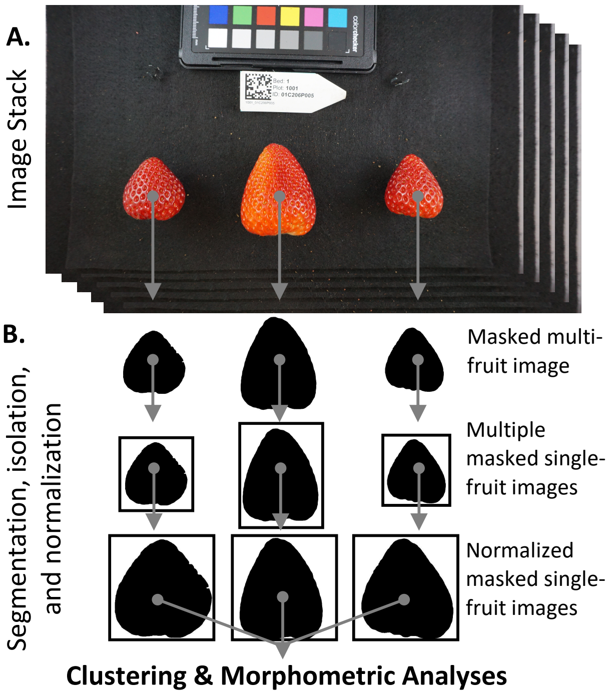

Figure 1. An example of the processing pipeline. **(A)** A user collects a stack of images containing multiple strawberries and a unique QR code. **(B)** All images are then segmented using the SIOX algorithm implemented in ImageJ. Each object is then cut from its original image based on the coordinates of its bounding rectangle in R $3.5.3$. White pixels are then added to the edges of each frame until all images are 1000×1000 pixels. Regions of interest are then scaled such that the major axis of each object becomes 1000px in ImageJ. Output images are scale invariant and maintain the original aspect ratio.

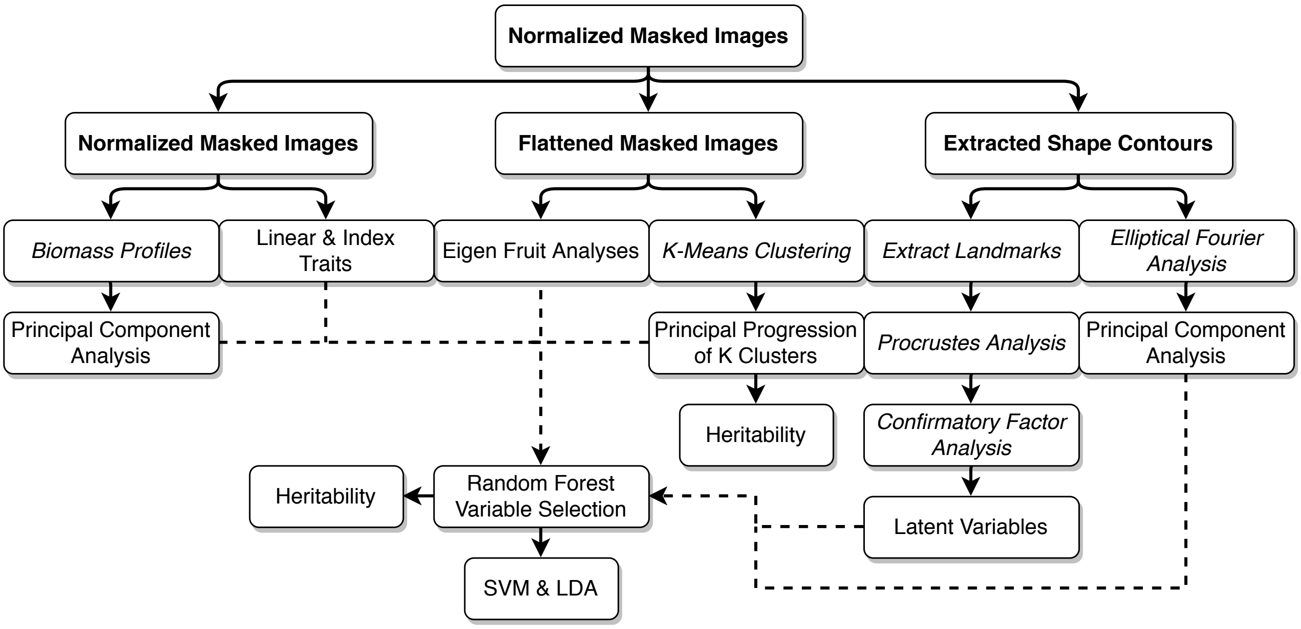

Figure 2. Analysis pipeline for this study. All images start as normalized, binary images from Fig 1. Images then follow each of the paths through different morphometric feature extractions including linear geometric features, Biomass Profile analysis, EigenFruit analysis, Procrustes analysis, and Elliptical Fourier analysis as either normalized or flattened images (e.g., linear, BPA, and EigenFruit analysis) or as shape contours (e.g., GPA and EFA). Flattened binary images are used to perform k-means clustering and subsequently PPKC.

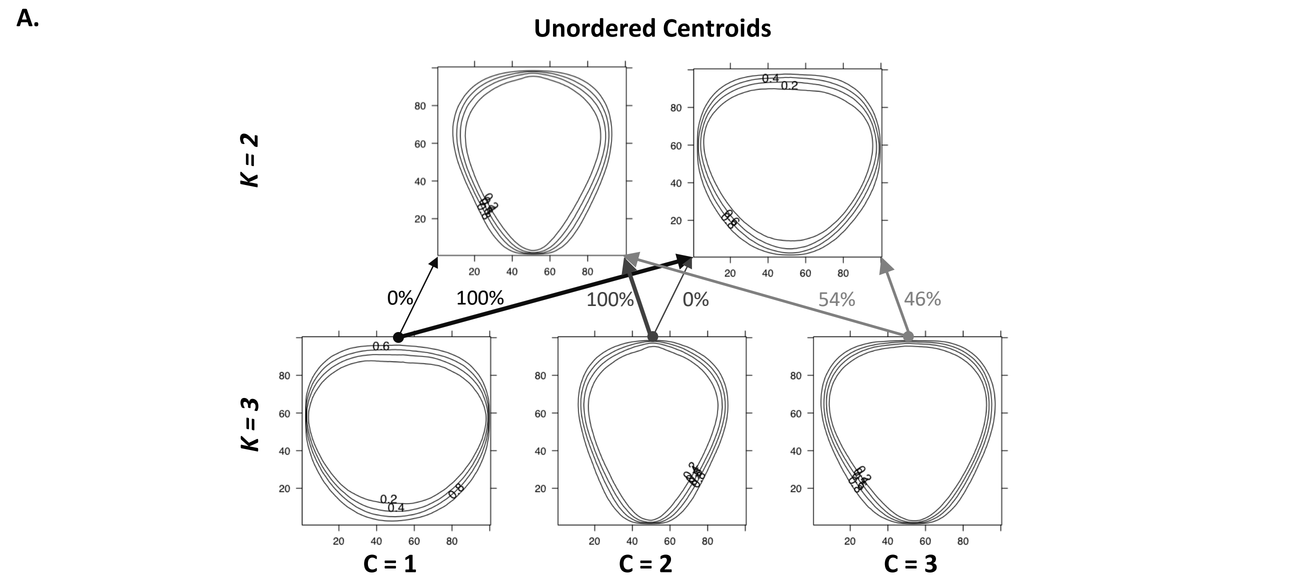

B.

M

	k3$_{c1}$	k3$_{c2}$	k3$_{c3}$
k2$_{c1}$	0	1	0.54
k2$_{c2}$	1	0	0.46

Σ_M

	k3$_{c1}$	k3$_{c2}$	k3$_{c3}$
k3$_{c1}$	0.5	-0.5	0.040
k3$_{c2}$	-0.5	0.5	-0.04
k3$_{c3}$	0.04	-0.04	0.003

Eigen Decomposition

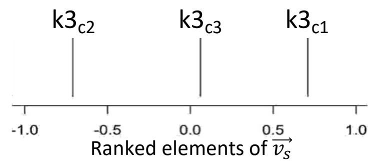

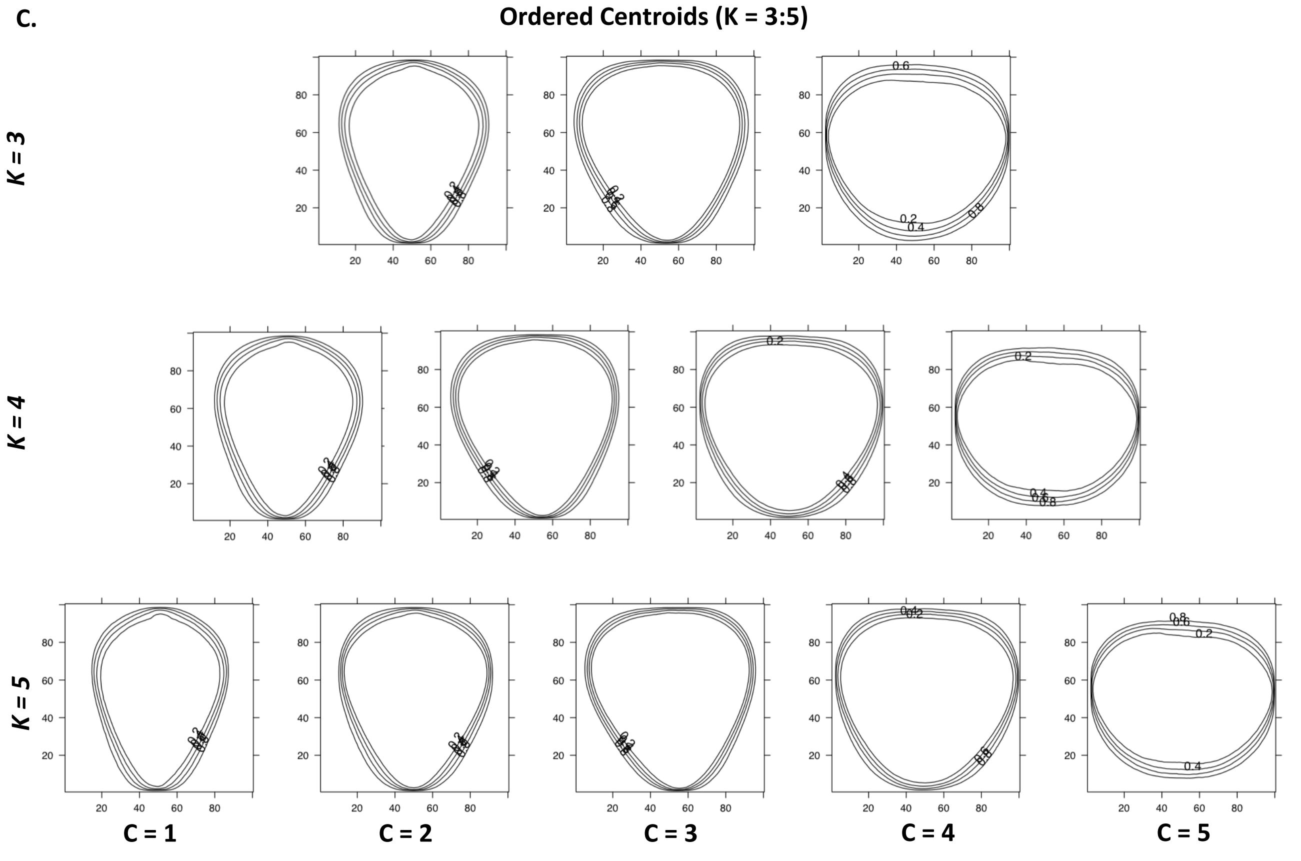

Figure 3. An example use of PPKC. **(A)** After k-means clustering is performed clusters are randomly assigned a numeric value ($1, 2, \ldots, k$). When $k > 2$, this value becomes nominal. PPKC relies on the fact that the order through clusters when $k = 2$ has identical interpretations in either direction. The lines representing each clusters centroid reflect the 20th, 40th, 60th, and 80th quantiles, moving out from the center of each images. **(B)**(1) A table representation of the resultant matrix from equation 1. Each cell represents the proportion of images in the column class and in the row class, normalized by the number of images in the column class. **(B)**(2) A table representation of $\Sigma_{\mathbf{M}}$. **(B)**(3) The ranked elements of $\vec{v}_s$ shown on a number line. **(C)** After using PPKC, the order of groups is explicitly identified. In this example, showing $k = [3, 5]$, the order discovered seems to trend from tall and thin berries, through more triangular shapes, and ending with berries that are short and wide.

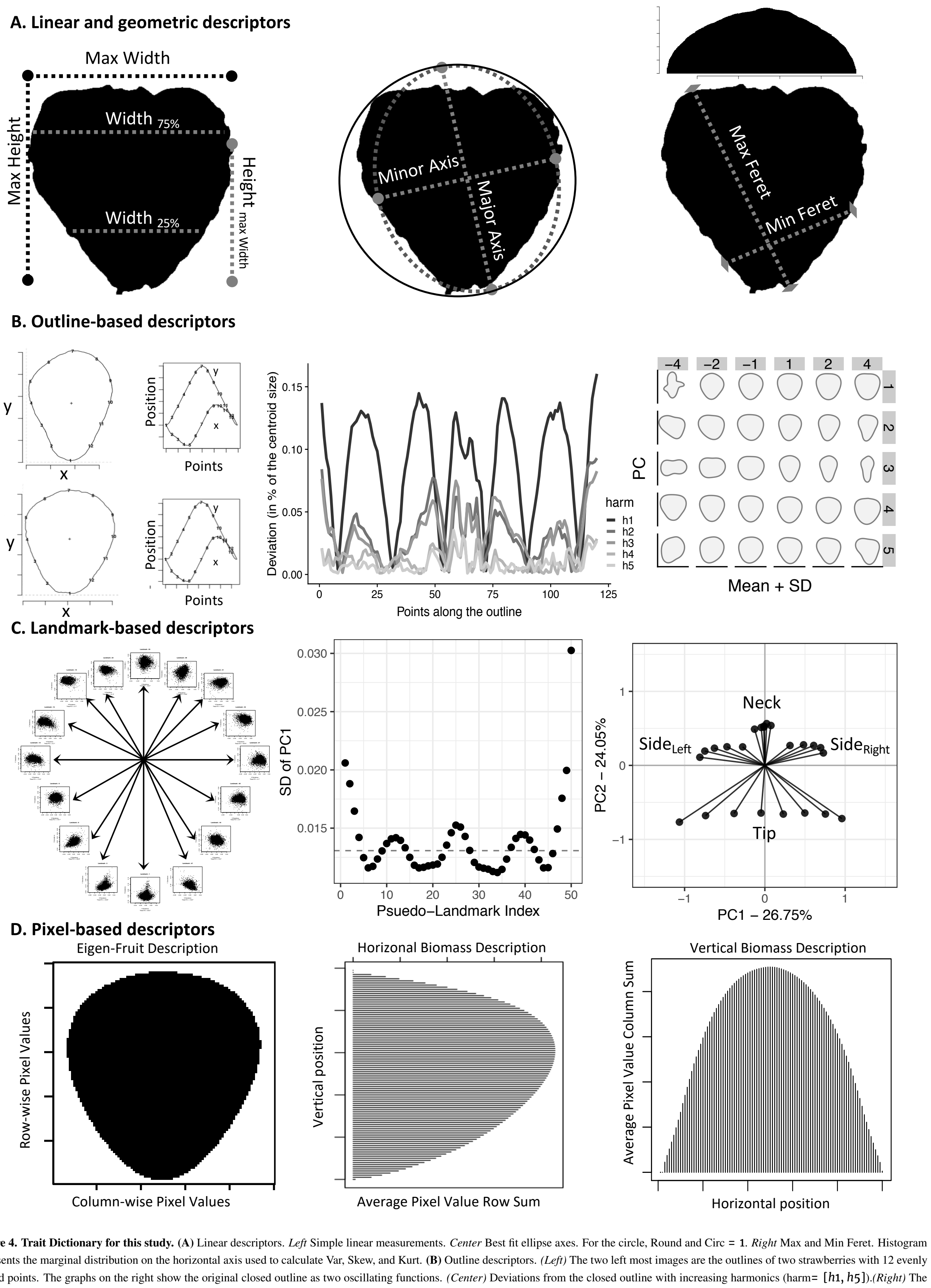

Figure 4. Trait Dictionary for this study. **(A)** Linear descriptors. *Left* Simple linear measurements. *Center* Best fit ellipse axes. For the circle, Round and Circ $= 1$. *Right* Max and Min Feret. Histogram represents the marginal distribution on the horizontal axis used to calculate Var, Skew, and Kurt. **(B)** Outline descriptors. *(Left)* The two left most images are the outlines of two strawberries with 12 evenly spaced points. The graphs on the right show the original closed outline as two oscillating functions. *(Center)* Deviations from the closed outline with increasing harmonics (harm$= [h1, h5]$).*(Right)* The plot shows the effects of PC $[1, 5]$ (vertical) with effect sizes, $[-4, 4]$ (horizontal) on the mean shape. **(C)** Landmark descriptors. *(Left)* 50 evenly spaced landmarks are extracted and treated as bi-variate features.*(Center)* Standard deviation of PC1 for each landmark is plotted in sequence. Dashed horizontal line is the median standard deviation. *(Right)* Pseudo-landmarks were selected to represent each region of high variance. Using the values on the first principal axis as observed variables, confirmatory factor analysis was performed to infer latent relationships to tip, left and right side, and neck shape. **(D)** Pixel descriptors. *(Left)* Mean EigenFruit using flattened binary images. *(Center)* Mean Horizontal Biomass using image row sums. *(Right)* Mean vertical biomass using image column sums.

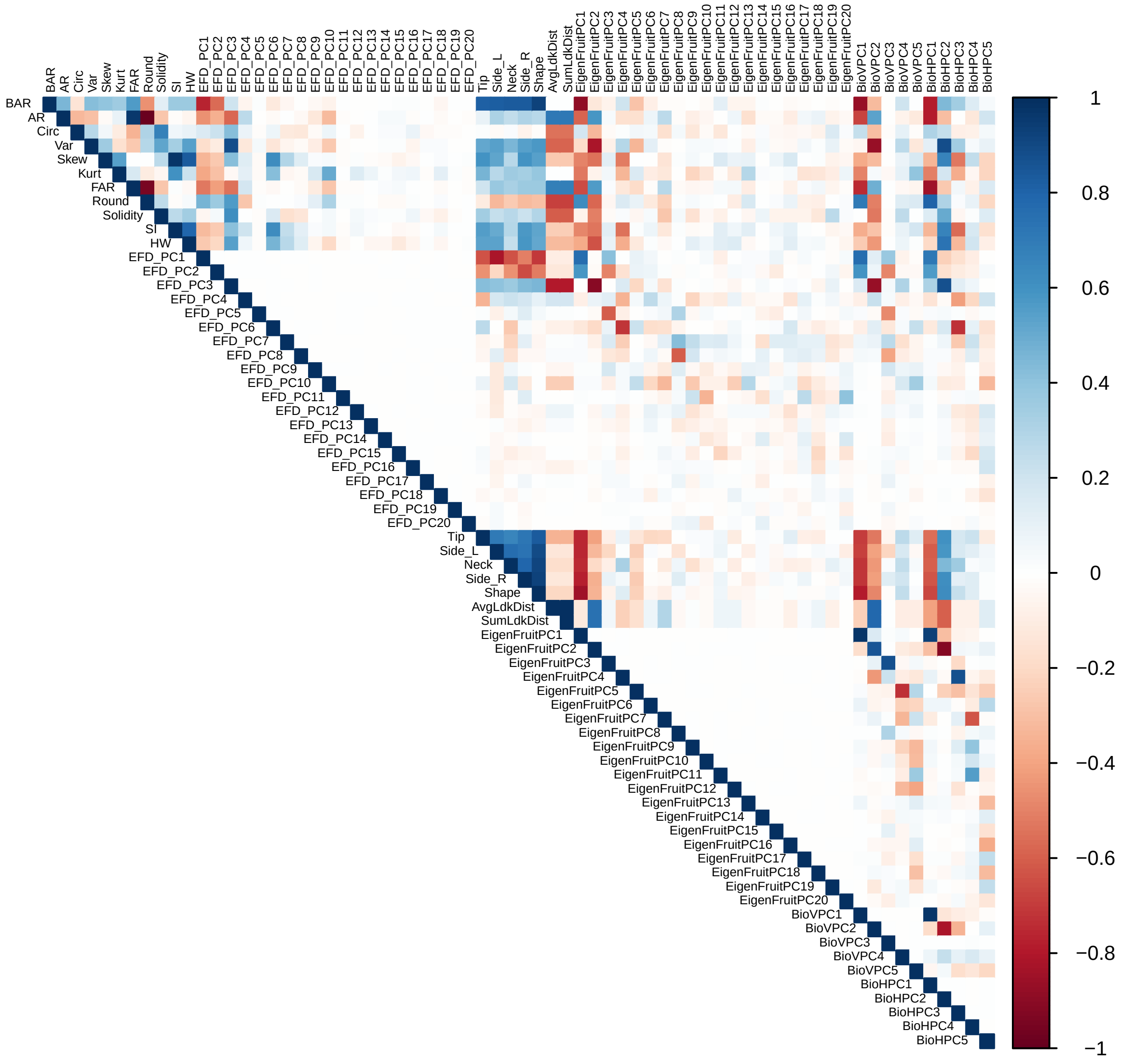

Figure 5. Correlations between all 68 features used in this study. Positive correlations are colored blue, negative correlations are colored red.

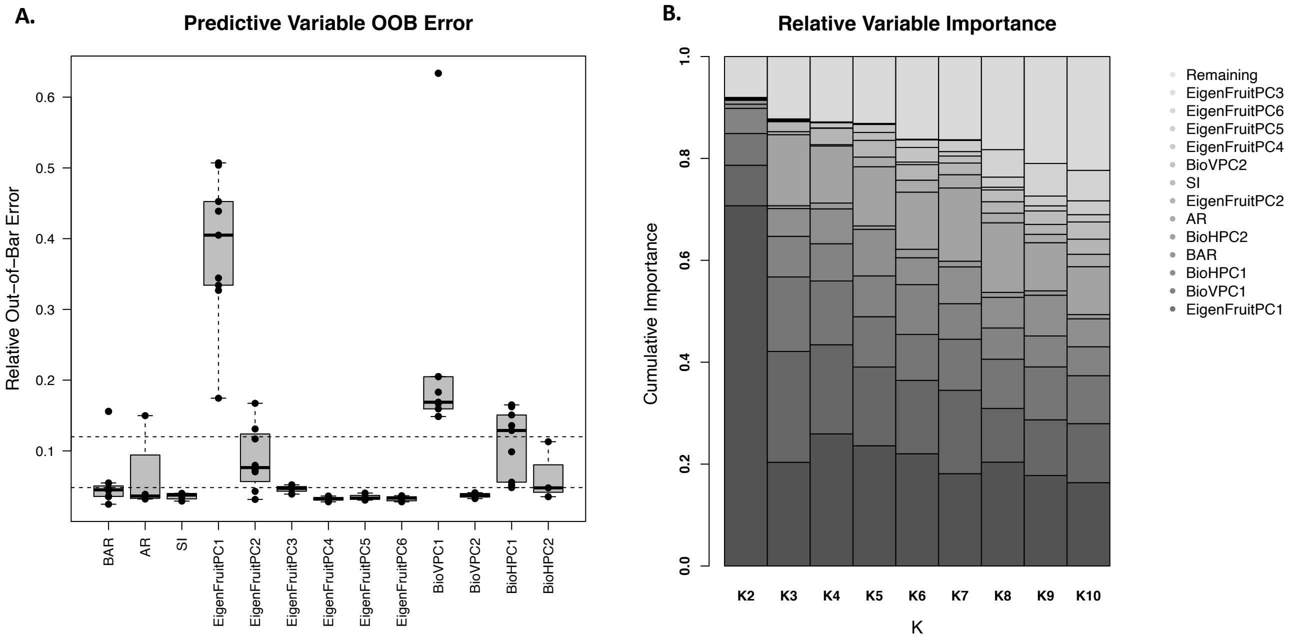

Figure 6. Results from feature selection. **(A)** Out-of-Bag error for each of the 13 selected features. Horizontal dashed lines are the median (0.047) and mean (0.12) OOB. **(B)** The relative importance of each feature within each level of k. The 13 selected features explain nearly $> 80\%$ of the weight attributed to all of the features.

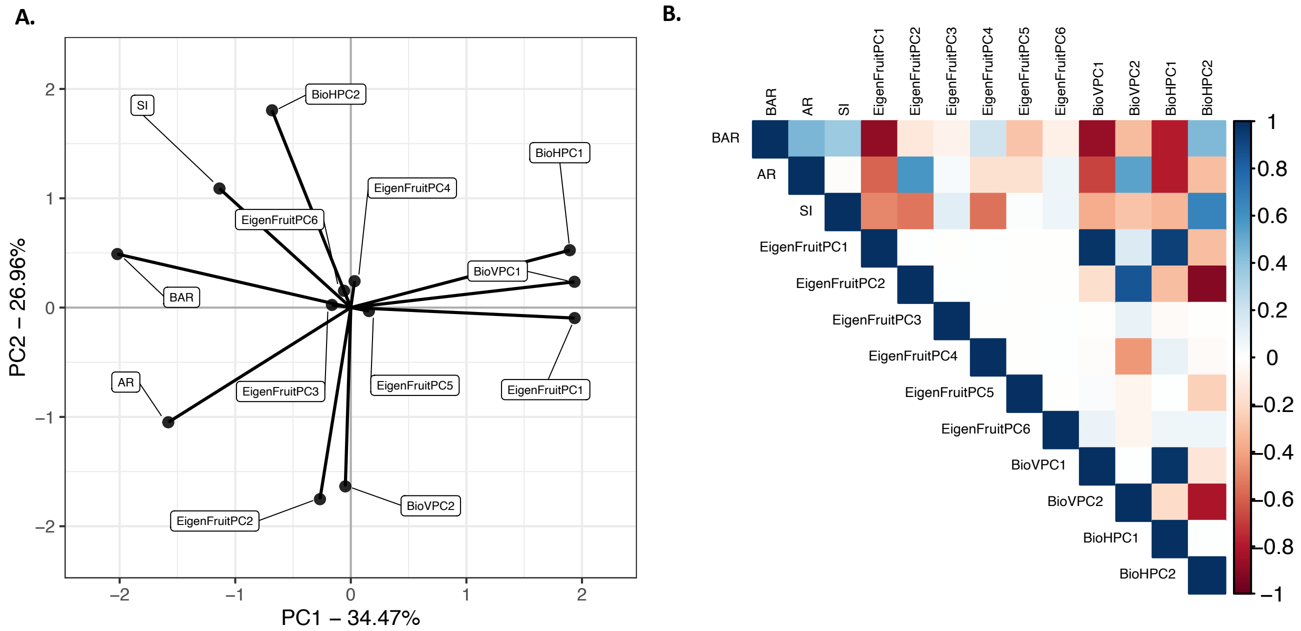

Figure 7. Relationship between selected features. **(A)** Principal directions of the feature variance-covariance matrix among the 13 features selected for classification. **(B)** Pearson correlation matrix of the 13 selected features. Positive correlations are colored blue, negative correlations are colored red.

Table 1. Broad-sense heritability of selected features

Feature	H^2	k Selected	Normalized Eigenvalue $_{(80\%,50\%,20\%)}$	Feature Set
EigenFruit PC1	0.68	9	0.26 (0.27,0.27,0.26)	13, 5, 3
EigenFruit PC2	0.58	8	0.14 (0.14,0.14,0.14)	13, 5
EigenFruit PC3	0.00	3	0.05 (0.06,0.05,0.06)	13
EigenFruit PC4	0.69	5	0.04 (0.04,0.05,0.04)	13
EigenFruit PC5	0.43	4	0.03 (0.03,0.04,0.03)	13
EigenFruit PC6	0.47	5	0.03 (0.03,0.03,0.03)	13
Vertical Biomass Profile PC1	0.67	9	0.65 (0.66,0.66,0.66)	13, 5, 3
Vertical Biomass Profile PC2	0.49	4	0.17 (0.17,0.16,0.17)	13
Horizontal Biomass Profile PC1	0.65	9	0.44 (0.44,0.46,0.44)	13, 5, 3
Horizontal Biomass Profile PC2	0.62	3	0.36 (0.36,0.35,0.37)	13, 5
Bounding Aspect Ratio	0.71	8	*NA*	13
Shape Index	0.72	4	*NA*	13
Ellipse Aspect Ratio	0.58	4	*NA*	13

Broad-sense heritability (H^2) estimated on a per line basis.
k selected is the number of classification models that a feature was selected in, out of 9 (i.e., $k = [2, 10]$).
Normalized eigenvalues is the eigenvalue associated with a specific PC divided by the sum of all eigenvalues.
The large value is the normalized eigenvalue from the full data set. Values in parentheses contain the normalized eigenvalues for the 80%, 50%, and the 20% training sets, respectively.
Feature set indicates in which of the 3 sets a given feature was included.

Table 2. Classification model evaluations validation experiment

Set (Train / Test)	k	H^2	$Accuracy_{13}$	$Precision_{13}$	$Recall_{13}$	FPR_{13}	$Accuracy_5$	$Precision_5$	$Recall_5$	FPR_5	$Accuracy_3$	$Precision_3$	$Recall_3$	FPR_3
80 / 20	2	0.98	0.990 / 0.978	0.990 / 0.978	0.990 / 0.978	0.010 / 0.022	0.995 / 0.982	0.995 / 0.983	0.995 / 0.981	0.005 / 0.019	0.990 / 0.983	0.990 / 0.983	0.990 / 0.985	0.010 / 0.015
	3	0.87	0.985 / 0.963	0.985 / 0.962	0.982 / 0.957	0.009 / 0.019	0.990 / 0.971	0.990 / 0.973	0.989 / 0.969	0.003 / 0.014	0.941 / 0.910	0.938 / 0.906	0.926 / 0.904	0.030 / 0.046
	4	0.85	0.982 / 0.949	0.982 / 0.953	0.981 / 0.943	0.008 / 0.020	0.982 / 0.950	0.983 / 0.952	0.981 / 0.951	0.008 / 0.018	0.946 / 0.921	0.950 / 0.935	0.934 / 0.896	0.019 / 0.029
	5	0.81	0.973 / 0.942	0.979 / 0.949	0.975 / 0.941	0.009 / 0.013	0.976 / 0.955	0.977 / 0.962	0.980 / 0.954	0.008 / 0.010	0.932 / 0.893	0.939 / 0.917	0.928 / 0.879	0.020 / 0.030
	6	0.83	0.973 / 0.943	0.976 / 0.947	0.973 / 0.940	0.008 / 0.010	0.965 / 0.926	0.966 / 0.934	0.965 / 0.919	0.010 / 0.015	0.898 / 0.852	0.903 / 0.876	0.889 / 0.835	0.020 / 0.031
	7	0.83	0.966 / 0.941	0.968 / 0.947	0.966 / 0.940	0.006 / 0.010	0.951 / 0.910	0.952 / 0.922	0.950 / 0.904	0.010 / 0.016	0.870 / 0.824	0.880 / 0.857	0.866 / 0.815	0.021 / 0.030
	8	0.82	0.963 / 0.928	0.964 / 0.934	0.962 / 0.926	0.009 / 0.010	0.866 / 0.825	0.856 / 0.828	0.858 / 0.812	0.018 / 0.027	0.790 / 0.748	0.790 / 0.765	0.778 / 0.731	0.028 / 0.038
	9	0.80	0.954 / 0.920	0.956 / 0.926	0.954 / 0.917	0.009 / 0.010	0.828 / 0.789	0.825 / 0.801	0.827 / 0.781	0.021 / 0.030	0.745 / 0.715	0.751 / 0.736	0.741 / 0.707	0.030 / 0.038
	10	0.81	0.951 / 0.909	0.952 / 0.915	0.951 / 0.906	0.008 / 0.010	0.798 / 0.752	0.798 / 0.770	0.802 / 0.752	0.024 / 0.026	0.708 / 0.679	0.718 / 0.704	0.706 / 0.676	0.034 / 0.036
50 / 50	2	-	0.990 / 0.978	0.990 / 0.978	0.990 / 0.979	0.010 / 0.021	0.993 / 0.983	0.992 / 0.983	0.993 / 0.983	0.007 / 0.017	0.990 / 0.990	0.990 / 0.990	0.990 / 0.990	0.010 / 0.010
	3	-	0.981 / 0.961	0.981 / 0.963	0.980 / 0.958	0.010 / 0.022	0.988 / 0.972	0.989 / 0.974	0.987 / 0.971	0.006 / 0.016	0.943 / 0.907	0.940 / 0.902	0.934 / 0.909	0.030 / 0.047
	4	-	0.979 / 0.951	0.980 / 0.953	0.979 / 0.944	0.010 / 0.019	0.981 / 0.952	0.981 / 0.955	0.980 / 0.954	0.010 / 0.018	0.943 / 0.920	0.947 / 0.933	0.927 / 0.896	0.020 / 0.030
	5	-	0.969 / 0.941	0.972 / 0.945	0.969 / 0.938	0.010 / 0.014	0.969 / 0.948	0.972 / 0.955	0.969 / 0.945	0.010 / 0.012	0.922 / 0.885	0.931 / 0.912	0.916 / 0.869	0.020 / 0.032
	6	-	0.966 / 0.941	0.967 / 0.945	0.966 / 0.939	0.010 / 0.010	0.961 / 0.928	0.961 / 0.935	0.960 / 0.917	0.010 / 0.014	0.887 / 0.856	0.896 / 0.882	0.879 / 0.835	0.022 / 0.030
	7	-	0.961 / 0.934	0.961 / 0.939	0.960 / 0.931	0.010 / 0.010	0.933 / 0.897	0.932 / 0.906	0.931 / 0.887	0.011 / 0.017	0.851 / 0.818	0.861 / 0.848	0.845 / 0.805	0.025 / 0.031
	8	-	0.955 / 0.928	0.957 / 0.931	0.955 / 0.923	0.010 / 0.010	0.872 / 0.831	0.861 / 0.832	0.861 / 0.808	0.017 / 0.027	0.794 / 0.759	0.790 / 0.772	0.776 / 0.738	0.028 / 0.037
	9	-	0.950 / 0.918	0.950 / 0.923	0.949 / 0.910	0.010 / 0.010	0.836 / 0.793	0.830 / 0.799	0.829 / 0.779	0.021 / 0.029	0.746 / 0.718	0.747 / 0.731	0.731 / 0.704	0.030 / 0.038
	10	-	0.947 / 0.909	0.949 / 0.915	0.947 / 0.904	0.010 / 0.010	0.802 / 0.762	0.798 / 0.774	0.804 / 0.755	0.022 / 0.027	0.707 / 0.693	0.716 / 0.713	0.705 / 0.687	0.031 / 0.034
20 / 80	2	-	0.987 / 0.977	0.987 / 0.977	0.986 / 0.977	0.014 / 0.023	0.990 / 0.983	0.990 / 0.983	0.990 / 0.983	0.010 / 0.017	0.990 / 0.986	0.990 / 0.986	0.990 / 0.986	0.010 / 0.014
	3	-	0.973 / 0.955	0.975 / 0.958	0.973 / 0.950	0.013 / 0.024	0.982 / 0.967	0.982 / 0.966	0.981 / 0.967	0.010 / 0.018	0.942 / 0.906	0.943 / 0.907	0.939 / 0.911	0.028 / 0.047
	4	-	0.967 / 0.944	0.971 / 0.951	0.964 / 0.938	0.010 / 0.020	0.973 / 0.953	0.977 / 0.955	0.971 / 0.953	0.010 / 0.017	0.940 / 0.921	0.949 / 0.939	0.921 / 0.892	0.020 / 0.030
	5	-	0.959 / 0.941	0.963 / 0.946	0.954 / 0.936	0.010 / 0.017	0.953 / 0.931	0.954 / 0.939	0.948 / 0.923	0.012 / 0.016	0.899 / 0.875	0.912 / 0.899	0.883 / 0.849	0.026 / 0.033
	6	-	0.953 / 0.935	0.958 / 0.940	0.951 / 0.935	0.010 / 0.012	0.937 / 0.909	0.938 / 0.917	0.935 / 0.899	0.012 / 0.020	0.851 / 0.835	0.864 / 0.861	0.837 / 0.819	0.030 / 0.034
	7	-	0.945 / 0.928	0.950 / 0.933	0.944 / 0.925	0.010 / 0.010	0.902 / 0.876	0.901 / 0.885	0.901 / 0.866	0.016 / 0.021	0.812 / 0.804	0.827 / 0.829	0.799 / 0.789	0.032 / 0.034
	8	-	0.937 / 0.913	0.938 / 0.920	0.933 / 0.914	0.010 / 0.011	0.829 / 0.804	0.825 / 0.810	0.822 / 0.792	0.023 / 0.030	0.736 / 0.733	0.755 / 0.753	0.722 / 0.720	0.039 / 0.040
	9	-	0.930 / 0.908	0.933 / 0.915	0.927 / 0.903	0.010 / 0.010	0.808 / 0.780	0.802 / 0.788	0.798 / 0.767	0.024 / 0.028	0.706 / 0.707	0.724 / 0.727	0.688 / 0.692	0.038 / 0.038
	10	-	0.927 / 0.901	0.930 / 0.905	0.926 / 0.896	0.010 / 0.010	0.794 / 0.758	0.796 / 0.781	0.796 / 0.760	0.023 / 0.028	0.677 / 0.681	0.701 / 0.706	0.670 / 0.676	0.038 / 0.036

Set refers to the 80 / 20, 50 / 50, or 20 / 80 training set / test set split.
k is the number of categories.
H^2 is the broad-sense heritability and was estimated using the full data set.
SVR metric / LDA metric
FPR = False Positive Rate
$_{13}$ refers to a classification model fit with 13 selected features
$_5$ refers to a classification model fit with 5 selected features
$_3$ refers to a classification model fit with 3 selected features

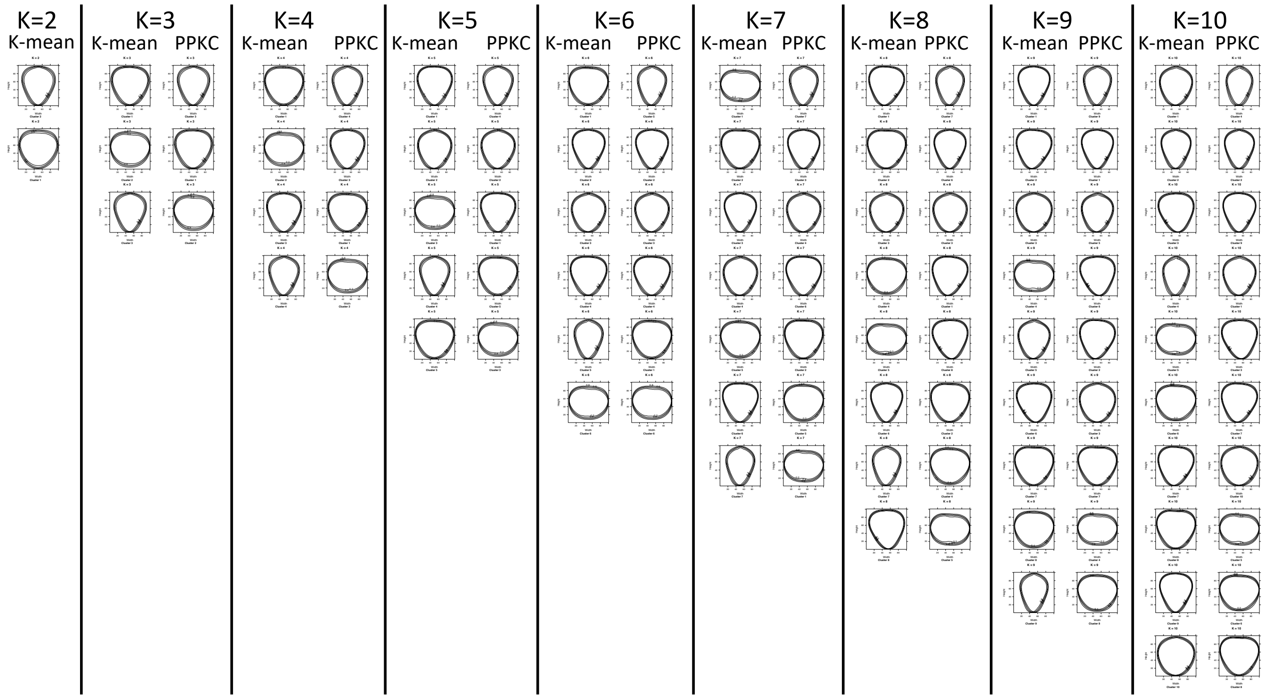

Figure S1. Results of PPKC against original cluster assignments. Ordered centroids from $k = 2$ to $k = 10$. On the left are the unordered assignments from k-means, and the on the right are the order assignments following PPKC.

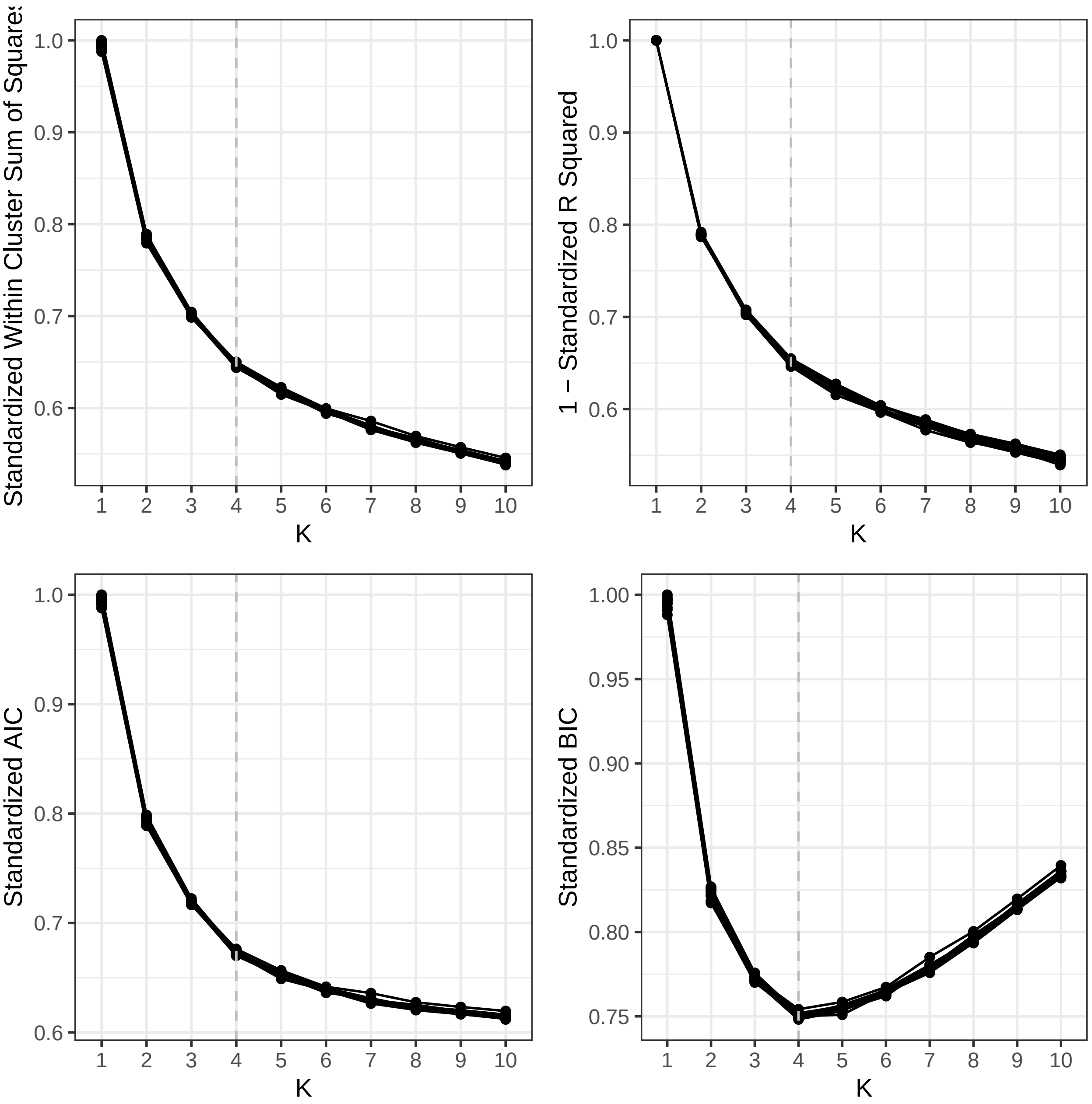

Figure S2. Optimal Value of k. **(A)** Total within cluster sum of squares. **(B)** Inverse of the Adjusted R^2. **(C)** Akaike information criterion (AIC). **(D)** Bayesian information criterion (AIC). All metrics were calculated on a random sample of $3,437$ images (50%). 10 samples were randomly drawn. The vertical dashed line in each plot represents the optimal value of k. Reported metrics are standardized to be between $[0,1]$.

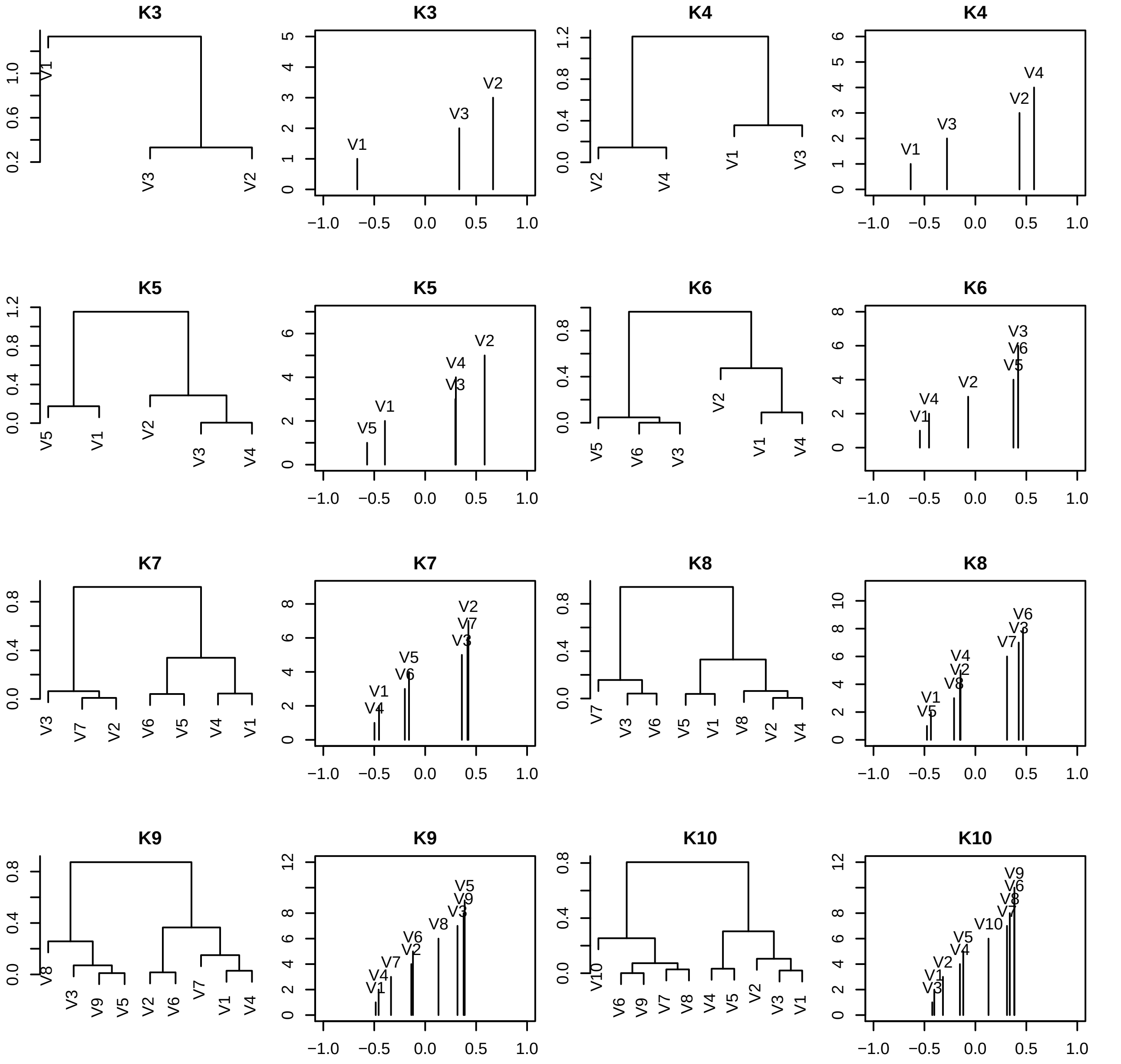

Figure S3. Hierarchical clustering and distance between classes on PC1. The relationship between clusters at each value of k is represented as both a dendrogram and as bar plot. The labels on the dendrogram (i.e., V1, V2, V3,..., V10) represent the original cluster assignment from k-means. The barplot to the right of each dendrogram depicts the elements of the eigenvector associated with the largest eigenvalue form PPKC. The labels above each line represent the original cluster assignment.

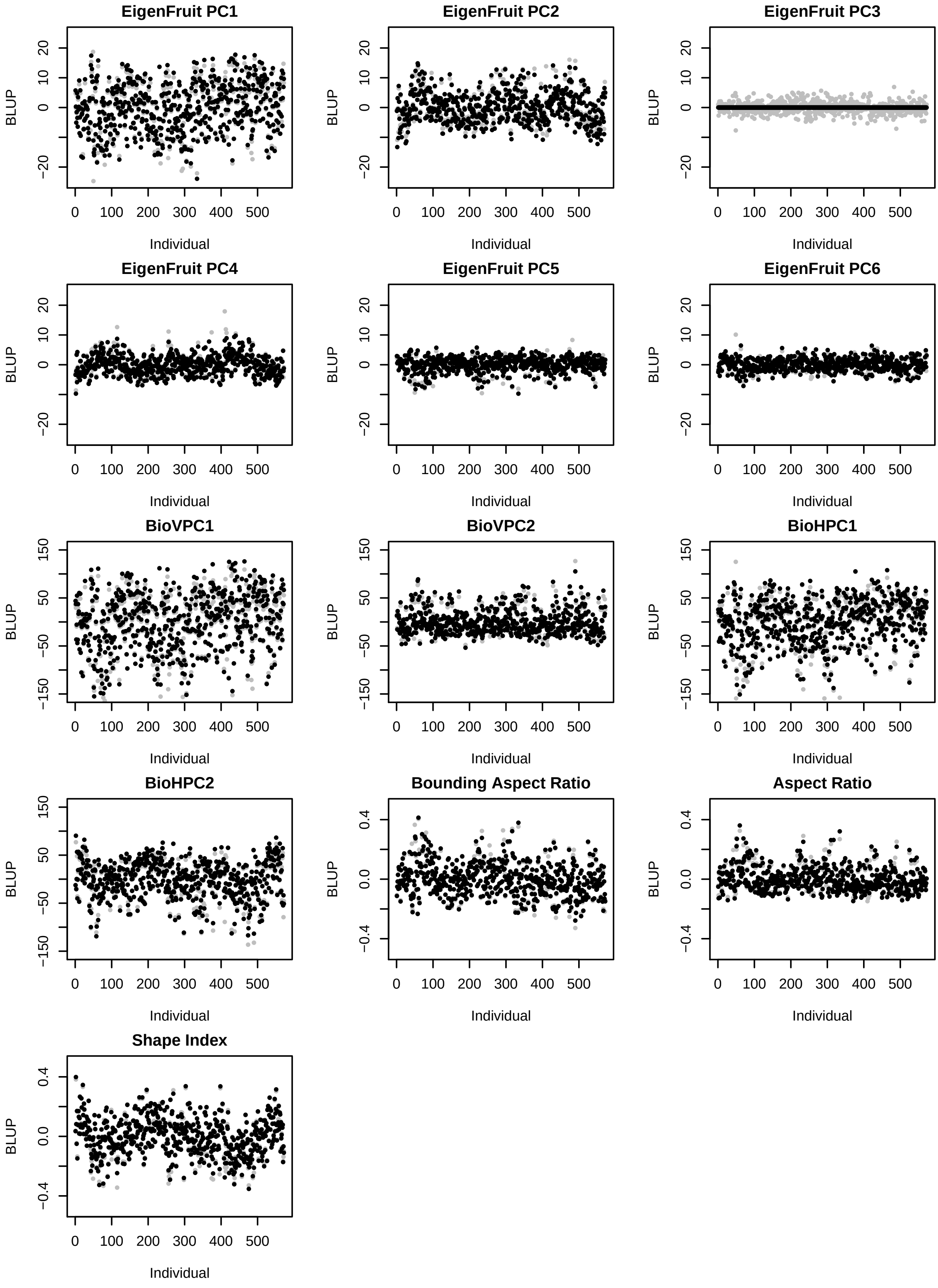

Figure S4. BLUPs for 13 selected features. For each plot, the X-axis is the index and the Y-axis is the BLUP value estimated from a linear mixed model. Grey points represent the mean feature value for each individual. Each point is the BLUP for a single genotype.

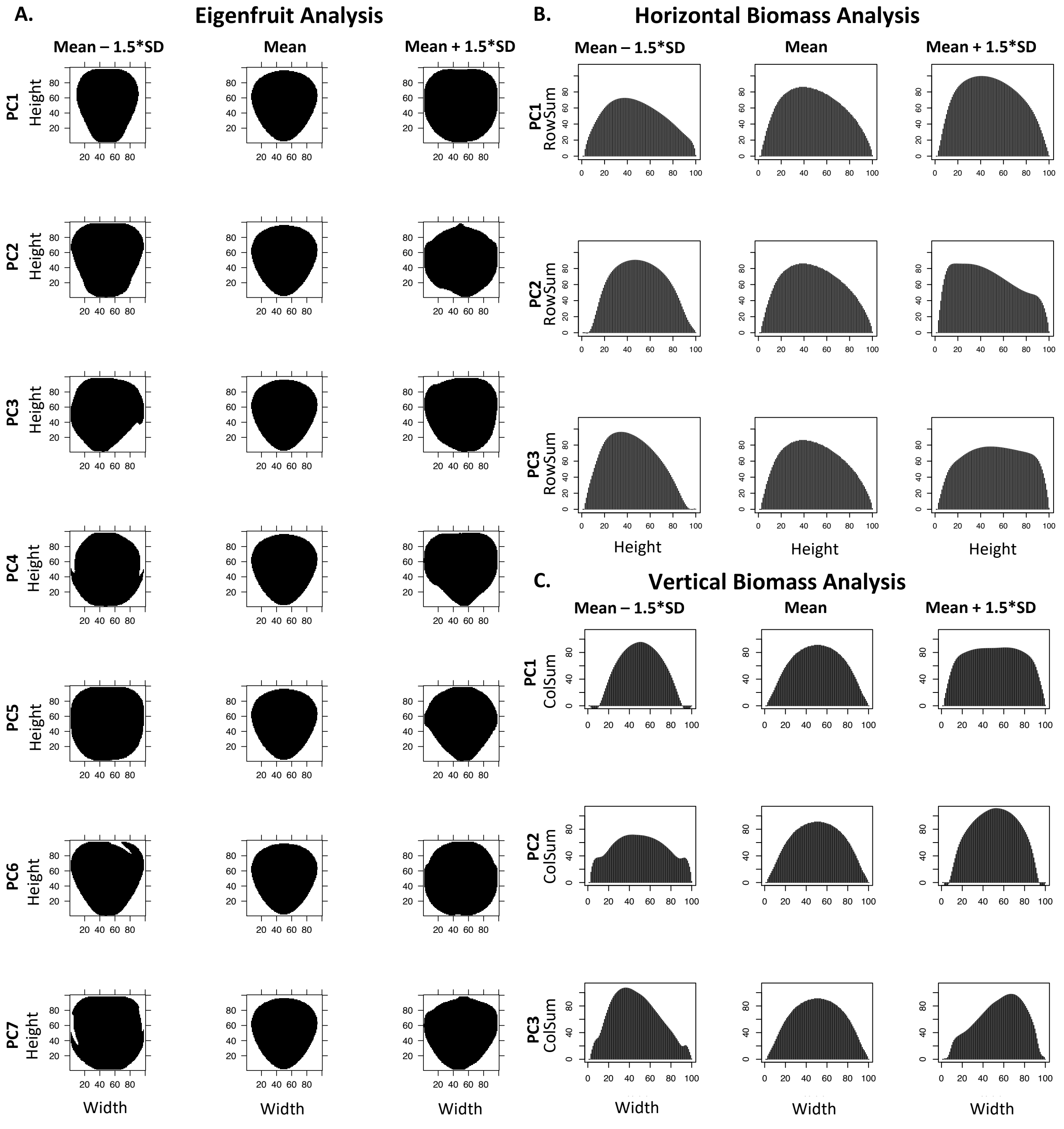

Figure S5. Effects of Eigenfruit, Vertical Biomass, and Horizontal Biomass Analyses. (A) Effects of PC $[1, 7]$ from the Eigenfruit analysis on the mean shape (center column). Left column is the mean shape minus $1.5\times$ the standard deviation. Right is the mean shape plus $1.5\times$ the standard deviation. The horizontal axis is the horizontal pixel position. The vertical axis is the vertical pixel position. **(B)** Effects of PC $[1, 3]$ from the Horizontal Biomass analysis on the mean shape (center column). Left column is the mean shape minus $1.5\times$ the standard deviation. Right is the mean shape plus $1.5\times$ the standard deviation. The horizontal axis is the vertical position from the image (height). The vertical axis is the number of activated pixels (RowSum) at the given veritcal position. **(C)** Effects of PC $[1, 3]$ from the Vertical Biomass analysis on the mean shape (center column). Left column is the mean shape minus $1.5\times$ the standard deviation. Right is the mean shape plus $1.5\times$ the standard deviation. The horizontal axis is the horizontal position from the image (width). The vertical axis is the number of activated pixels (ColSum) at the given horizontal position.

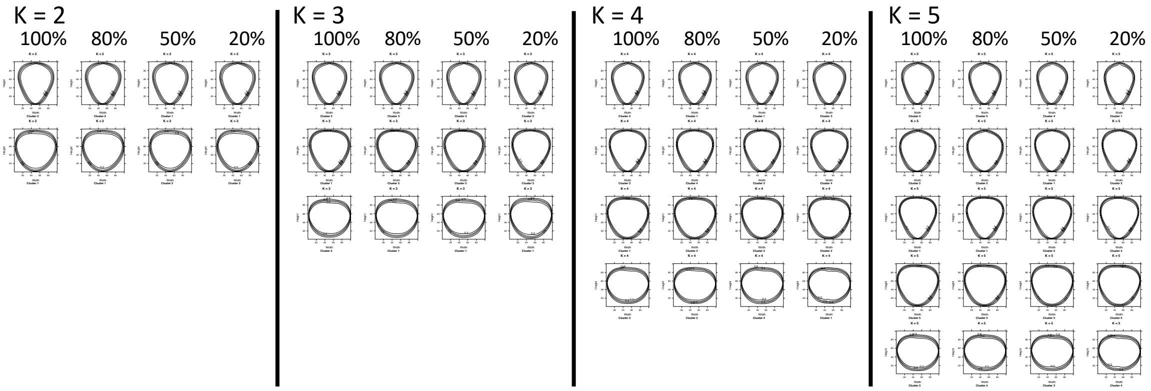

Figure S6. PPKC with variable sample size. Ordered centroids from $k = 2$ to $k = 5$ using different image sets for clustering. For all $k = [2, 5]$, k-means clustering was performed using either 100, 80, 50%, or 20% of the total number of images; $6,874$, $5,500$, $3,437$, and $1,374$ respectively.

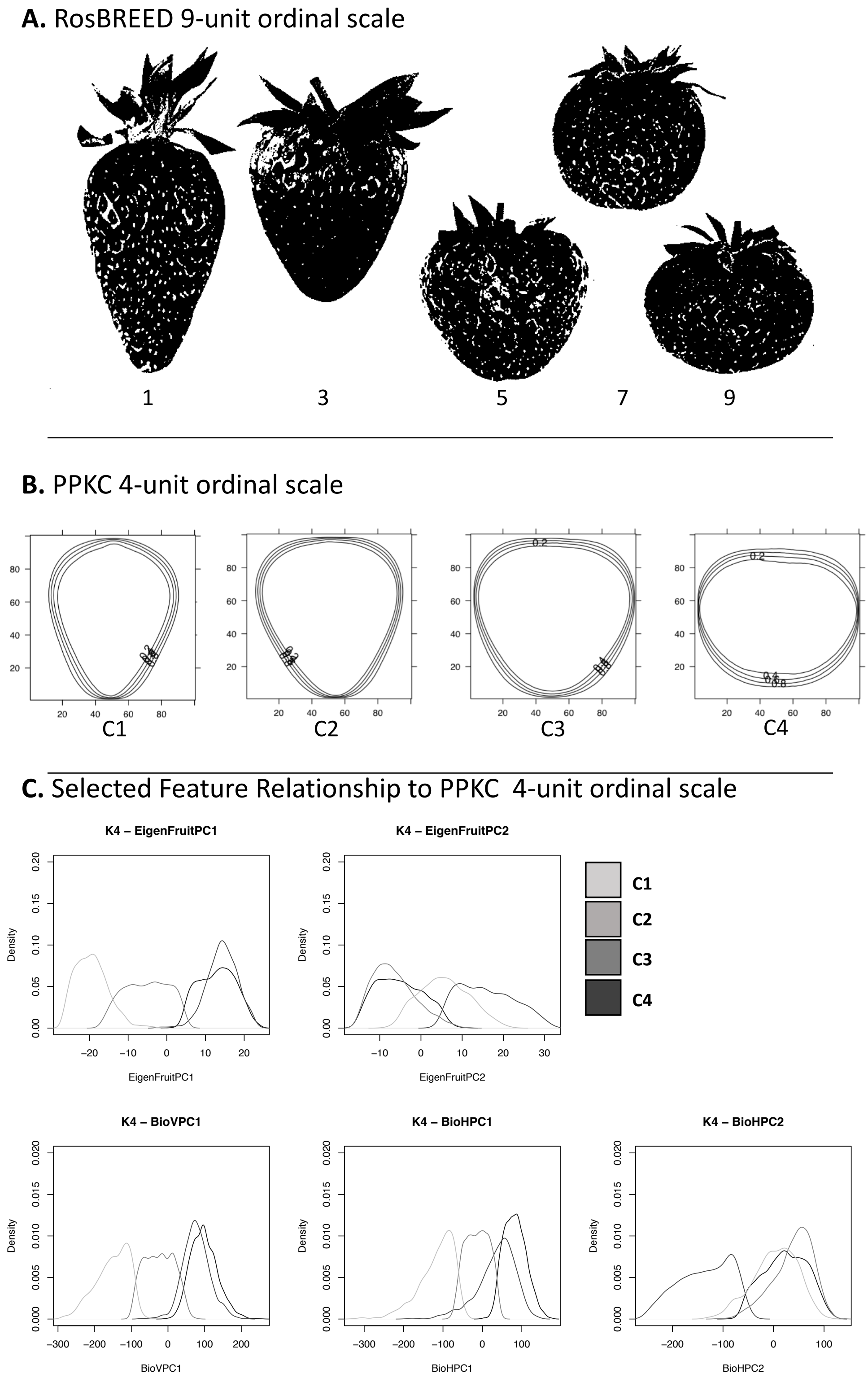

Figure S7. Comparison to RosBREED scale. (A.) RosBREED 9-unit ordinal scale. **(B.)** PPKC 4-unit ordinal scale. **(C.)** Distributions of selected feature with each level of $k = 4$ from the PPKC 4-unit ordinal scale. The light gray line is cluster 1, medium gray line is cluster 2, dark gray line is cluster 3, and black line is cluster 4.
