# Supplementary material for: Multi-dimensional machine learning approaches for fruit shape phenotyping in strawberry
Source: Gigascience. 2020 Apr 30;9(5):giaa030. doi: 10.1093/gigascience/giaa030 (PMC7191992; doi:10.1093/gigascience/giaa030)
Supplement: giaa030_Supplemental_Files [file giaa030_supplemental_files.zip › giaa030_Supplemental_Captions.docx]

**Figure S1. Results of PPKC against original cluster assignments.** Ordered centroids from k = 2 to k = 8. On the left are the unordered assignments from k-means, and the on the right are the order assignments following PPKC. Cluster position indicated on the right [1, 8].

**Figure S2. Optimal Value of k. (A) Total within clusters sum of squares.** (B) The inverse of the Adjusted R . (C) Akaike information criterion (AIC). (D) Bayesian information criterion (AIC). All metrics were calculated on a random sample of 3, 437 images (50%). 10 samples were randomly drawn. The vertical dashed line in each plot represents the optimal value of k. Reported metrics are standardized to be between [0, 1].

**Figure S3. Hierarchical clustering and distance between classes on PC1.** The relationship between clusters at each value of k is represented as both a dendrogram and as bar plot. The labels on the dendrogram (i.e., V1, V2, V3,..., V10) represent the original cluster assignment from k-means. The barplot to the right of each dendrogram depicts the elements of the eigenvector associated with the largest eigenvalue form PPKC. The labels above each line represent the original cluster assignment.

**Figure S4. BLUPs for 13 selected features.** For each plot, the X-axis is the index and the Y-axis is the BLUP value estimated from a linear mixed model. Grey points represent the mean feature value for each individual. Each point is the BLUP for a single genotype.

**Figure S5. Effects of Eigenfruit, Vertical Biomass, and Horizontal Biomass Analyses**. (A) Effects of PC [1, 7] from the Eigenfruit analysis on the mean shape (center column). The left column is the mean shape minus 1.5× the standard deviation. Right is the mean shape plus 1.5× the standard deviation. The horizontal axis is the horizontal pixel position. The vertical axis is the vertical pixel position. (B) Effects of PC [1, 3] from the Horizontal Biomass analysis on the mean shape (center column). The left column is the mean shape minus 1.5× the standard deviation. Right is the mean shape plus 1.5× the standard deviation. The horizontal axis is the vertical position from the image (height). The vertical axis is the number of activated pixels (RowSum) at the given vertical position. (C) Effects of PC [1, 3] from the Vertical Biomass analysis on the mean shape (center column). The left column is the mean shape minus 1.5× the standard deviation. Right is the mean shape plus 1.5× the standard deviation. The horizontal axis is the horizontal position from the image (width). The vertical axis is the number of activated pixels (ColSum) at the given horizontal position.

**Figure S6. PPKC with variable sample size.** Ordered centroids from k = 2 to k = 5 using different image sets for clustering. For all k = [2, 5], k-means clustering was performed using either 100, 80, 50%, or 20% of the total number of images; 6,874, 5, 500, 3, 437, and 1, 374 respectively. Cluster position indicated on the right [1, 5].

**Figure S7. Comparison of scale and continuous features.** (A.) PPKC 4-unit ordinal scale. (B.) Distributions of the selected features with each level of k = 4 from the PPKC 4-unit ordinal scale. The light gray line is cluster 1, the medium gray line is cluster 2, the dark gray line is cluster 3, and the black line is cluster 4.
