# Supplementary figures and images for: Multi-dimensional machine learning approaches for fruit shape phenotyping in strawberry
Source: Gigascience. 2020 Apr 30;9(5):giaa030. doi: 10.1093/gigascience/giaa030 (PMC7191992; doi:10.1093/gigascience/giaa030)

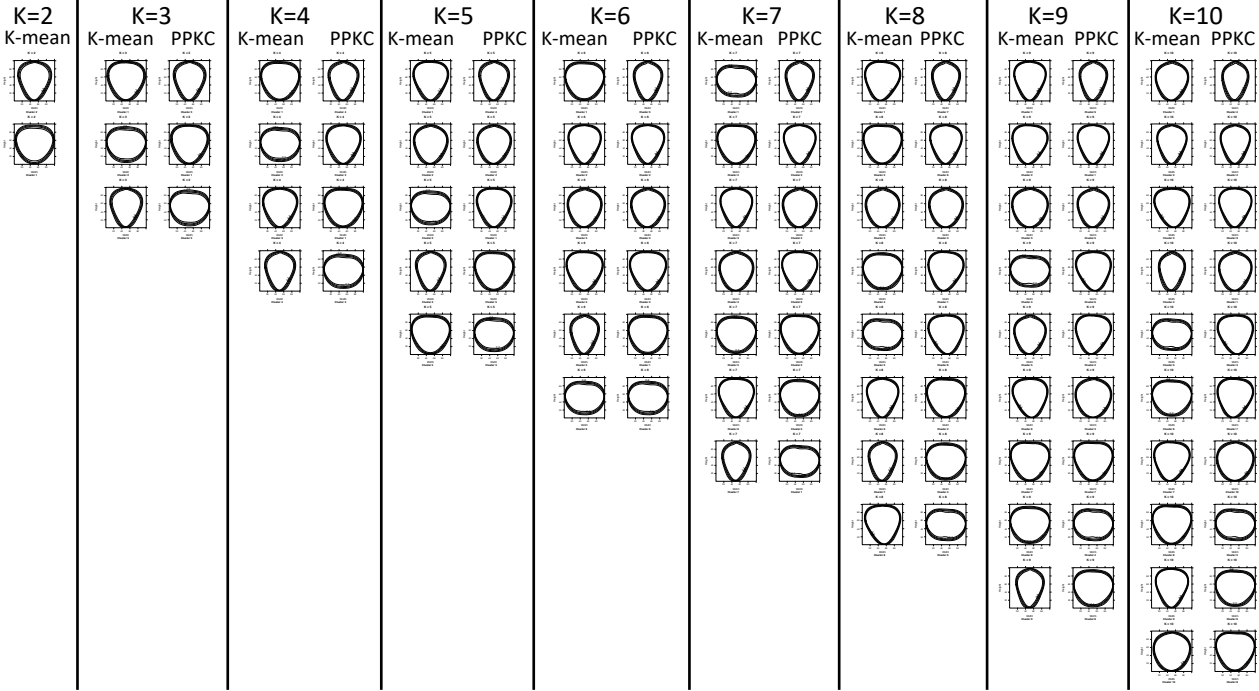

Supplement: giaa030_Supplemental_Files [file giaa030_supplemental_files.zip › S1_Km_vs_PPKC.pdf]

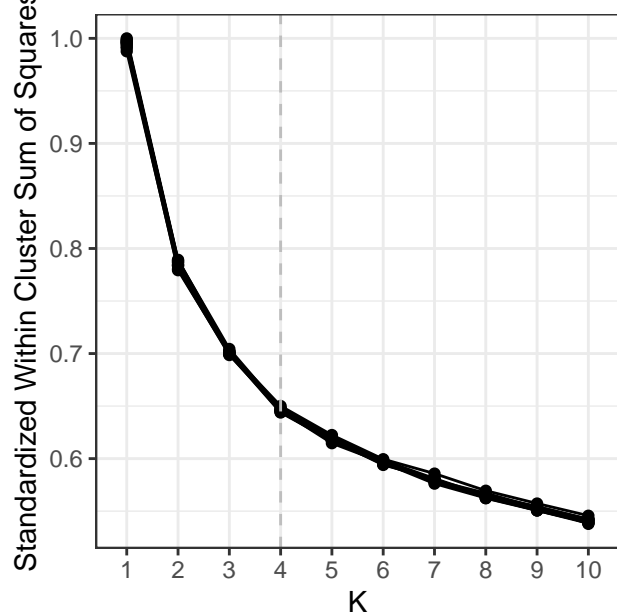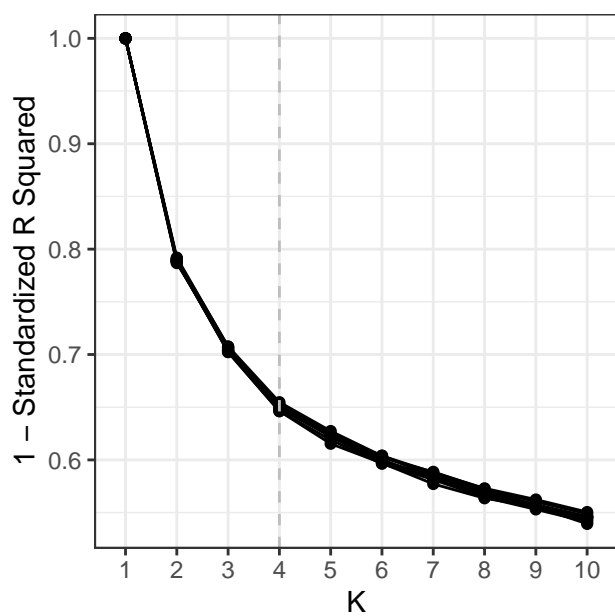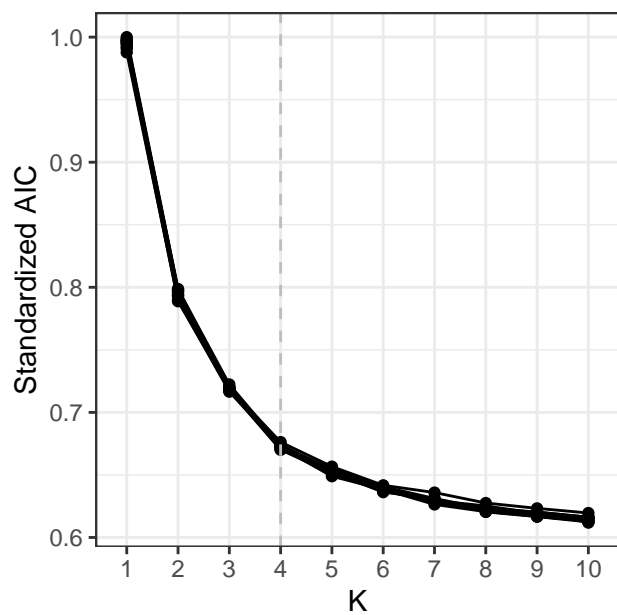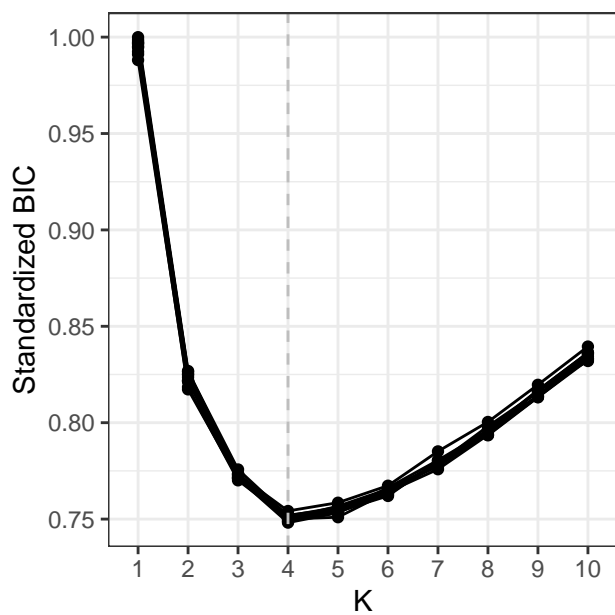

Supplement: giaa030_Supplemental_Files [file giaa030_supplemental_files.zip › S2_optKclust.pdf]

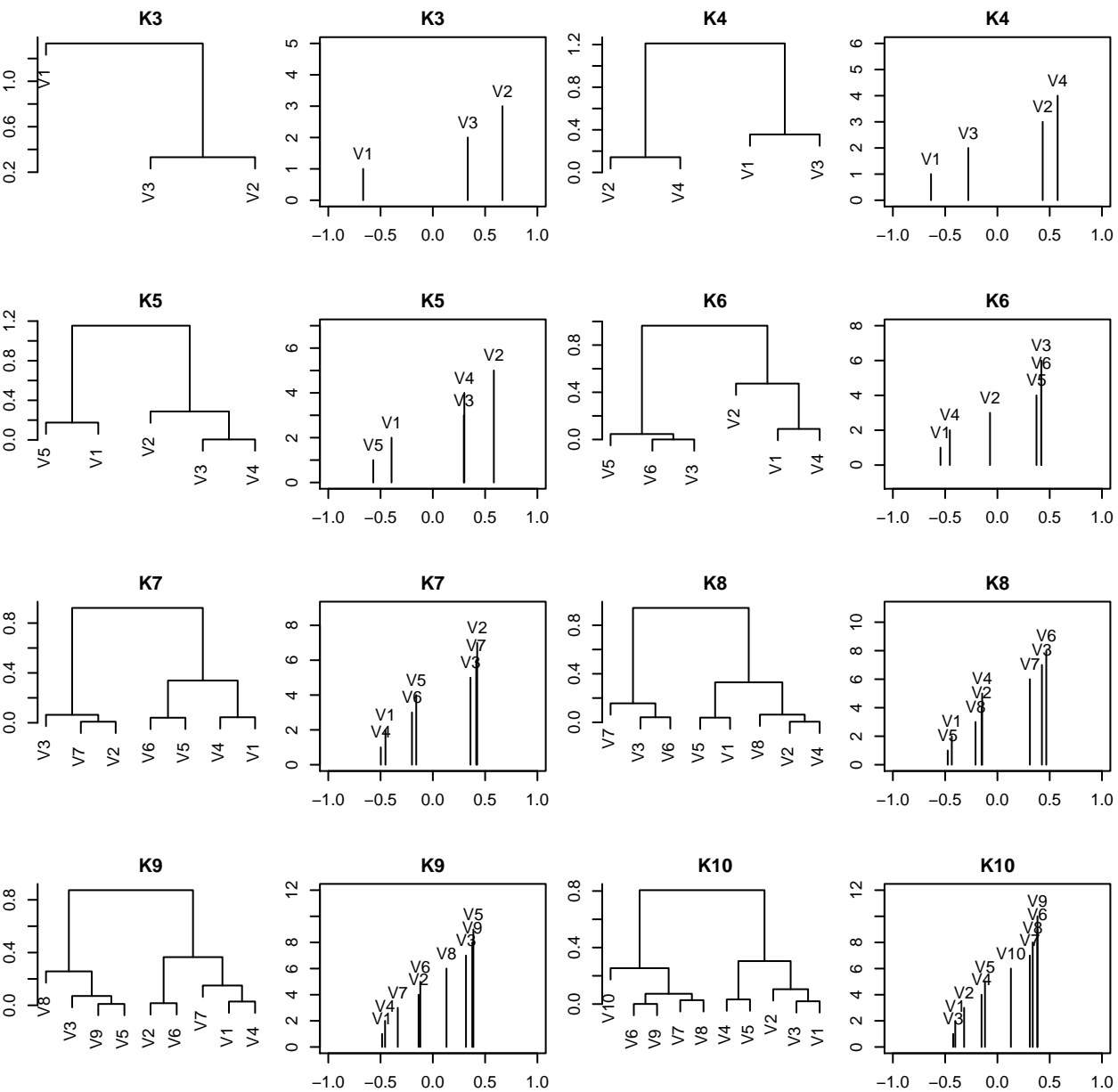

Supplement: giaa030_Supplemental_Files [file giaa030_supplemental_files.zip › S3_PPKC_dend.pdf]

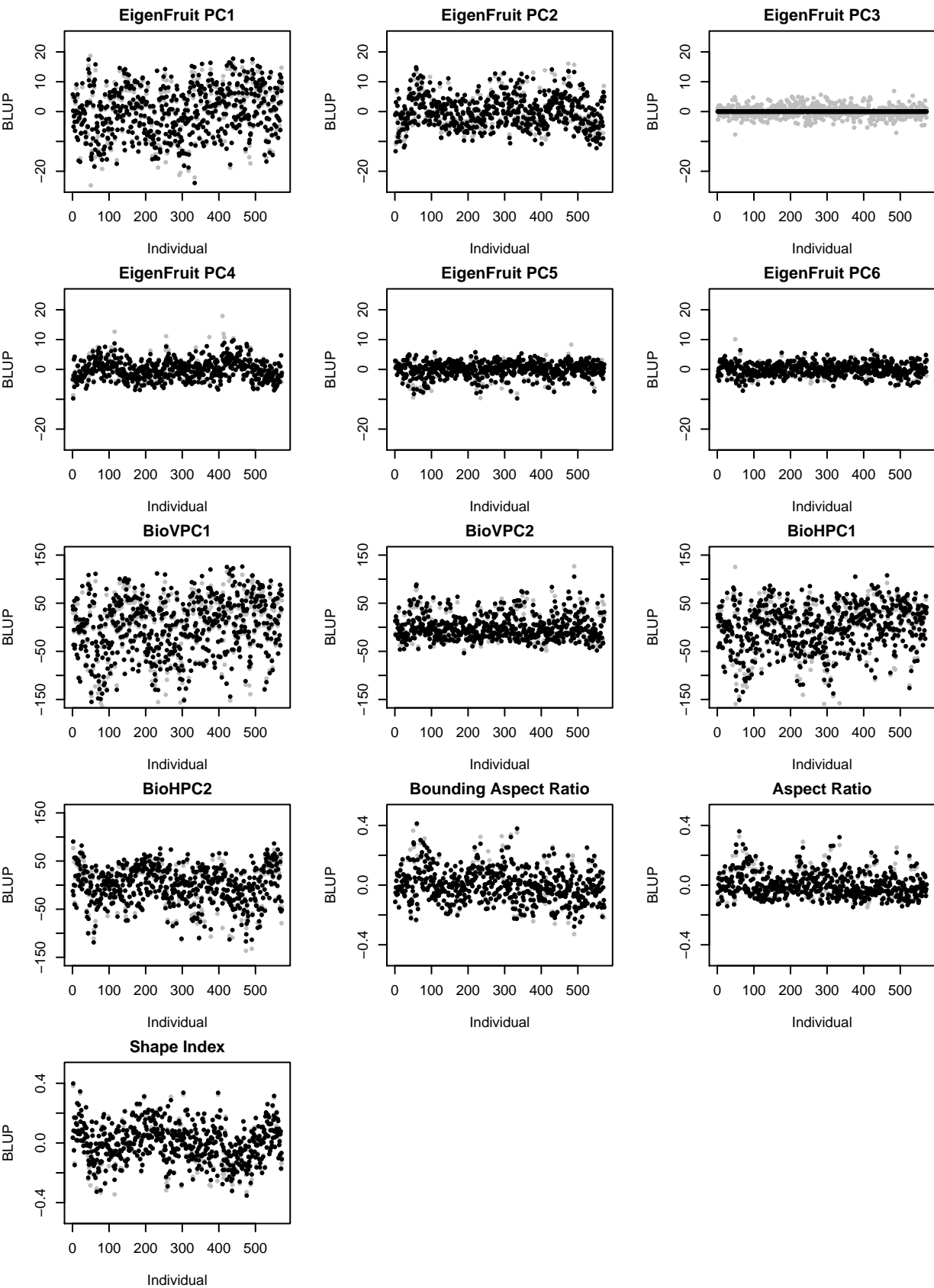

Supplement: giaa030_Supplemental_Files [file giaa030_supplemental_files.zip › S4_BLUP.pdf]

## A. Eigenfruit Analysis

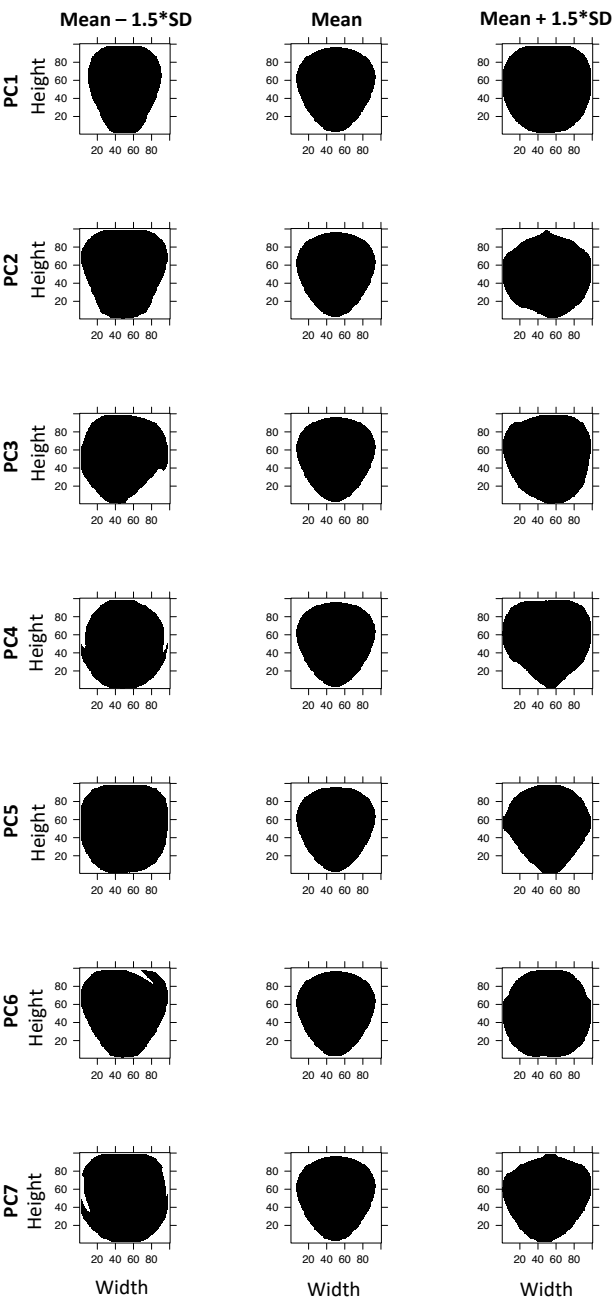

## B. Horizontal Biomass Analysis

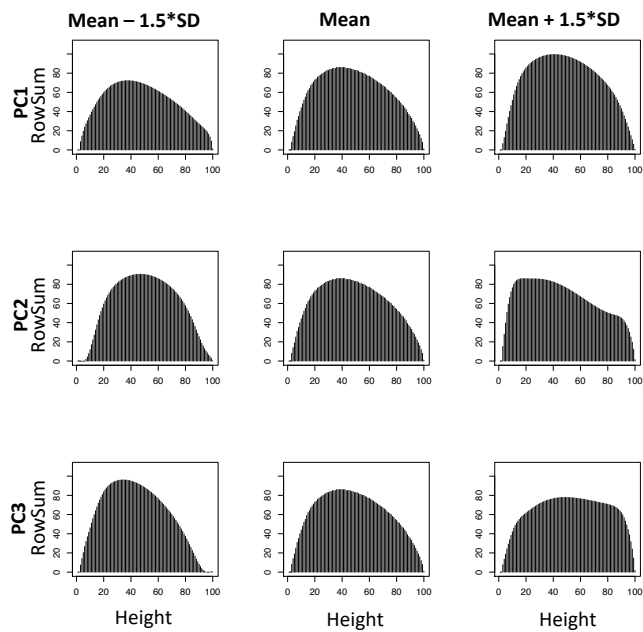

## C. Vertical Biomass Analysis

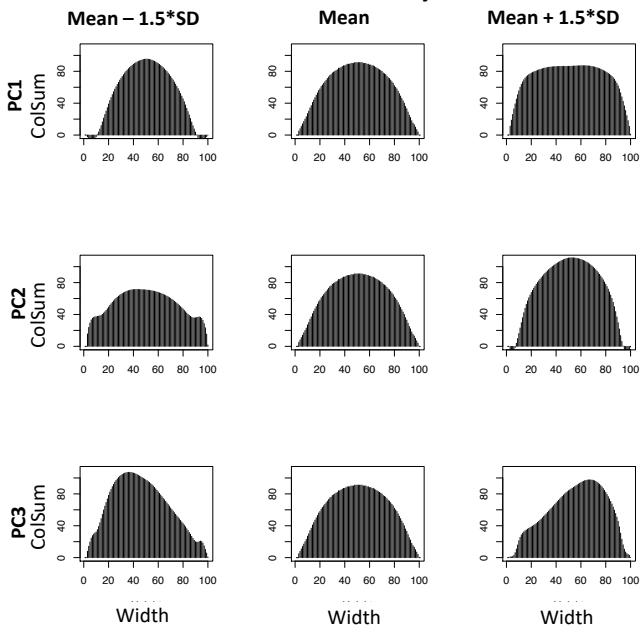

Supplement: giaa030_Supplemental_Files [file giaa030_supplemental_files.zip › S5_PixelBasedFeatures.pdf]

**K = 2**

100%

80%

50%

20%

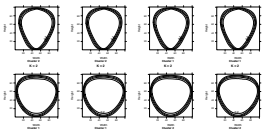**K = 3**

100%

80%

50%

20%

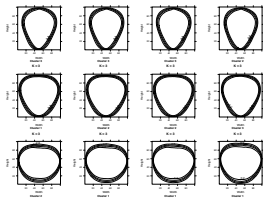**K = 4**

100%

80%

50%

20%

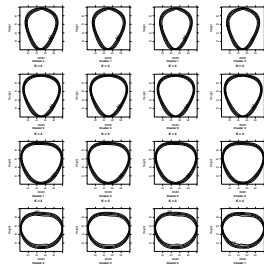**K = 5**

100%

80%

50%

20%

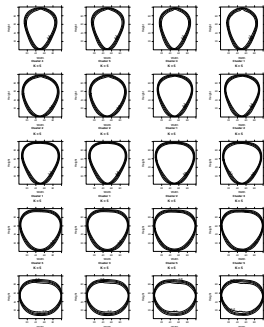

Supplement: giaa030_Supplemental_Files [file giaa030_supplemental_files.zip › S6_ppkcSampleSize.pdf]

## A. PPKC 4-unit ordinal scale

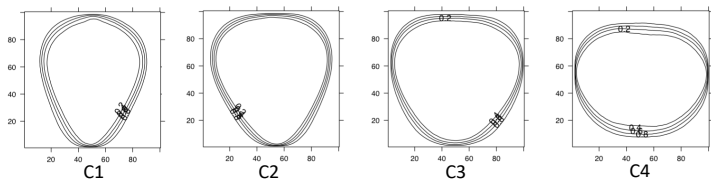

## B. Selected Feature Relationship to PPKC 4-unit ordinal scale

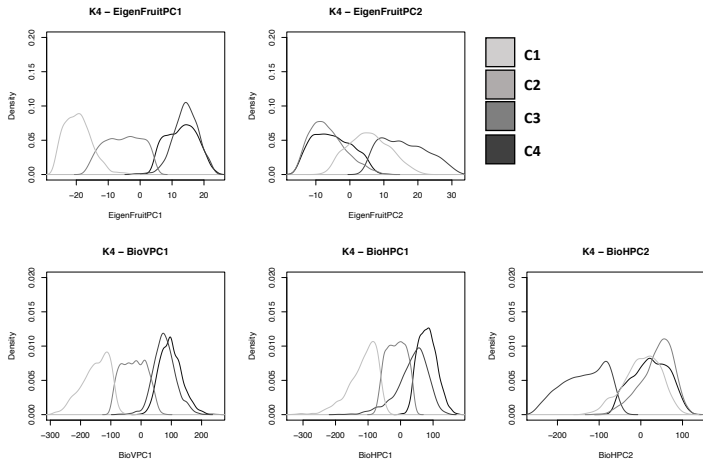

Supplement: giaa030_Supplemental_Files [file giaa030_supplemental_files.zip › S7_4UnitScale.pdf]
