# Supplementary material for: Multi-dimensional machine learning approaches for fruit shape phenotyping in strawberry
Source: Gigascience. 2020 Apr 30;9(5):giaa030. doi: 10.1093/gigascience/giaa030 (PMC7191992; doi:10.1093/gigascience/giaa030)
Supplement: giaa030_Response_to_Reviewer_Comments_Original_Submission [file giaa030_response_to_reviewer_comments_original_submission.pdf]

Author's Response To Reviewer Comments

Close

The revised version of “Multi-Dimensional Machine Learning Approaches for Fruit Shape Phenotyping in Strawberry” has taken into account the concerns of the two reviewers. Point-by-point comments attached below. I have also added an additional supplemental figure (Fig. S8) showing the similarity between the PPKC 4-unit scale and the RosBREED 9-unit scale, alongside the distribution of 5 quantitative features for each of the clusters in the PPKC 4-unit scale in response to Reviewer #1. The majority of changes have taken place in the introduction and discussion sections. I have added 1 new main figure (Fig. 2) visualizing the analysis workflow, as suggested by Reviewer #2.

Reviewer #1:
There really is not a major focus on recognition of varieties in the manuscript. I suggest removing "recognition" from the title given the focus on quantitative genetic application, or better highlight the use of these data for recognition.
The primary focus of this paper was not to recognize strawberry cultivars, but to recognize and label the shapes that as a phenotyping method. I can see why this might be a little misleading and I removed “Recognition and” from the title. The point is phenotyping for future genetic applications, although cultivar description and recognition could be another future application.

Suggest changing "genome-wide and forward genetic analyses" to "genetic dissection and prediction analyses". Also, there is some slight clarification needed in the manuscript. GWAS is typically considered a reverse genetics method going from genotype (gene) to phenotype, while linkage analysis (QTL analysis) is considered forward genetics going from phenotype to genotype (gene). I suggest just dropping the repeated reference to forward genetic analyses and change to quantitative genetic analyses.
The revised draft takes this into account throughout.

I understand the importance of nominal vs. ordinal, but continuous (e.g., 2.3, 5.7., 8.9 etc.) distributions are more favorable compared to ordinal ranks (e.g., 1, 2, 3...10) when mapping phenotypes. Continuous affords higher statistical power when dissecting the genetic architecture of traits. Perhaps this is an issue of semantics but it still needs exactness in the writing.
This is absolutely correct. In this manuscript, we suggest using the quantitative measurements that we extract from the images, and not the ordinal categories themselves for genetic studies. Although, we hypothesize that it would reasonable to use the ordinal categories in a breeding situation where decisions need to be made quickly, for automated sorting, or for bulk segregant type experiments..

In the Introduction, it is stated that "genetic determinants of fruit shape are unknown in strawberry" but then the next sentence cites two papers that presumably have "uncover some of the genetic basis of fruit shape in…strawberry." This needs resolving in a revision.
The specific research that implicates genetic mechanism was done in diploid strawberry [citation 9] and [citation 42] implicates 5 QTL influencing fruit shape (=Width / Length) in a single biparental cross. [citation 42] does estimate h^2=0.72 (in our study H^2=0.71 for the equivalent trait [BAR]), but they do not describe any amount of PVE for any of the 5 QTL that they implicate. So, while they do discover potential QTL, they do not report effect sizes or any actionable information regarding these QTL. However, I do see how this is potentially misleading and confusing and have adjusted the wording to “However, the major genetic determinants of fruit shape remain unclear or understudied in octoploid strawberry” [line 63].

There are instances of where the Results section has material that is best left for the Discussion. As an example, there is a discussion of the underlying cause of heritability = 0 for a feature that is needed but best included in the Discussion. The Discussion is really missing the comparative of this method to other related digital phenotyping and associated statistical approaches for fruit shape phenotyping. Having the inclusion of this content is truly needed to put the presented work into its proper context.
The second paragraph of the discussion is new and discusses phenotyping through latent variables (PCA, MDS, auto-encoders, and SEM) and the challenges of interpreting these latent features. Following this is

the discussion of H2 = 0. This paragraph has undergone fairly heavy modification and discusses several studies where latent variables are used as phenotypes where heritability is estimated.

Equation 9. This is typically harmonic mean of observed plots for a genotype and not blocks. I suggest looking at Jim Holland's papers on how to estimate broad-sense heritability. I may have misinterpreted your design, however.
The quantity hb in equation 9 is equivalent. Each individual is replicated once per block (b) on two harvest dates (h). So r_G=hb in this case. I have modified the notation to be hr instead of hb.

Stratified sampling was not needed for CV to ensure that each fold was representative of family (75 biparental crosses) composition in the entire population?
Certainly, stratified sampling would be necessary to ensure that each family was represented in each fold. However, our objective was to assess categorization of shape categories of individual fruit regardless of pedigree. In our experiment, we were concerned about missing categories, but even when there were 10 categories and 20% of the data used for training, we didn't seem to run into any issues with missing clusters. I have included a statement [line 310] to mention this to point out that the 10 iterations that we performed may have been lucky to incorporate all 10 groups and that if we had far fewer images and highly unequal samples per cluster, stratification would have been necessary.

It would be interested to know for at least a small subset of how these quantitative estimates relate to visual ratings historically used in strawberry breeding programs.
I have added a new supplemental figure (Fig. S8) to try to account for this and have referenced it in the discussion. Fig. S8 shows images of the RosBREED 9-unit scale, PPKC 4-unit scale, and distributions of 5 of the selected variables for each of the categories in the PPKC 4-unit scale. I chose the 5 selected feature set, to reduce the size of the Fig. S8, and because the classification accuracy with these 5 features was roughly equivalent to the feature set containing 15 features. I did not explicitly estimate the parameters for the RosBREED shapes, but the visual similarity between the our PPKC scale and the RosBREED scale, should be indicative of the relationship of the quantitative estimate and the RosBREED scale.

Reviewer #2:
The main concern I am having is about the Modified k-clustering (line 115-149) which is also the very early step of the analysis. The authors observed there are two groups (c2 and c6) are mirror symmetric when they performed k (=8)-mean clustering. Thus they rotated the images in one of these two groups and redo the k-mean clustering. In fact, if we "observe an object from two opposite sides, they would appear as mirror images of one another". But this is true for all the images, not just the one in group c6. And the original dataset includes the random factor. Assume there is a direction reflecting the mirror symmetry. Only rotating the images in c6 means only move the most extreme data points into the other most extreme side. This will result in partially reducing the variance caused by the mirror symmetry. However, it will also change the whole data structure and pairwise distance. For most empirical data, it is very common to have relatively continuous distribution. I believe it is also the case for this strawberry data. For different k, the samples that closer to group c6 might be grouped together with some of the samples in c6. But after partially rotate c6, those samples will be far from them. I am not sure whether it is valid to do it this way. What if PPKC is performed on the original dataset? Will the result be very different? I think it is more careful to eliminate the mirror symmetry for the whole dataset not just partially, or just using the original dataset without stratification.
Our concern was that at k = 8 two of the categories were visually similar but mirror images of one another. I will note that this was determined with prior to assessing any goodness-of-fit measures or heritability. There was typically symmetry to most of the discovered categories. However, when k=8 but there was a clear visual asymmetry in these two categories. Effectively, the original k8_{c2} and k8_{c6} were both asymmetrically lobed, one to the left and one to the right (Fig. S1B). It seems likely that this could have arisen from difficulties in choosing which perspective to image the fruit and not from any biologically relevant feature. One challenge that we recognize with 2D phenotypes in strawberry, is the lack of an obvious, homologous perspective for imaging. Because the objects are somewhat symmetric, we determined that imaging the widest fruit face would be the standard and would likely get us to the closest thing to a biologically homologous view. However, a 180º rotation from the widest face, is again the widest face. So, while the data structure would change, we feel that these changes are for the best and results in more visually, distinct categories. Furthermore, the results from PPKC are visually identical when k = [2,7]. Only after k=8, do the PPKC results differ between the two approaches.

I do have a few questions about the new methods PPKC.
With the context and math equations, I understand the method itself such as the math and the

algorithm. But I still had hard time to interpret and understand how it works. If I understand correctly, the PCA treats the k clusters as variables and each former groups as the observations. The PC1 loadings calculate the contributions of these k clusters to the most variance. But I am still not sure how it can reflect the progression categories. I think it is even challenged for the readers who may not be familiar with PCA. Thus, I think, beside those math theory, it would be better to provide more intuitive interpretation.
I have included a statement regarding this in the results section [line 206-211]. "In theory, this perspective of covariance should be able to separate the classes effectively as it describes a linear axis containing the greatest amounts of independent variation. The value a category takes on this composite axis is therefore suggestive of its linear relationship to other the k categories being considered."

The reason why the PPKC in k=8 did not follow the same pattern is not convinced me as I didn't see the "dramatic change of slop in the total within-group sums of squares, AIC, and Adjusted R2" (line 206-208) at k=8. Is the k going to be different number for different dataset? Is the method not stable enough?
Yes, we believe that this is a function of the specific data set. The model is highly overfit when k = 8 (Fig. S3) and so we are not specifically concerned with the order at this level. If we had a data set that had better fit at higher levels of k (more unique categories from a likely more diverse data population), we believe that this method would continue to perform as expected as k increases. We estimate the order for 5 < k < 10 and show their order, but for future analyses we would only use k = 4. If we had more data from a more diverse background, we hypothesize that the optimal value of k would be different and that the ability to order larger values of k would improve.

For a new method, the readers usually would like to know whether there are other methods have been developed for the similar purpose. What are the advantage and limitation of this new approach. (Line 39-41) Is it the first method that is able to provide ordinal categories? If not, the comparison with some previous method is commonly asked.
As far as I am able to determine, this is the first method specific for transforming a multinomial scale into an ordinal scale. One of the things we were interested in when developing PPKC, was to be able to rapidly collect large amount of data (images) and categorize them as with a machine. Ordinal scales are common in strawberry and other crops for various traits, but they are typically arbitrarily defined a priori. This method is advantageous because it reduces cost, bias, and the need for arbitrary a priori decisions [line 47]. We specifically state [line 211-213], "However, we note that relationships containing branches, bubbles, and other topological features will likely not be captured accurately."
Like exploratory "ordination" techniques used in community ecology and other fields, PPKC does rely on the eigenvalue decomposition to generate the ranked order. Conceptually, the PCA step of PPKC could be replaced by multi-dimensional scaling (metric or non-metric), correspondence analysis, or other such techniques. The reason we chose PCA is the repeatability of the discovered axes (i.e., there is one solution given an input) unlike MDS and t-SNE which are either dependent one the starting conditions or stochastic. I incorporated a statement about this in the discussion [line 404-408].

Minor points:
Line 23-24, this sentence is same with the one in the abstract. Please rephrase either of them.
corrected

The sentences from line 86-98 provides the outline of this work. But maybe because there are many different steps with many details to process the images, extract features, machine learning, clustering and classification, readers may get lost. I think a workflow for the entire analysis from input raw data to the output analysis result with the details will better guide the readers.
Added a new figure in response. The new figure is a flow chart which details the analytical workflow. I have indicated these figures on [line 89] and elsewhere in the manuscript.

Line 136 and 138, there is no Fig. S1D and Fig. S1E in the figure.
corrected

Line 158, AIC and BIC are abbreviation. I think it would be better to give the full phrase when they are mentioned in the first place.
corrected

Line 171, just curious how to compute the phenotypic variance for the ordinal scales.
The purpose of this statement at 171 is more of a visual exercise than a theoretical one. Imagine having two 100-element vectors of integers [1,…,5]. If I "re-order" these integers as [4,2,1,5,3] the resulting

histogram has a different “shape”. If we then assume that there is some underlying continuous distribution, the probability of an observation being above or below some arbitrary threshold (the quartiles for instance) changes. However, I agree that this may be a bit confusing for other readers and have removed this statement. I replaced it with “as the relative relationship between the k categories is identical in both (e.g., c3 is more related to c2 than either c1 or c4).” [line 177 - 182]

Equation (1), the denominators of the last row are typos.
corrected

Line 239, same comment for OOB as minor point 3.
corrected

Line 610, same comment for PVE as minor point 3.
corrected

What is the computational time?
Because we only interact with relatively small covariance matrices for PPKC, the computational time for this algorithm is <0.15s on a 2015 MBP. K-means clustering is much more computationally expensive. Included a statement [line 222-225].

Close
