# Supplementary material for: Multi-dimensional machine learning approaches for fruit shape phenotyping in strawberry
Source: Gigascience. 2020 Apr 30;9(5):giaa030. doi: 10.1093/gigascience/giaa030 (PMC7191992; doi:10.1093/gigascience/giaa030)
Supplement: giaa030_Response_to_Reviewer_Comments_Revision_1 [file giaa030_response_to_reviewer_comments_revision_1.pdf]

# Author's Response To Reviewer Comments

Close

I would like the thank the reviewer for their critical consideration of this work. The reviewers main concern is the the modified k-clustering approach.

The reviewer suggested clustering larger values of k from 10 to 20 and interrogating whether or not other mirrored categories appear to test if this solves this problem globally. However, I do not believe that this would globally test the appropriateness of this modification. While, I do think that an intelligent, programmatic method could be developed to globally test for mirror symmetries in this type of data, I do not know of such a test. Therefore, I opted to redo the analyses in this manuscript excluding the modification to the k-means clustering and retaining the random effect of orientation in the data.

I went to the original images and clustered using k-means for k = [2,10], performed PPKC, extracted features, estimated H2, and performed the image classification as before. Few minor elements in this manuscript have changed with these edits. (1) The overall prolate to oblate order is recovered with PPKC, (2) the same optimal value of k (k=4), (3) Heritabilities of the ordinal scales are very similar (H2=0.8+), (4) the selected variables are very similar (primarily consisting pixel-based descriptors) and (5) the accuracy of classification was also high for the different training/test set combinations. Removing the modified k-means approach from the strategy did not change the main results of this manuscript indicating that the modification to k-clustering was likely unnecessary to begin with.

I believe that the interrogation of the modification to k-means has made this article more accessible, more reproducible, and removes a potential source of confusion for readers as well as a source of bias in these analysis.

Close
