# Supplementary material for: Multi-dimensional machine learning approaches for fruit shape phenotyping in strawberry
Source: Gigascience. 2020 Apr 30;9(5):giaa030. doi: 10.1093/gigascience/giaa030 (PMC7191992; doi:10.1093/gigascience/giaa030)
Supplement: giaa030_Response_to_Reviewer_Comments_Revision_2 [file giaa030_response_to_reviewer_comments_revision_2.pdf]

Author's Response To Reviewer Comments

Close

We have added the citation to the Gigascience Database and have added statements in the sections "Data Description" and "Availability of Supporting Data and Materials" citing this repository alongside the Zenodo repository and the Github page. Because we added a similar statement citing GigaDB in the "Data Description" section, the additional citation is [68].

I have been in communications with Chris to finalize the GigaDB repository associated with this manuscript, and will approve that database as soon as it is transferred to me.

Close
