# Supplementary material for: Multi-dimensional machine learning approaches for fruit shape phenotyping in strawberry
Source: Gigascience. 2020 Apr 30;9(5):giaa030. doi: 10.1093/gigascience/giaa030 (PMC7191992; doi:10.1093/gigascience/giaa030)
Supplement: giaa030_Reviewer_1_Report_Original_Submission — Michael Gore -- 9/14/2019 Reviewed [file giaa030_reviewer_1_report_original_submission.pdf]

**Reviewer Report**

**Title: Multi-Dimensional Machine Learning Approaches for Fruit Shape Phenotyping in Strawberry**

**Version: Original Submission** **Date:** 9/14/2019

**Reviewer name: Michael Gore**

**Reviewer Comments to Author:**

I have reviewed the manuscript of Feldmann et al. on shape recognition and phenotyping of strawberry I very much enjoyed reading this manuscript.

There really is not a major focus on recognition of varieties in the manuscript. I suggest removing "recognition" from the title given the focus on quantitative genetic application, or better highlight the use of these data for recognition.

Suggest changing "genome-wide and forward genetic analyses" to "genetic dissection and prediction analyses"

Also, there is some slight clarification needed in the manuscript. GWAS is typically considered a reverse genetics method going from genotype (gene) to phenotype, while linkage analysis (QTL analysis) is considered forward genetics going from phenotype to genotype (gene). I suggest just dropping the repeated reference to forward genetic analyses and change to quantitative genetic analyses.

I understand the importance of nominal vs. ordinal, but continuous (e.g., 2.3, 5.7., 8.9 etc.) distributions are more favorable compared to ordinal ranks (e.g., 1, 2, 3...10) when mapping phenotypes. Continuous affords higher statistical power when dissecting the genetic architecture of traits. Perhaps this is an issue of semantics but it still needs exactness in the writing.

In the Introduction, it is stated that "genetic determinants of fruit shape are unknown in strawberry" but then the next sentence cites two papers that presumably have "uncover some of the genetic basis of fruit shape in…strawberry." This needs resolving in a revision.

There are instances of where the Results section has material that is best left for the Discussion. As an example, there is a discussion of the underlying cause of heritability = 0 for a feature that is needed but best included in the Discussion.

The Discussion is really missing the comparative of this method to other related digital phenotyping and associated statistical approaches for fruit shape phenotyping. Having the inclusion of this content is truly needed to put the presented work into its proper context.

Equation 9. This is typically harmonic mean of observed plots for a genotype and not blocks. I suggest looking at Jim Holland's papers on how to estimate broad-sense heritability. I may have misinterpreted your design, however.

Stratified sampling was not needed for CV to ensure that each fold was representative of family (75 biparental crosses) composition in the entire population?

It would be interested to know for at least a small subset of how these quantitative estimates relate to visual ratings historically used in strawberry breeding programs.

**Methods**

Are the methods appropriate to the aims of the study, are they well described, and are necessary controls included? Choose an item.

**Conclusions**

Are the conclusions adequately supported by the data shown? Choose an item.

**Reporting Standards**

Does the manuscript adhere to the journal's guidelines on minimum standards of reporting? Choose an item.

Choose an item.

**Statistics**

Are you able to assess all statistics in the manuscript, including the appropriateness of statistical tests used? Choose an item.

**Quality of Written English**

Please indicate the quality of language in the manuscript: Choose an item.

**Declaration of Competing Interests**

Please complete a declaration of competing interests, considering the following questions:

- Have you in the past five years received reimbursements, fees, funding, or salary from an organisation that may in any way gain or lose financially from the publication of this manuscript, either now or in the future?
- Do you hold any stocks or shares in an organisation that may in any way gain or lose financially from the publication of this manuscript, either now or in the future?
- Do you hold or are you currently applying for any patents relating to the content of the manuscript?
- Have you received reimbursements, fees, funding, or salary from an organization that holds or has applied for patents relating to the content of the manuscript?
- Do you have any other financial competing interests?
- Do you have any non-financial competing interests in relation to this paper?

If you can answer no to all of the above, write 'I declare that I have no competing interests' below. If your reply is yes to any, please give details below.

I declare that I have no competing interests

I agree to the open peer review policy of the journal. I understand that my name will be included on my report to the authors and, if the manuscript is accepted for publication, my named report including any attachments I upload will be posted on the website along with the authors' responses. I agree for my report to be made available under an Open Access Creative Commons CC-BY license (http://creativecommons.org/licenses/by/4.0/). I understand that any comments which I do not wish to be included in my named report can be included as confidential comments to the editors, which will not be published.

Choose an item.

To further support our reviewers, we have joined with Publons, where you can gain additional credit to further highlight your hard work (see: https://publons.com/journal/530/gigascience). On publication of this paper, your review will be automatically added to Publons, you can then choose whether or not to claim your Publons credit. I understand this statement.

Yes Choose an item.
