# Supplementary material for: Multi-dimensional machine learning approaches for fruit shape phenotyping in strawberry
Source: Gigascience. 2020 Apr 30;9(5):giaa030. doi: 10.1093/gigascience/giaa030 (PMC7191992; doi:10.1093/gigascience/giaa030)
Supplement: giaa030_Reviewer_2_Report_Original_Submission — Mao Li -- 10/4/2019 Reviewed [file giaa030_reviewer_2_report_original_submission.pdf]

Reviewer Report

Title: Multi-Dimensional Machine Learning Approaches for Fruit Shape Phenotyping in Strawberry

Version: Original Submission **Date:** 10/4/2019

Reviewer name: Mao Li

Reviewer Comments to Author:

This is the review for "Multi-Dimensional Machine Learning Approaches for Fruit Shape Recognition and Phenotyping in Strawberry" by Feldmann et al. The authors developed an unsupervised clustering approach named the principal progression of k clusters (PPKC) which provides ordinal categories for 2D digital images. This clusters are ordered based on the rank of the PC1 loading of PCA performing on a covariance matrix. This matrix is derived from rectangular matrix in which each element is the proportion of each cluster came from all former clusters derived from a modified k-means clustering. Then they did very thorough analysis including calculate broad-sense heritability, extract features derived from four different types of shape descriptors, study their relationships and perform classification using a subset of features. The manuscript is well written and the analysis is performed thoroughly.

My review will mainly focus on the method and data analysis part. I personally appreciate all kinds of different new methods. The idea of this method is interesting and also relatively easy in computation. For the current version of the manuscript, I still have a few concerns and suggestions that would like to discuss with the authors.

* The main concern I am having is about the Modified k-clustering (line 115-149) which is also the very early step of the analysis. The authors observed there are two groups (c2 and c6) are mirror symmetric when they performed k (=8)-mean clustering. Thus they rotated the images in one of these two groups and redo the k-mean clustering. In fact, if we "observe an object from two opposite sides, they would appear as mirror images of one another". But this is true for all the images, not just the one in group c6. And the original dataset includes the random factor. Assume there is a direction reflecting the mirror symmetry. Only rotating the images in c6 means only move the most extreme data points into the other most extreme side. This will result in partially reducing the variance caused by the mirror symmetry. However, it will also change the whole data structure and pairwise distance. For most empirical data, it is very common to have relatively continuous distribution. I believe it is also the case for this strawberry data. For different k, the samples that closer to group c6 might be grouped together with some of the samples in c6. But after partially rotate c6, those samples will be far from them. I am not sure whether it is valid to do it this way. What if PPKC is performed on the original dataset? Will the result be very different? I think it is more careful to eliminate the mirror symmetry for the whole dataset not just partially, or just using the original dataset without stratification.

* I do have a few questions about the new methods PPKC.

a). With the context and math equations, I understand the method itself such as the math and the algorithm. But I still had hard time to interpret and understand how it works. If I understand correctly, the PCA treats the k clusters as variables and each former groups as the observations. The PC1 loadings

calculate the contributions of these k clusters to the most variance. But I am still not sure how it can reflect the progression categories. I think it is even challenged for the readers who may not be familiar with PCA. Thus, I think, beside those math theory, it would be better to provide more intuitive interpretation.

b). The reason why the PPKC in k=8 did not follow the same pattern is not convinced me as I didn't see the "dramatic change of slop in the total within-group sums of squares, AIC , and Adjusted R2" (line 206-208) at k=8. Is the k going to be different number for different dataset? Is the method not stable enough?

c). For a new method, the readers usually would like to know whether there are other methods have been developed for the similar purpose. What are the advantage and limitation of this new approach. (Line 39-41) Is it the first method that is able to provide ordinal categories? If not, the comparison with some previous method is commonly asked.

Minor points:

1. Line 23-24, this sentence is same with the one in the abstract. Please rephrase either of them.
2. The sentences from line 86-98 provides the outline of this work. But maybe because there are many different steps with many details to process the images, extract features, machine learning, clustering and classification, readers may get lost. I think a workflow for the entire analysis from input raw data to the output analysis result with the details will better guide the readers.
3. Line 136 and 138, there is no Fig. S1D and Fig. S1E in the figure.
4. Line 158, AIC and BIC are abbreviation. I think it would be better to give the full phrase when they are mentioned in the first place.
5. Line 171, just curious how to compute the phenotypic variance for the ordinal scales.
6. Equation (1), the denominators of the last row are typos.
7. Line 239, same comment for OOB as minor point 3.
8. Line 610, same comment for PVE as minor point 3.
9. What is the computational time?

Methods

Are the methods appropriate to the aims of the study, are they well described, and are necessary controls included? Choose an item.

Conclusions

Are the conclusions adequately supported by the data shown? Choose an item.

Reporting Standards

Does the manuscript adhere to the journal's guidelines on minimum standards of reporting? Choose an item.

Choose an item.

Statistics

Are you able to assess all statistics in the manuscript, including the appropriateness of statistical tests used? Choose an item.

Quality of Written English

Please indicate the quality of language in the manuscript: Choose an item.

Declaration of Competing Interests

Please complete a declaration of competing interests, considering the following questions:

- Have you in the past five years received reimbursements, fees, funding, or salary from an organisation that may in any way gain or lose financially from the publication of this manuscript, either now or in the future?
- Do you hold any stocks or shares in an organisation that may in any way gain or lose financially from the publication of this manuscript, either now or in the future?
- Do you hold or are you currently applying for any patents relating to the content of the manuscript?
- Have you received reimbursements, fees, funding, or salary from an organization that holds or has applied for patents relating to the content of the manuscript?
- Do you have any other financial competing interests?
- Do you have any non-financial competing interests in relation to this paper?

If you can answer no to all of the above, write 'I declare that I have no competing interests' below. If your reply is yes to any, please give details below.

I declare that I have no competing interests

I agree to the open peer review policy of the journal. I understand that my name will be included on my report to the authors and, if the manuscript is accepted for publication, my named report including any attachments I upload will be posted on the website along with the authors' responses. I agree for my report to be made available under an Open Access Creative Commons CC-BY license (http://creativecommons.org/licenses/by/4.0/). I understand that any comments which I do not wish to be included in my named report can be included as confidential comments to the editors, which will not be published.

Choose an item.

To further support our reviewers, we have joined with Publons, where you can gain additional credit to further highlight your hard work (see: https://publons.com/journal/530/gigascience). On publication of this paper, your review will be automatically added to Publons, you can then choose whether or not to claim your Publons credit. I understand this statement.

Yes Choose an item.
