# Supplementary material for: Multi-dimensional machine learning approaches for fruit shape phenotyping in strawberry
Source: Gigascience. 2020 Apr 30;9(5):giaa030. doi: 10.1093/gigascience/giaa030 (PMC7191992; doi:10.1093/gigascience/giaa030)
Supplement: giaa030_Reviewer_2_Report_Revision_1 — Mao Li -- 12/5/2019 Reviewed [file giaa030_reviewer_2_report_revision_1.pdf]

**Reviewer Report**

**Title: Multi-Dimensional Machine Learning Approaches for Fruit Shape Phenotyping in Strawberry**

**Version: Revision 1** **Date:** 12/5/2019

**Reviewer name: Mao Li**

**Reviewer Comments to Author:**

This is the review of the revised version of "Multi-Dimensional Machine Learning Approaches for Fruit Shape Phenotyping in Strawberry". The reviewer was the reviewer #2 of the original submission.
This work reported new approaches. Thus, I paid a lot attention to make sure the method makes sense and the analysis is validated. Thus I raised two main concerns about 1) an important preprocessing (modified k-clustering at k=8) step and 2) the reason that the new method (PPKC) doesn't not follow the pattern at k=8. But I am afraid that my concerns are still not fully removed especially the first one. Roughly speaking, the modified k-clustering is performing by a few steps: 1) display the categories of k mean clustering for k=2 to 10; 2) visually find, at k=8, there were two categories similar but left-right mirror symmetric; 3) left-right flip the image for one of these two mirror categories to make these two categories both skew to the right. I agree that it makes sense to eliminate the left-right symmetry as it is not biological relevant feature. But my concern is this is a partial manipulation. I agree that, at k=8, "there was typically symmetry to most of the discovered categories" except these two categories. But I think this is because k is not large enough to pick more detailed features. Just like, those mirror symmetrical categories only appeared after k is increased to be large enough (k=8) as more detailed features had been considered. I guess there might be some k (maybe larger than 10) existed that another two categories will be picked showing their difference in skewness (although I believe the skewness should not be as obvious as the ones at k=8). Should we also flip one of these categories? I have this concern because the current processing only flipped the images with large skewness. I believe a more safe manipulation is to standardize this process so that all images skew to the right. However, I understand it might be too much to do this as the authors have to redo everything. Maybe an easy accessible way is to just test whether this partial manipulation is somehow safe to do.
Let's think about the way to test it. The authors may have better ideas. Here, I can provide one possible option. How about examining the k mean clustering for a few larger k values (e.g. k=10 to 20) on the current working data (k8{c6} has been flipped already) and visually see if there are two more categories similar but left-right mirror symmetric at some k. If no (or yes, but almost cannot notice by human eyes), then I think the current analysis should be safe enough because the data after this partial manipulation is similar as, when taking the picture, human purposely standardize the strawberry side to be right skew by our eyeballs. If yes but noticeable, the work that they need to do might be a little bit more. For example, flipping one of these categories and perform the some analysis to see whether the result will be changed. But no matter which case, I think it would be better to clarify and discuss more in the text for the readers.
For the reason that KKPC did not follow the pattern at k=8. Authors were not specifically concerned as the model was highly overfit. I am fine with this reason. But since it also happened at k=8, that is why I

am a little curious whether it is somehow related to the process in the modified k-clustering. I will let the authors decide whether they would like to explore a bit or clarify a bit more for the readers.

**Methods**

Are the methods appropriate to the aims of the study, are they well described, and are necessary controls included? Choose an item.

**Conclusions**

Are the conclusions adequately supported by the data shown? Choose an item.

**Reporting Standards**

Does the manuscript adhere to the journal's guidelines on minimum standards of reporting? Choose an item.

Choose an item.

**Statistics**

Are you able to assess all statistics in the manuscript, including the appropriateness of statistical tests used? Choose an item.

**Quality of Written English**

Please indicate the quality of language in the manuscript: Choose an item.

**Declaration of Competing Interests**

Please complete a declaration of competing interests, considering the following questions:

- Have you in the past five years received reimbursements, fees, funding, or salary from an organisation that may in any way gain or lose financially from the publication of this manuscript, either now or in the future?
- Do you hold any stocks or shares in an organisation that may in any way gain or lose financially from the publication of this manuscript, either now or in the future?
- Do you hold or are you currently applying for any patents relating to the content of the manuscript?
- Have you received reimbursements, fees, funding, or salary from an organization that holds or has applied for patents relating to the content of the manuscript?
- Do you have any other financial competing interests?
- Do you have any non-financial competing interests in relation to this paper?

If you can answer no to all of the above, write 'I declare that I have no competing interests' below. If your reply is yes to any, please give details below.

I declare that I have no competing interests

I agree to the open peer review policy of the journal. I understand that my name will be included on my report to the authors and, if the manuscript is accepted for publication, my named report including any attachments I upload will be posted on the website along with the authors' responses. I agree for my report to be made available under an Open Access Creative Commons CC-BY license (http://creativecommons.org/licenses/by/4.0/). I understand that any comments which I do not wish to be included in my named report can be included as confidential comments to the editors, which will not be published.

Choose an item.

To further support our reviewers, we have joined with Publons, where you can gain additional credit to further highlight your hard work (see: https://publons.com/journal/530/gigascience). On publication of this paper, your review will be automatically added to Publons, you can then choose whether or not to claim your Publons credit. I understand this statement.

Yes Choose an item.
