# Supplementary material for: Multi-dimensional machine learning approaches for fruit shape phenotyping in strawberry
Source: Gigascience. 2020 Apr 30;9(5):giaa030. doi: 10.1093/gigascience/giaa030 (PMC7191992; doi:10.1093/gigascience/giaa030)
Supplement: giaa030_Reviewer_3_Report_Revision_2 — Chris Armit -- 2/4/2020 Reviewed [file giaa030_reviewer_3_report_revision_2.pdf]

Reviewer Report

Title: Multi-Dimensional Machine Learning Approaches for Fruit Shape Phenotyping in Strawberry

Version: Revision 2 **Date:** 2/4/2020

Reviewer name: Chris Armit

Reviewer Comments to Author:

I was invited to review this Research Article as it had been noted that the image dataset that was originally submitted to GigaScience, and that were used in the initial review, were now to be replaced by an alternative and updated image dataset that has been made publicly available in Zenodo.
By comparing md5 checksum values of the image data submitted to GigaScience with those submitted to Zenodo, I can report that only 8 of the original 16,672 images that were submitted to GigaScience are missing from Zenodo and that 7 of these have been replaced with alternative images with the same filename but different md5 checksum values. The different md5 values are listed below.
Files submitted to GigaDB, but not Zenodo
md5 Filename
6136777d7556c8c7f0ccec4d7a2d5465 ./raw_H2/3240_16C051P038.JPG
00bd8c7412cac407acdb305cbc7a7da3 ./raw_H2/3241_16C095P027.JPG
125a20a0e39a88a3ffbe58d087a5102e ./ReSized_Images/4924_mN_2241_16C052P019.jpg
9e49be370ca167d5037698fc36211caa ./ReSized_Images/3603_mN_1197_16C095P014.jpg
8e3709b7030d57be90785397a045f129 ./ReSized_Images/6654_mN_3465_16C088P003.jpg
6ece9586a1c756f927757575068b44be ./ReSized_Images/627_mN_1356_16C059P024.jpg
8055713ffb0c77c857caea1e93b29776 ./ReSized_Images/3418_mN_1106_16C089P008.jpg
577a08c11e1ecf99351841074fa3dac5 ./ReSized_Images/4375_mN_1574_16C057P040.jpg
Files submitted to Zenodo, but not GigaDB
md5Filename
9c5a9c4c19475603544e8d89e481c5a8 ./Images/H2/3240_16C051P038.JPG
a83e9a8db567997222ebb179c77242ae ./Images/H2/3241_16C095P027.JPG
eca07800c41d60c81ba065b6f1a6a58f ./Images/ReSized/4924_mN_2241_16C052P019.jpg
cf6c5adea524c3e9cc9649b05c6ff011 ./Images/ReSized/3603_mN_1197_16C095P014.jpg
3a9f5fff272b23e389afefb2e77132b6 ./Images/ReSized/6654_mN_3465_16C088P003.jpg
265af22fd509f7c2ebe6bd2429779732 ./Images/ReSized/627_mN_1356_16C059P024.jpg
995025035c9f955713737473c361aab3 ./Images/ReSized/3418_mN_1106_16C089P008.jpg
As there are only a small number of updated image files I recommend this article for publication in GigaScience. I additionally recommend that the GigaScience Database (GigaDB) takes a snapshot of the image data archived in Zenodo.

Methods

Are the methods appropriate to the aims of the study, are they well described, and are necessary controls included? Choose an item.

Conclusions

Are the conclusions adequately supported by the data shown? Choose an item.

Reporting Standards

Does the manuscript adhere to the journal's guidelines on minimum standards of reporting? Choose an item.

Choose an item.

Statistics

Are you able to assess all statistics in the manuscript, including the appropriateness of statistical tests used? Choose an item.

Quality of Written English

Please indicate the quality of language in the manuscript: Choose an item.

Declaration of Competing Interests

Please complete a declaration of competing interests, considering the following questions:

- Have you in the past five years received reimbursements, fees, funding, or salary from an organisation that may in any way gain or lose financially from the publication of this manuscript, either now or in the future?
- Do you hold any stocks or shares in an organisation that may in any way gain or lose financially from the publication of this manuscript, either now or in the future?
- Do you hold or are you currently applying for any patents relating to the content of the manuscript?
- Have you received reimbursements, fees, funding, or salary from an organization that holds or has applied for patents relating to the content of the manuscript?
- Do you have any other financial competing interests?
- Do you have any non-financial competing interests in relation to this paper?

If you can answer no to all of the above, write 'I declare that I have no competing interests' below. If your reply is yes to any, please give details below.

I declare that I have no competing interests.

I agree to the open peer review policy of the journal. I understand that my name will be included on my report to the authors and, if the manuscript is accepted for publication, my named report including any attachments I upload will be posted on the website along with the authors' responses. I agree for my

report to be made available under an Open Access Creative Commons CC-BY license (http://creativecommons.org/licenses/by/4.0/). I understand that any comments which I do not wish to be included in my named report can be included as confidential comments to the editors, which will not be published.

Choose an item.

To further support our reviewers, we have joined with Publons, where you can gain additional credit to further highlight your hard work (see: https://publons.com/journal/530/gigascience). On publication of this paper, your review will be automatically added to Publons, you can then choose whether or not to claim your Publons credit. I understand this statement.

Yes Choose an item.
